# Beyond MIDAS: An *In Silico* Study of a Putative Noncanonical C16 Binding Site in αvβ3 Integrin

**DOI:** 10.1021/acsomega.5c11287

**Published:** 2026-02-19

**Authors:** Francisco Das Chagas Pereira de Andrade, Yago Ferreira e Silva, Paulo Ricardo Batista, Anderson Nogueira Mendes

**Affiliations:** † Laboratory of Innovation in Science and Technology − LACITEC, Department of Biophysics and Physiology, 67823 Federal University of Piauí, Teresina, Piauí 64049-550, Brazil; ‡ Department of Biophysics and Physiology, Federal University of Piauí, Teresina 64049-550, Brazil; § Programa de Computação Científica, Vice-Presidência de Educação, Informação e Comunicação, 37903 Fundação Oswaldo Cruz, Av. Brasil 4365, Residência Oficial, Manguinhos, Rio de Janeiro 21045-900, Brasil; ∥ Programa de Pós-graduação em Biologia Computacional e Sistemas, Instituto Oswaldo Cruz, Fundação Oswaldo Cruz, Av. Brasil 4365, Manguinhos, Rio de Janeiro 21045-900, Brasil

## Abstract

Integrins are essential transmembrane receptors that mediate bidirectional signaling between the extracellular matrix (ECM) and the cytoskeleton. Among them, αvβ3 and α5β1 are key regulators of angiogenesis, metastasis, and tumor progression. The laminin-1-derived C16 peptide (KAFDITYVRLKF) interacts with both integrins, but the molecular determinants governing its specificity remain unclear. Here, we applied an integrative computational approach combining ADMET predictions, molecular docking, and all-atom molecular dynamics (MD) simulations to characterize C16 pharmacokinetics and its predicted binding modes to αvβ3 and α5β1. *In silico* pharmacological profiling indicated that C16 displays low oral bioavailability and limited permeability but possesses favorable binding properties and moderate metabolic stability. Docking and MD analyses revealed that C16 interacts with the canonical metal ion–dependent adhesion site (MIDAS) in both integrins via conserved hydrogen bonds and hydrophobic contacts. Strikingly, an additional putative noncanonical binding pocket (S2) was identified in αvβ3 (not in α5β1), located opposite the MIDAS. This site forms an amphiphilic interface stabilized by complementary electrostatic and hydrophobic interactions, providing a structural rationale for the preferential interaction of C16 with αvβ3. MD simulations confirmed the stability of both S1 and S2 complexes, with reduced conformational fluctuations and lower binding free energies for αvβ3, particularly at the S2 site. These computational findings propose a novel αvβ3 C16 recognition mode, offering computational insights into predicted molecular determinants of integrin subtype selectivity and informing the rational design of peptide-based therapeutics targeting angiogenesis and tumor invasion.

## Graphical abstract

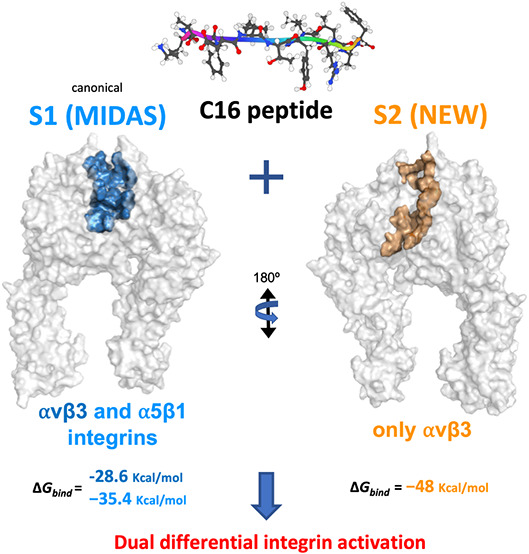

## 1 Introduction

Integrins are heterodimeric transmembrane receptors that mediate bidirectional communication between the extracellular matrix (ECM) and the intracellular cytoskeleton. They play a central role in the cell’s mechanical stability and signal transduction.^1,2^ Specifically, human integrins are composed of 18 α and 8 β subunits, which can combine to form 24 unique heterodimers with distinct affinities for ECM ligands.^3–5^ These interactions may regulate essential cellular processes, including adhesion, migration, proliferation, and survival.^6^

Structurally, each integrin subunit comprises a large extracellular domain (approximately 1,000 residues), a single transmembrane helix, and a short cytoplasmic tail (∼20 residues).^7^ The extracellular segment of integrins is organized into two major portions: the headpiece, which contains the ligand-binding domains, and the legs, which connect to the transmembrane and cytoplasmic tails.^8^ The headpiece represents the functional core of the receptor (Figure S1), as it houses the structural elements responsible for ligand recognition and affinity regulation. It is formed by the N-terminal portions of the α and β subunits. The α subunit typically includes a β-propeller domain and, in many integrins, an inserted I domain (also known as the αA domain) that directly mediates ligand recognition.^9^ The I domain contains a metal ion–dependent adhesion site (MIDAS), which coordinates divalent cations crucial for stabilizing ligand interactions. The β subunit contributes an I-like domain that pairs with the α subunit to complete the headpiece architecture and also contains a MIDAS motif. In addition, the β subunit head domain harbors other conserved metal-binding motifs, including the adjacent MIDAS (ADMIDAS) and the Synergistic Metal ion-Binding Site (SyMBS), which fine-tune ligand specificity and affinity.^10^ These structural elements are essential for integrin activation and are important pharmacological targets for therapeutics for integrin-associated disease.^11–14^

Integrins undergo conformational changes between at least three conformational states: a bent-closed (inactive), an extended-closed (low-affinity), and an extended-open (high-affinity).^15^ In the bent-closed state, the headpiece is folded toward the membrane, limiting access to ligands. Upon activation, integrins shift to an extended-closed conformation, in which the extracellular domain straightens but the headpiece remains in a low-affinity configuration. The final transition to an extended-open state involves a structural rearrangement of the headpiece, exposing the ligand-binding site in a high-affinity form.^2^ This conformation is crucial for effective ligand engagement and stable cell adhesion.^16,17^ Ligand binding triggers these conformational shifts, which are transmitted through the transmembrane region to the cytoplasmic tails—a process termed integrin activation. Such conformational plasticity enables integrins to function as bidirectional signaling molecules.^5,18^

Antagonists against isoforms αIIbβ3, α4β1, α4β7, and αLβ2 have been approved for the treatment of thrombosis, inflammatory diseases, and dry eye syndrome.^15^ Beyond these clinical advances, more than 90 additional integrin-targeting compounds (including small molecules and peptide-based mimetics) are undergoing clinical investigation.^6^

Notably, αvβ3 and α5β1 are key targets in angiogenesis, metastasis, and tissue remodeling in tumor microenvironments.^19–22^ These two integrins interact with ECM ligands via a conserved RGD motif.^23–26^ Experimental antagonism of these receptors has demonstrated efficacy in preclinical cancer models by disrupting neovascular attachment and inducing apoptosis in tumor-associated endothelial cells.^27–29^

One important component of ECM is laminin-1. The C16 peptide (KAFDITYVRLKF), derived from its γ1 chain, has been shown to interact specifically with the integrins αvβ3 and α5β1.^30–36^ Early affinity chromatography and blocking-antibody experiments identified these integrins as key receptors, and subsequent angiogenesis studies confirmed that C16 promotes endothelial migration and vascular remodeling via this dual engagement.^33^ More recent work in cancer and skin models further highlights C16’s dependence on β1-containing integrins, with functional evidence implicating α5β1 in processes such as invadopodia formation, fibronectin signaling, and inflammatory responses.^36,37^ This peptide facilitates tumor cell adhesion, invasion, and angiogenesis, suggesting it may be considered a candidate for integrin-targeted therapeutic development. Despite these findings, no rigorous quantitative binding measurements or structural analyses have been reported, leaving the precise molecular determinants and atomic-level mechanisms of C16–integrin recognition unresolved.

In this study, we adopt an integrative computational strategy to investigate the pharmacokinetic and pharmacodynamic profiles of the C16 peptide. We evaluate its drug-likeness through absorption, distribution, metabolism, excretion (ADME), and toxicity (ADMET) predictions and explore its binding affinity and dynamics with αvβ3 and α5β1 integrins using molecular docking and all-atom molecular dynamics (MD) simulations. Binding free energies are estimated using the MM-PBSA method, with particular attention to the MIDAS region’s role in coordinating ligand interactions. In addition to the canonical binding site for C16, we found a previously uncharacterized putative binding site specific in αvβ3, which can be targeted to develop new specific therapies. Collectively, these insights aim to inform rational design strategies for integrin-targeted anticancer therapeutics.

## 2 Methods

### 2.1 Predictions of C16 Physicochemical, Pharmacokinetic, Pharmacodynamic, and ADMET Properties

Physicochemical, pharmacokinetic, pharmacodynamic, and ADMET properties predictions were evaluated *in silico* analysis employing four *online* servers: PreADMET,^38^ SwissADME,^39^ ADMETlab 2.0,^40^ and ADMETSAR 2.0.^41^ These tools were selected based on prior validation reported in the literature. The results obtained from each predictor were compared to assess consistency and reliability. The three-dimensional (3D) structure of the C16 peptide (Figure 1) was extracted from the Protein Data Bank (PDB),^42^ specifically from the laminin-1 γ1 chain crystal structure (PDB ID: 4AQT^43^).

**Figure 1:**
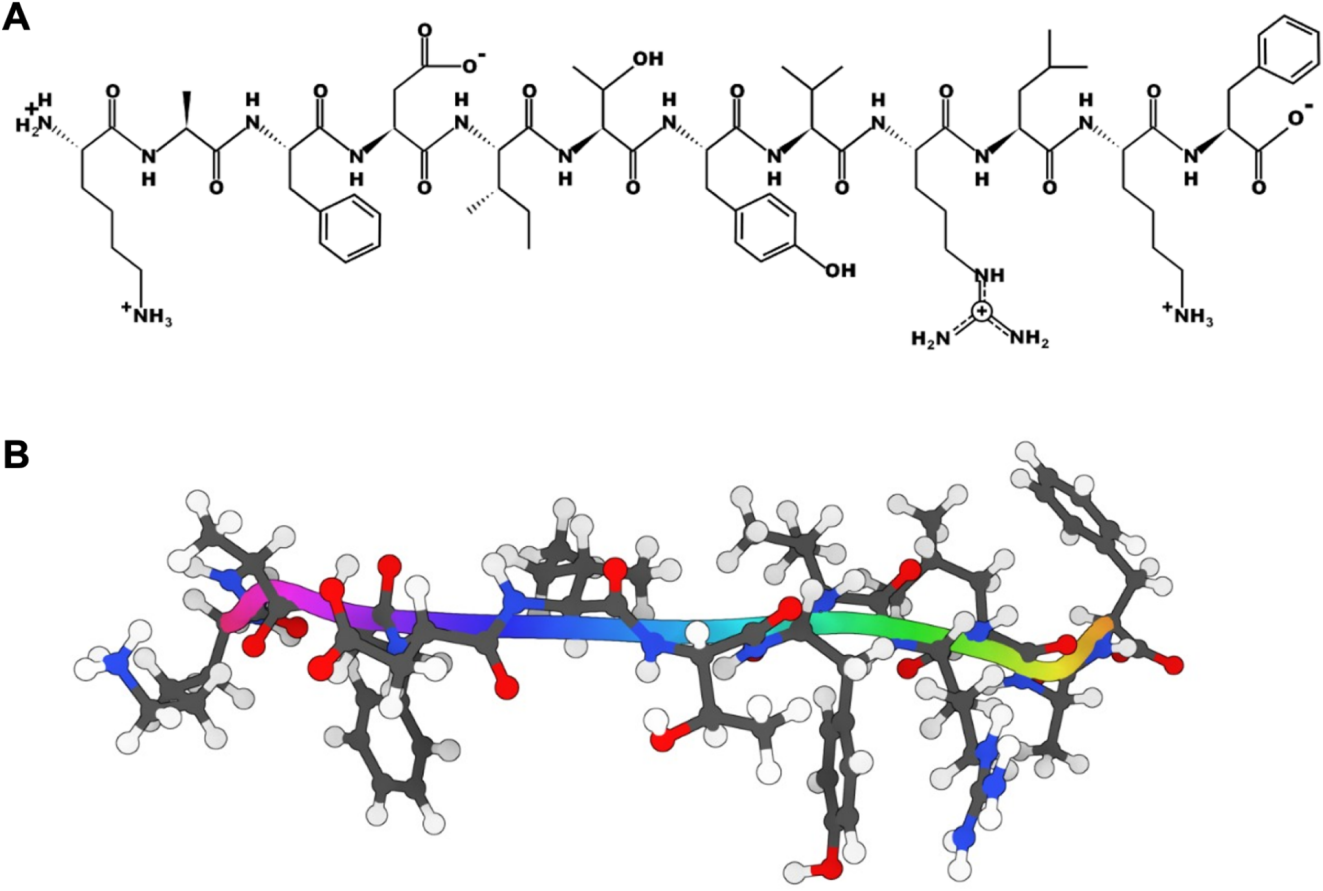
Molecular Structure of the C16 peptide. (A) Chemical structure of the C16 peptide generated from its primary amino acid sequence using the PepDraw tool. (B) Three-dimensional structural representation of the C16 peptide obtained from the corresponding laminin-1 γ1 chain fragment experimental structure. Visualization and rendering performed with SAMSON 2022 R.^44^

ADMET properties of the C16 peptide were predicted using four independent web servers in order to obtain a consensus assessment. This multipredictor strategy was adopted to reduce biases associated with individual models, which are largely optimized for small molecules rather than peptides. Predictions not consistently supported across platforms were considered outliers and were not overinterpreted.

### 2.2 Molecular Docking of C16 Peptide with Integrin Receptors

The interactions and binding interface between the C16 peptide and integrin receptors αvβ3 and α5β1 were predicted using molecular docking simulations performed with HPEPDOCK,^45^ a web-based hierarchical flexible peptide docking protocol that incorporates MODPEP^46^ for peptide conformational sampling. Unlike grid-based docking approaches, HPEPDOCK uses a fragment-based sampling strategy with an internally defined, knowledge-based scoring function, and does not allow user control over search space dimensions, scoring weights, or sampling parameters. The integrin receptors were treated as rigid, while peptide flexibility was implicitly accounted for during docking. The number of output poses was set to 100 for each run.

The docking process followed a two-step approach: *i.* an initial blind docking was performed to identify potential binding sites; and; *ii.* a subsequent directed docking was conducted using the predicted interaction sites in the blind docking step.

The coordinate files and sequences for the headpiece portion of integrin αvβ3 and α5β1 structures were retrieved from the PDB (PDB IDs: 4MMX^47^ and 3VI4,^48^ respectively) and UniProt.^49^ Missing residues in the protein structures were identified through sequence alignment using EMBOSS Needle^50^ and modeled using MODELER 10.4.^51^ The sequence alignment of the headpiece portion from each α and β subunits (corresponding to A and B chains, respectively) is provided in Figure S2. The stereochemical quality of the final receptor structures was assessed and validated through Ramachandran plot analysis (Figure S3) and the programs ERRAT2 ^52^ and VERIFY3D^53,54^ using the SAVES v.6.1 Web server.^55^ The *phi* and *psi* dihedral distributions were analyzed using the PyRAMA script.^56,57^

An initial blind docking was performed for each integrin receptor, allowing the C16 peptide to explore the entire protein surface without predefined constraints. The resulting poses were grouped by clustering analysis, and binding regions were identified based on the most populated clusters rather than individual docking scores.

For αvβ3, two dominant binding regions were identified: the most populated cluster, corresponding to a noncanonical interdomain pocket (S2), and the second most populated cluster, corresponding to the canonical MIDAS-associated site (S1) (Figure S4). For α5β1, only MIDAS-associated poses formed stable and recurrent clusters. For each site, the best-scored pose within the corresponding cluster was selected.

The interacting residues identified in these poses were then used as spatial restraints for a second round of directed docking, refining peptide orientation within the previously identified binding regions. They are **α5β1 S1** (A:224, A:225, A:227, A:228, A:254, A:255, A:257, A:258, B:728, B:729, B:730, B:733, B:782, B:783, B:819, B:820, B:821, B:822, B:823, B:824, B:825, B:855, B:856, B:857, B:858, B:914, B:916, B:917, B:919, B:920, B:938); **αvβ3 S1** (A:119, A:215, A:216, A:218, A:219, A:121, A:122, A:148, A:149, A:154, A:245, A:248, A:249, B:1124, B:1128, B:1132, B:1135, B:1170, B:1171, B:1172, B:1208, B:1209, B:1121, B:1122, B:1123, B:1270, B:1273, B:1174, B:1269, B:1270, B:1273); and **αvβ3 S2** (A:88, A:90, A:91, A:110, A:111, A:113, A:122, A:406, A:337, A:339, A:364, A:369, B:1117, B:1118, B:1219, B:1220, B:1123, B:1219, B:1221, B:1222, B:1223, B:1226, B:1228, B:1239, B:1240, B:1241, B:1243). The S1 site is located near to the MIDAS while the S2 is located at the opposite side of the integrin.

C16 peptide structure files were prepared using Discovery Studio 2020,^58^ ensuring flexibility of all rotatable bonds. Prior to submission to HPEPDOCK, all structures (integrins + ions + C16) were protonated according to a pH of 7.4 using the APBS (Adaptive Poisson–Boltzmann Solver) server,^59^ with the *propka* titration method and the PARSE force field. This pH setting ensures consistency with typical extracellular conditions under which integrin–ligand interactions occur.

To evaluate the robustness and specificity of our docking approach, we performed two additional control experiments using peptides that preserve the amino-acid composition and net charge of C16 but alter its sequence order: (i) a reversed C16 sequence (FKLRYVITDFAK) and (ii) a scrambled C16 variant (KRYLAFVKTDFI) in which the same residues are rearranged into a randomized order. These controls allow us to determine whether the predicted binding mode arises from sequence-specific recognition rather than generic physicochemical properties. Both control peptides were blind docked into the αvβ3 integrin using the same workflow applied to the native C16 peptide.

To analyze the docking results, identify interacting residues, and characterize electrostatic and van der Waals interactions, the open-source version of PyMOL 2.1.1 ^60^ and BIOVIA Discovery Studio 2021 ^58^ were used. PyMOL was also employed to inspect the final complex structures. Two-dimensional and three-dimensional peptide-integrin interaction diagrams were generated using LigPlot^61^ and SAMSON, respectively.

### 2.3 Molecular Dynamics Simulations

To evaluate the stability of the protein-peptide association at the molecular level and to obtain a comprehensive picture of how predicted native contacts are maintained, molecular dynamics (MD) simulations studies were conducted.^62^

The top-scored docking pose of each protein-peptide complex was selected for the MD simulations. The simulation systems were set up for each protein–ligand complex in solution using the GPU-enabled GROMACS 2024.2 package.^63^ Besides the predicted C16 peptide/integrin complexes, the *apo* αvβ3 and α5β1 integrins were also simulated. Each simulation system was carried out in triplicate, with new velocities randomly sorted during the heating step.

All necessary topology files were generated using the Solution Building module of the CHARMM-GUI web-based interface.^64^ CHARMM-GUI was used to build the simulation system and to obtain the scripts and topology files compatible with GROMACS. TIP3P water model was used to solvate the system. The dimensions of the box were defined by ensuring at least a 10 Å distance between the protein and the box edges to avoid boundary effects. Proper neutralization of the system was achieved by adding counterions (Na^+^ and Cl^–^) based on the protein’s charge automatically calculated by CHARMM-GUI. CHARMM36 force field^65^ was chosen, which provides parameters for proteins and peptides.

Before the production of MD simulations, structure optimization by energy minimization was performed, to avoid unfavorable contacts or steric clashes in the system. The steepest-descent energy minimization was used, and the position of heavy atoms were restrained, with a maximum force of 100 kJ/(mol·nm) on each atom. The solvated system was equilibrated in two steps: first, the system was equilibrated for 1 ns under a constant volume ensemble (NVT) without restraints applied; second, the system was equilibrated for another 1 ns under a constant pressure ensemble (NPT) without any restraint. Production simulation was conducted for 100 ns under the NPT ensemble. All bonds containing hydrogen atoms were constrained using the default LINCS constraint algorithm. The coupling algorithm of Nose-Hoover was used to maintain temperature (310 K) and Parrinello–Rahman algorithm to maintain temperature pressure (1 atm) with a constant of 1.0 ps. The electrostatic interactions were treated with the particle mesh-Ewald (PME) method. The integration time step was set to 2 fs and periodic boundary conditions were applied in all directions.

### 2.4 MD Trajectory Analyses

Global structural parameters were quantified using utilities from the GROMACS 2024.5 package. The trajectories and configuration files are available in the linked Zenodo and GitHub repositories.

#### 2.4.1 Trajectory Preparation

An index file was generated with atoms corresponding to the integrin α and β chains and the C16 peptide using gmx make_ndx. Trajectories were processed with gmx trjconv to remove periodic boundary conditions and recenter the integrins.

#### 2.4.2 Root Mean Square (RMS) Deviation

RMSD values were computed with gmx rms, using the first frame as reference. Calculations were performed separately for the integrins, the C16 peptide, and the peptide relative to the integrins.

#### 2.4.3 RMS Fluctuation

RMSF profiles of the integrins and the C16 peptide were obtained with gmx rmsf.

#### 2.4.4 Radius of Gyration (Rg)

The Rg of each trajectory frame was determined using gmx gyrate.

#### 2.4.5 Solvent-Accessible Surface Area (SASA)

The SASA of the integrins was calculated using the gmx sasa module.

#### 2.4.6 Peptide Secondary Structure

The secondary structure of the C16 peptide was monitored across trajectories using gmx dssp.

#### 2.4.7 Clustering and Pairwise RMSD Distribution

Structural clustering and RMSD distribution analyses were performed with the gmx cluster using the gromos algorithm. Separate calculations were performed for the integrins (cutoff = 0.30) and for the C16 peptide (cutoff = 0.15).

#### 2.4.8 Hydrogen bond analysis

Hydrogen bonds between the C16 peptide and integrins were quantified with gmx hbond. Each pair was classified, and its occupancy evaluated using a Python script with the MDAnalysis package.

#### 2.4.9 Peptide Binding Free Energy Estimation

Binding free energies of the C16 peptide to integrins were estimated using the Molecular Mechanics/Poisson–Boltzmann Surface Area (MM-PBSA) approach, implemented with the gmx_MMPBSA tool.^66,67^ This method is based on the AMBER MMPBSA.py script and enables end-state free energy calculations from GROMACS trajectories. A brief tutorial describing how to execute the MMPBSA workflow is provided in the GitHub, together with the full mmpbsa.in input file containing all calculation parameters and settings. For each system, MM-PBSA calculations were performed on three independent simulation replicates, and the reported binding free energies correspond to the mean values ± standard deviation (SD) computed over the three replicates. A total of 1001 frames were extracted from the production MD trajectories and used for the analysis. The atomic radii were assigned according to the mbondi2 scheme (PBRadii = 3). Binding free energies were computed using the Generalized Born implicit solvent model (igb = 5), with an internal dielectric constant of 1.0, an external dielectric constant of 78.5, and a temperature of 310.15 K. No salt concentration was included in the implicit solvent calculations. The binding free energy was calculated as the sum of molecular mechanics energy terms (electrostatic and van der Waals interactions) and solvation free energy contributions. The nonpolar solvation term was estimated using a solvent-accessible surface area (SASA) model with a surface tension coefficient of 0.0072 kcal·mol^–1^·Å^–2^ and a solvent probe radius of 1.4 Å. Entropic contributions were not included in the MM-PBSA calculations. Accordingly, the reported MM-PBSA values represent relative binding free energies suitable for comparative analysis between systems rather than absolute binding affinities.

#### 2.4.10 Chain/Residue Matching

Residue correspondence between αvβ3 and α5β1 integrins was established using the matchChains function of ProDy^68^ (v. 2.3) with pairwise sequence alignment (pwalign = True) and a sequence identity threshold of 45%. This procedure yielded 1011 common residues.

#### 2.4.11 Principal Component Analysis (PCA)

PCA was performed on concatenated trajectories from all systems (considering the common residues), including replicates, to identify dominant motions. Calculations were based on Cα Cartesian coordinates of the 1011 matched residues. The covariance matrix was generated with buildCovariance, diagonalized to obtain eigenvectors with calcModes, and the first PCs were analyzed.

#### 2.4.12 Normal Mode Analysis (NMA)

Intrinsic flexibility of each integrin structure was assessed by computing vibrational modes with the elastic network model. The 100 slowest nontrivial modes were calculated with ProDy using the anisotropic network model (calcANM), based on Cα Cartesian coordinates of the 1011 common residues. The Hessian matrix was constructed with a 15 Å cutoff for pairwise interactions and a spring constant γ = 1 N/m, and modes were obtained by diagonalization.

#### 2.4.13 Deformation Vector Calculation

Structural deformation vector (defvec) between fitted αvβ3 and α5β1 integrin structures was computed with calcDeformVector in ProDy. The resulting vector was normalized using getNormed.

#### 2.4.14 Mode Overlaps

The overlap between low-frequency normal modes and either the normalized deformation vector or the principal components was computed as their inner product. Defvector analysis was performed in both directions, and all vector operations were implemented in Python with NumPy.

#### 2.4.15 Normal Mode Linear Combination

Linear combinations of the first, second, third, and fifth slowest nontrivial modes were applied to displace the αvβ3 structure toward the α5β1 conformation. Mode weights were scaled according to their overlap with the deformation vector (0.6, 0.21, 0.15, 0.25, respectively). A 50-frame trajectory was generated with a 200-fold amplification factor using a Python script with NumPy and ProDy.

### 2.5 Plot, Figures, and Animations

All figures and movies were generated using VMD^69^ (version 1.9.4a51), the open-source version of PyMol,^60^ BIOVIA Discovery Studio 2021, LigPlot,^61^ and SAMSON. The plots were generated using matplotlib,^70^ seaborn,^71^ and ProDy tools. Most of the results were presented as averages across replicas (line plots), with standard deviations represented as shaded areas using the fill_between method in Matplotlib.

## 3 Results and Discussion

### 3.1 Prediction of C16 Putative Therapeutic Targets

Although the primary aim of this work is to characterize the interaction of C16 with αvβ3 and α5β1 integrins, the identification of additional putative partners can help contextualize the peptide’s behavior across different cellular environments. C16 has been associated with diverse processes linked to integrin activation, and its functional effects may be modulated by interactions with other receptors or signaling proteins. Thus, mapping potential off-targets offers a broader mechanistic perspective and aids in interpreting C16-induced responses reported in experimental settings.

To explore these possibilities, ligand-based target prediction was carried out to estimate the most probable molecular targets of the C16 peptide from the Therapeutic Target Database. This method evaluates the similarity between C16 and known ligands to predict potential biological targets.^72,73^

The results, summarized in Table 1 and Table S1, highlight several probable targets, along with their respective ChEMBL,^74^ UniProt, and associated PDB structure IDs, prediction probabilities, and model accuracies.

**Table 1:** Ligand-Based Target Prediction for C16 Peptide^a^

Target Name	ChEMBL ID	UniProt ID	PDB ID	Probability	Model Accuracy
Neurotensin receptor 2	CHEMBL2514	O95665	–	98.52%	100%
NF-kappa-B p105 subunit	CHEMBL3251	P19838	1SVC	98.29%	96.09%
Neur. acetylcholine receptor; α4/β4	CHEMBL19075	P30926	6UR8	98.13%	100%
Delta opioid receptor	CHEMBL236	P41143	4N6H	98.31%	99.35%
Glycine transporter 2	CHEMBL3060	Q9Y345	–	97.60%	99.17%
Dipeptidyl peptidase VIII	CHEMBL4657	Q6 V1 × 1	6EOP	97.30%	97.21%
Caspase-8	CHEMBL3776	Q14790	4JJ7	96.06%	97.06%
Neuropeptide Y receptor type 2	CHEMBL4018	P49146	–	95.09%	98.94%

a Targets with model accuracy and prediction probability >95%, sorted by descending probability.

Among the predicted interactions, notable targets include the NF-kappa-B p105 subunit and peroxisome proliferator-activated receptors (PPARs), which are both critically involved in the regulation of inflammatory responses and metabolic processes. Literature reports emphasize the pivotal roles of NF-κB and PPARs in inflammation, cell proliferation, and metabolic homeostasis.^75–77^

The current findings suggest that the C16 peptide may exert bioactivity through modulation of these molecular pathways, offering insight into its potential therapeutic applications. Notably, laminin-binding integrins have been shown to play a central role in reducing the secretion of pro-inflammatory cytokines by regulating NF-κB-dependent signaling. This regulation is associated with increased activity of NF-κB transcription factors and the expression of their downstream targets. These data indicate that laminin-binding integrins contribute to the control of epithelial cell polarity and inflammation through NF-κB-dependent mechanisms.^78^

The target prediction analysis for C16 indicates high probability interactions with multiple proteins involved in neurological, oncological, inflammatory, and pain-related conditions (Table S1). Notably, neuronal acetylcholine receptor α4/β4 interactions are predicted with a probability of 98.13% and model accuracy of 100%. This target is linked to Alzheimer’s disease, aneurysm, and tobacco dependence.

For cathepsins D and L, strong interactions were predicted (probabilities 99.32–99.96%), with targets associated with multiple sclerosis, bone cancer, glioma, cancer-related pain, and solid tumors, highlighting potential implications in neurodegenerative and oncological pathways.

Delta opioid receptor-predicted interactions (98.31% probability, ∼99.35% accuracy) are associated with major depressive disorder, migraine, pain, substance use disorder, and rheumatoid arthritis, indicating possible relevance in neuromodulation, analgesia, and inflammatory pathways.

These predictions suggest that C16 could interact with targets involved in both central nervous system functions and peripheral pathologies, supporting its potential therapeutic versatility. The high model probabilities and accuracies indicate a reliable *in silico* prediction, though experimental validation is required to confirm these interactions.

### 3.2 Prediction of Pharmacological and Biophysical Properties of C16 Peptide

For a molecule to be effectively administered and efficient in drug therapy, it must reach its cellular target at a sufficient concentration and remain in a bioactive form long enough to elicit the desired biological response.^79^ In this context, computational chemistry approaches are widely employed to predict key pharmacological and pharmacokinetic ADMET properties.^80,81^

These *in silico* methods range from rule-based filters, such as Lipinski’s “Rule of Five” and Veber’s criteria, which provide quick assessments of drug-likeness, to quantitative structure–activity relationship (QSAR) models, which use statistical and machine learning algorithms to relate molecular descriptors to experimentally measured ADMET parameters. Other strategies include artificial intelligence–driven predictive platforms and physiologically based pharmacokinetic modeling, which integrates molecular properties with physiological parameters to simulate drug absorption, distribution, metabolism, and excretion in virtual patient populations.

The combination of these tools enables the early identification of potential liabilities such as poor solubility, low permeability, or high metabolic turnover, thereby reducing late-stage failures in drug development and accelerating the optimization of lead compounds.

We performed ADMET predictions for the C16 peptide, evaluating a comprehensive set of pharmacokinetic and pharmacological parameters. These included drug-likeness rules, lipophilicity, p*K*_a_, P-glycoprotein (P-gp) inhibitory activity, plasma stability, red blood cell (RBC) partitioning, metabolic stability, cytochrome P450 (CYP) enzyme inhibition, blood–brain barrier (BBB) permeability, oral bioavailability, aqueous solubility, chemical stability, plasma protein binding, and P-gp induction.^82^

The molecular weight of a peptide is calculated by summing the atomic masses of all the atoms in the peptide sequence. In the case of the C16 peptide, KAFDITYVRLKF, the theoretical molecular weight is 1501 Da. C16 molecular weight was predicted to be 1502.3 ± 0.6 Da (Table 2), which is relatively large for a peptide. Peptides with molecular weights above 1000 Da often fall outside the typical Lipinski’s rule-of-5, which guides the design of orally available drugs. This means C16 peptide may require specialized delivery methods or formulations to be effective as therapeutics. It may face challenges in gastrointestinal absorption, cellular uptake, and metabolic stability due to increased susceptibility to enzymatic degradation.

**Table 2:** C16 Peptide Predicted Physicochemical and ADMET Properties

Property	ADMETSAR	ADMETlab 2.0	PreADMET
Molecular Weight (MW)	1502.82	1501.87	–
Num. heavy atoms	–	–	–
Fraction Csp3	–	0.575	–
Volume	–	1523.87	–
Density	–	0.986	–
nHA (Acceptors)	21	34	–
nHD (Donors)	21	25	–
nRot (Rotatable bonds)	50	61	–
nRing	–	3	–
Molar Refractivity	–	–	–
MaxRing	–	6	–
nHet (Heteroatoms)	–	34	–
fChear	–	1	–
nRig	–	31	–
Flexibility	–	1.968	–
Stereo Centers	–	14	–
TPSA (Å^2^)	–	578.68	–
Pure water solubility (mg/L)	–	–	0.0038
Buffer solubility (mg/L)	–	–	322.16
Log *S* (mmol/L)	–3.654	–0.964	–3.669
Log *P*	–	–1.891	–
Log *D*	–	0.007	–

To overcome these limitations, strategies such as chemical modifications to enhance stability and permeability, specialized delivery systems (e.g., nanoparticles, liposomes), and alternative administration routes (e.g., injection, inhalation) could be employed.

Despite these challenges, larger peptides can offer important therapeutic advantages. Their extended surface area allows for more extensive and complementary interactions with target proteins, which can result in higher potency when disrupting protein–protein interactions, a class of targets often considered “undruggable” by small molecules. Polar surface area is also a critical factor in ADME,^83^ as it influences solubility and permeability,^84^ ultimately affecting passive diffusion across cell membranes—a fundamental event during drug absorption and distribution.^85^

The increased number of functional groups in larger peptides enhances the diversity and strength of noncovalent interactions, including hydrogen bonds, electrostatic interactions, and hydrophobic contacts, which improves binding affinity. A significant number of hydrogen bond donors and acceptors further supports strong interaction potential with biological molecules. Additionally, the greater structural complexity of the C16 peptide can confer higher target specificity, minimizing binding to unintended biomolecules and thereby reducing off-target effects.

This specificity not only may improve safety profiles but also allow for lower therapeutic doses, helping to mitigate systemic toxicity. In some cases, the modular nature of larger peptides enables rational incorporation of functional moieties, such as cell-penetrating sequences or targeting ligands, to further enhance pharmacological performance. Ligand flexibility, as reflected in the number of rotatable bonds, can facilitate adaptation to dynamic binding sites and improve target fit. However, excessive flexibility may incur entropic penalties upon binding and negatively affect pharmacokinetic properties, highlighting the need for a balanced structural design.

The C16 peptide shows very low water solubility, with Log *S* values around −3.6 (corresponding to ∼0.0038 mg/L in PreADMET). However, it exhibits a much higher solubility in buffer (322.16 mg/L), indicating that its dissolution can be improved in specific environments. In summary, while the peptide’s solubility is moderate and may suffice for certain delivery routes, enhancing solubility could be particularly beneficial for oral administration.^86^

C16 Log *P* (partition coefficient) values such as −1.891 indicate poor lipid bilayer permeability. This low value reflects a compound with limited lipophilicity and correspondingly low membrane permeability, pointing to potential challenges in achieving adequate bioavailability without the aid of specialized delivery strategies.^87^ Consistent with Log *P*-based predictions, C16 Log *D* estimation suggests that at physiological pH, it is likely to have low aqueous solubility and moderate lipophilicity.^88^

Absorption characteristics of the C16 peptide (Table 3) highlight its low permeability across various biological membranes. PreADMET predicts low skin permeability (−0.9388), indicating that C16 is unlikely to be effectively absorbed through the skin.^89^

**Table 3:** C16 Peptide Predicted Permeability and ADMET Properties^a^

Property	ADMETSAR	ADMETlab 2.0	PreADMET
Skin Permeability	–	–	–0.9388
Caco-2 Permeability	0.865	0.817	1.289
MDCK Permeability (nm/s)	–	Yes	0.0406
P-gp Inhibitor	Yes	No inhibitor	Inhibitor
P-gp Substrate	Yes	Substrate	–
HIA (Human Intestinal Abs.)	69.9%	<30%	0.00
*F*(20%)	–	<30%	–
*F*(30%)	–	<30%	–

a “up” = uncorrelated parameter; preferred: MW > 400, acid p*K*_a_ < 4, H-bonds >10, Log *S* > −4 mol/L, solubility >60 μg/mL, Log *D*_7_ < 1, Log *P* < 0; higher Caco-2/MDCK = better permeability; HIA ≥ 70% and *F*(20%/30%) = good oral bioavailability.

Caco-2 permeability predicted values of 0.865 (ADMETSAR), 0.817 (ADMETlab 2.0), and 1.289 (PreADMET) suggest a moderate capacity to permeate intestinal epithelial cells, with the higher PreADMET value indicating relatively better absorption potential in the gut.^90^ However, human intestinal absorption (HIA) predictions of <30% (ADMETlab, PreADMET) point to poor oral absorption, a finding consistent with ADMETlab’s prediction of <30% bioavailability.

MDCK Permeability (0.0406 nm/s) indicates very low permeability, meaning the peptide may have limited central nervous system access.^91^ ADMETlab also classified C16 as a P-gp substrate, which means that it may interact with this transporter in complex ways, potentially reducing its cellular uptake due to efflux.^92^

Overall, C16 exhibits low predicted skin permeability and limited potential for oral absorption, with moderate Caco-2 permeability values but HIA and bioavailability both below 30%. Very low MDCK permeability suggests minimal blood–brain barrier (BBB) penetration, while its classification as a P-glycoprotein substrate indicates possible efflux-mediated reduction in cellular uptake.

Distribution refers to the peptide’s transport in the body, including plasma protein binding, BBB penetration, and tissue distribution. C16 is predicted to have moderate plasma protein binding (≈30–40%), potentially limiting the fraction of free peptides available for distribution (Table 4).^93^

**Table 4:** C16 Predicted Plasma Binding and Distribution Properties^a^

Property	ADMETSAR	ADMETlab 2.0	PreADMET
PPB (Plasma Protein Binding, %)	46.1	32.89	38.15
Fu (Fraction Unbound in Plasma, %)	–	39.30	–
VD (Volume of Distribution, L/kg)	–	0.545	–
BBB (Blood–Brain Barrier) Penetration	0	Yes	0.027

a Legend: up = uncorrelated parameter.

The C16 fraction unbound (Fu) of 39.30% suggests that only about 40% of the peptide is freely available in the bloodstream, while the rest is bound to proteins.^94^ The volume of distribution (VD) of 0.545 L/kg is between 0.04 and 20 L/kg, which is considered optimal for drug distribution into tissues relative to the plasma.^95^

*In silico* predictions for BBB permeability of the C16 peptide show contrasting results. ADMETsar and PreADMET report low permeability values (0 and 0.027, respectively), suggesting it is unlikely to reach the central nervous system. On the other hand, ADMETlab 2.0 simply indicates “Yes”, implying some potential to permeate the BBB. These discrepancies highlight the variability among computational tools and the need for experimental validation to accurately assess C16 BBB penetration.

In short, CYP450 ligand interaction indicates how a compound is metabolized and whether it might inhibit or induce metabolic enzymes, directly impacting bioavailability, half-life, and safety. The interaction of the C16 peptide with cytochrome P450 enzymes (CYPs) is therefore crucial for understanding its metabolism (Table 5).

**Table 5:** C16 Predicted CYP450 Enzyme Interactions^a^

Property	ADMETSAR	ADMETlab 2.0	PreADMET
CYP1A2 inhibitor	No	No	–
CYP1A2 substrate	–	No	–
CYP2C19 inhibitor	No	No	No
CYP2C19 substrate	–	No	–
CYP2C9 inhibitor	No	No	No
CYP2C9 substrate	No	No	–
CYP2D6 inhibitor	No	No	No
CYP2D6 substrate	No	No	No
CYP3A4 inhibitor	No	No	Inhibitor
CYP3A4 substrate	Yes	No	Weakly

a Legend: up = uncorrelated parameter.

The CYP450 interaction profile of C16 indicates minimal predicted inhibition or substrate activity for most cytochrome isoforms, including CYP1A2, CYP2C19, CYP2C9, and CYP2D6, across ADMETSAR, ADMETlab 2.0, and PreADMET predictions. This suggests that C16 is unlikely to significantly interfere with the metabolism of drugs processed by these enzymes, reducing the risk of certain drug–drug interactions. However, results for CYP3A4 are more variable: ADMETSAR predicts that C16 is a substrate, while PreADMET classifies it as both a weak substrate and a potential inhibitor.

Given that CYP3A4 is responsible for the metabolism of a large proportion of clinically used drugs, these findings suggest that CYP3A4 may play a primary role in C16 metabolism, and that possible inhibitory effects—though inconsistently predicted—could influence coadministered CYP3A4-metabolized drugs. The lack of consensus between predictors underscores the need for experimental validation, particularly to confirm CYP3A4 interactions and clarify their potential impact on pharmacokinetics.^96^

C16 exhibits a moderate clearance rate and a short half-life (Table 6), indicating that it is metabolized and excreted relatively quickly. This pharmacokinetic profile suggests that frequent administration may be required to maintain sustained therapeutic effects, depending on its pharmacodynamic properties.^97^

**Table 6:** C16 Predicted Pharmacokinetic Parameters^a^

Property	ADMETSAR	ADMETlab 2.0	PreADMET
CL (Clearance Rate, mL/min/kg)	–	0.623	–
T1/2 (Half-Life, h)	–	0.927	–

a Legend: up = uncorrelated parameter.

The predicted toxicological profile of C16 (Table 7) highlights several potential safety concerns. Carcinogenicity predictions are contradictory: PreADMET indicates negative results in mice but positive results in rats, suggesting species-specific variability. Bioconcentration is predicted to be low (0.359, ADMETlab 2.0), indicating limited potential for bioaccumulation. Aquatic toxicity parameters (IGC50, LC50 for Fathead minnow, *Daphnia magna*, and Medaka) show moderate to low toxicity, suggesting limited environmental risk. Endocrine and receptor-mediated toxicity predictions indicate potential interactions with multiple nuclear receptors, including androgen receptor (NR-AR), estrogen receptor (NR-ER), aromatase, AhR, PPAR-γ, and downstream stress response pathways (SR-ARE, SR-ATAD5, SR-HSE, SR-MMP, SR-p53), all classified as active in ADMETlab 2.0. While these results do not indicate direct carcinogenicity or genotoxicity, the activation of multiple receptor and stress-response pathways may warrant further investigation.

**Table 7:** C16 Predicted Toxicological and Safety Parameters

Property	ADMETSAR	ADMETlab 2.0	PreADMET
Carcinogenicity (Mouse)	–	–	Negative
Carcinogenicity (Rat)	–	–	Positive
Bioconcentration Factor	–	0.359	–
IGC50 (*T. pyriformis*)	3.375	3.727	–
LC50FM (*Fathead minnow*)	–	4.460	4.8176
LC50DM (*Daphnia magna*)	–	6.778	6.1518
LC50MM (*Medaka*)	–	–	5.5658
Acute Oral Toxicity	3.872	–	–
Acute Algae Toxicity	–	–	8.9634
NR-AR (Androgen Receptor)	–	Active	–
NR-AR-LBD	–	Active	–
NR-AhR	–	Active	–
NR-Aromatase	–	Active	–
NR-ER (Estrogen Receptor)	–	Active	–
NR-ER-LBD	–	Active	–
NR-PPAR-γ	–	Active	–
SR-ARE	–	Active	–
SR-ATAD5	–	Active	–
SR-HSE	–	Active	–
SR-MMP	–	Active	–
SR-p53	–	Active	–
Acute Toxicity Rule	–	No alerts	–
Genotoxic Carcinogenicity Rule	–	No alerts	–
Nongenotoxic Carcinogenicity Rule	–	No alerts	–
Skin Sensitization Rule	–	Three alerts	–
Aquatic Toxicity Rule	–	No alerts	–

Skin sensitization predictions show three alerts, suggesting potential for dermal reactivity. Other acute toxicity parameters, including acute oral toxicity (3.872, ADMETSAR) and acute algae toxicity (8.9634, PreADMET), indicate moderate risk. Overall, the laminin-1 derivative C16 peptide exhibits limited drug-likeness due to its high molecular weight, substantial flexibility, and large hydrogen-bonding capacity, which contribute to strong interactions with biomacromolecules but also pose challenges in solubility, permeability, and oral bioavailability. Predicted absorption and distribution properties indicate low skin permeability, moderate Caco-2 intestinal permeability, poor human intestinal absorption (<30%), low bioavailability, very low MDCK permeability, and potential efflux via P-glycoprotein, suggesting that oral delivery may be ineffective and alternative administration routes or delivery systems could be required.

Lipophilicity predictions indicate limited membrane permeability, consistent with poor passive absorption. CYP450 profiling shows minimal inhibition for most isoforms but identifies CYP3A4 as the main metabolic enzyme, suggesting potential drug–drug interactions. Toxicity predictions reveal low carcinogenicity and bioaccumulation risk, moderate acute toxicity, and limited aquatic impact. Nonetheless, possible receptor-mediated effects (androgen, estrogen, aromatase, AhR, and PPAR-γ pathways) and three skin sensitization alerts warrant further validation. Overall, C16 exhibits favorable target interaction properties but displays pharmacokinetic constraints and safety concerns that must be addressed to optimize its therapeutic applicability.

The assessment of pharmacological and biophysical properties presented for the C16 peptide was included primarily to contextualize its molecular behavior rather than to suggest unexpected drug-likeness features. As a medium-sized, highly polar peptide, C16 exhibits physicochemical and ADMET characteristics typical of bioactive peptides reported in the literature, including limited stability and restricted biodistribution. Documenting these baseline properties is important for interpreting the mechanistic analyses that follow, as they help clarify why C16 is unlikely to function as a classical small-molecule ligand for integrins. This contextualization also reinforces that the biological effects attributed to C16 are more plausibly associated with local or receptor-mediated mechanisms rather than systemic pharmacokinetic behavior.

### 3.3 Molecular Docking Analyses of C16 to αvβ3 and α5β1 Integrin Receptors

#### 3.3.1 C16 Interactions with MIDAS Are Conserved in Both αvβ3 and α5β1 Integrins

The C16 peptide is relatively long and structurally flexible, enabling it to adopt diverse conformations depending on its environment, such as in solution or upon receptor binding. Consequently, it can transition between random coil and more ordered structures in response to solvent conditions or specific molecular interactions.

To evaluate how C16 engages integrins, we performed molecular docking in two sequential steps: i. an exploratory docking across the entire receptor surface, followed by ii. focused docking restricted to the regions identified in the initial screen. In both αvβ3 and α5β1, the blind docking consistently predicted a canonical binding site near the MIDAS, hereafter referred to as **S1** (Figure S4). This site overlaps with the classical RGD-recognition pocket, originally identified in fibronectin and later described in other ECM proteins such as vitronectin, osteopontin, and laminins.^7^

Additionally, we predicted the ternary integrin structures comprising the α and β integrin chains together with C16 using the AlphaFold3 Web server.^98^ In both αvβ3 and α5β1 integrins, the resulting models consistently positioned C16 near the canonical MIDAS, in agreement with the docking results (Figure S5).

Based on the C16/α5β1 interaction residues predicted in the initial exploratory screening, a more focused and refined docking analysis was conducted. The interactions of the top-scoring C16 conformation are presented in Figure 2 and Table 8. Additionally, 2D schematic diagrams illustrating the interactions between C16 and the α5β1 or αvβ3 integrins are shown in Figures S6 and S7, respectively. In both integrins, C16 engages the MIDAS binding site through a combination of hydrogen bonding, hydrophobic and electrostatic interactions, and van der Waals forces.

**Figure 2:**
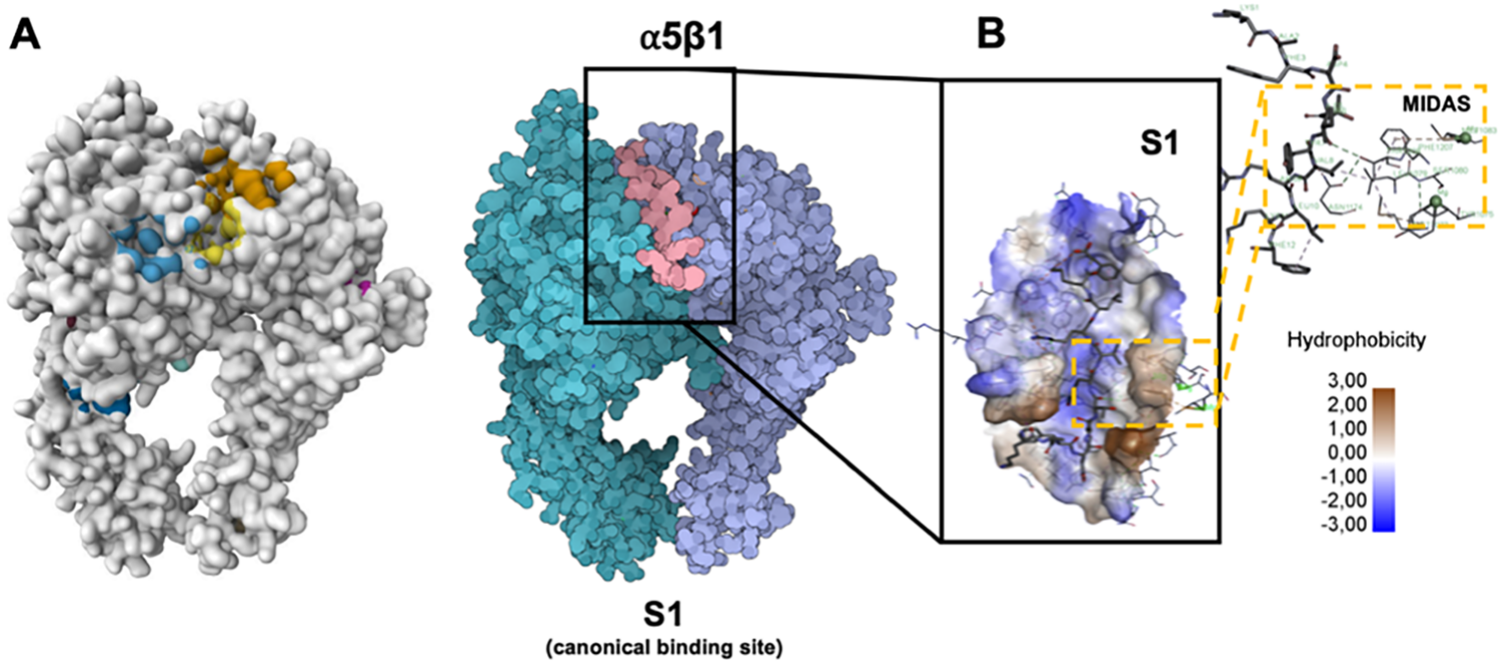
C16 peptide docking and molecular interactions with integrin α5β1. (A) Prediction of the ligand-binding sites on the α5β1 structure using PrankWeb. Best-scored pose of the C16 peptide within the MIDAS (metal ion–dependent adhesion site) pocket of integrin α5β1, visualized using SAMSON. (B) Molecular interactions of C16 with the MIDAS binding pocket generated using Discovery Studio 2020. Detailed alignment of C16 within the binding pocket, highlighting interactions with coordinated active residues in chain A: Arg220, Ala222, Tyr226, Asn256, Tyr261, Asp269, Ser277, and Asn275. Green dashed lines indicate hydrogen bonds.

**Table 8:** Docking Key Interactions between Integrin Isoforms and C16 Peptide

Integrin Isoform	Hydrogen Bonds	Bond Length (Å)	Electrostatic Interactions	Hydrophobic Chain A	Hydrophobic Chain B
**α5β1_S1**	Glu126 (A)	3.08	Glu916 (B)	Tpr157 (A)	Tyr729 (B)
	Cys783 (B)	3.20		Phe187 (A)	Ser730 (B)
	Ser823 (B)	2.70		Ile225 (A)	Lys778 (B)
				Ile225 (A)	Pro782 (B)
				Asp228 (A)	Thr784 (B)
				Leu257 (A)	Glu787 (B)
					Phe917 (B)
**αvβ3_S1**	Asp148 (A)	2.46	Asp218(A)	Asp150 (A)	Leu1079(B)
	Tyr178 (A)	2.43		Phe177 (A)	Asn1174(B)
	Thr1208 (B)	3.07		Leu209 (A)	Pro1135 (B)
				Ala215 (A)	Thr1209 (B)
				Ile216 (A)	Val1269 (B)
				Arg248 (A)	
**αvβ3_S2**	Lys42 (A)	2.50		Gly16 (A)	Asp1117 (B)
	Arg1220 (B)	2.92		Tyr18 (A)	Lys1118 (B)
				Asp83 (A)	Tyr1123 (B)
				Asp84 (A)	Ser1241 (B)
				Pro85 (A)	Ala1242 (B)
				Phe88 (A)	
				Ser90 (A)	
				Hist91 (A)	
				Arg11 (A)	
				Gln120 (A)	
				Glu121 (A)	
				Arg122 (A)	
				Glu52 (A)	
				Ser399 (A)	
				Met 400 (A)	

#### 3.3.2 Prediction of an Alternative C16 Binding Site in the αvβ3 Integrin

In contrast to the α5β1 results, the top-scoring blind docking poses of C16 in αvβ3 revealed a previously uncharacterized binding site, designated S2, located opposite the canonical S1 pocket. Additionally, a densely populated cluster was identified at the MIDAS site (S1), similar to α5β1.

Based on the interaction residues predicted in the exploratory docking, a more focused and refined docking analysis was subsequently performed on the S1 and S2 sites (Figure 3). Notably, for both integrins, the top-scoring poses coincided with the ligand-binding sites predicted by PrankWeb.

**Figure 3:**
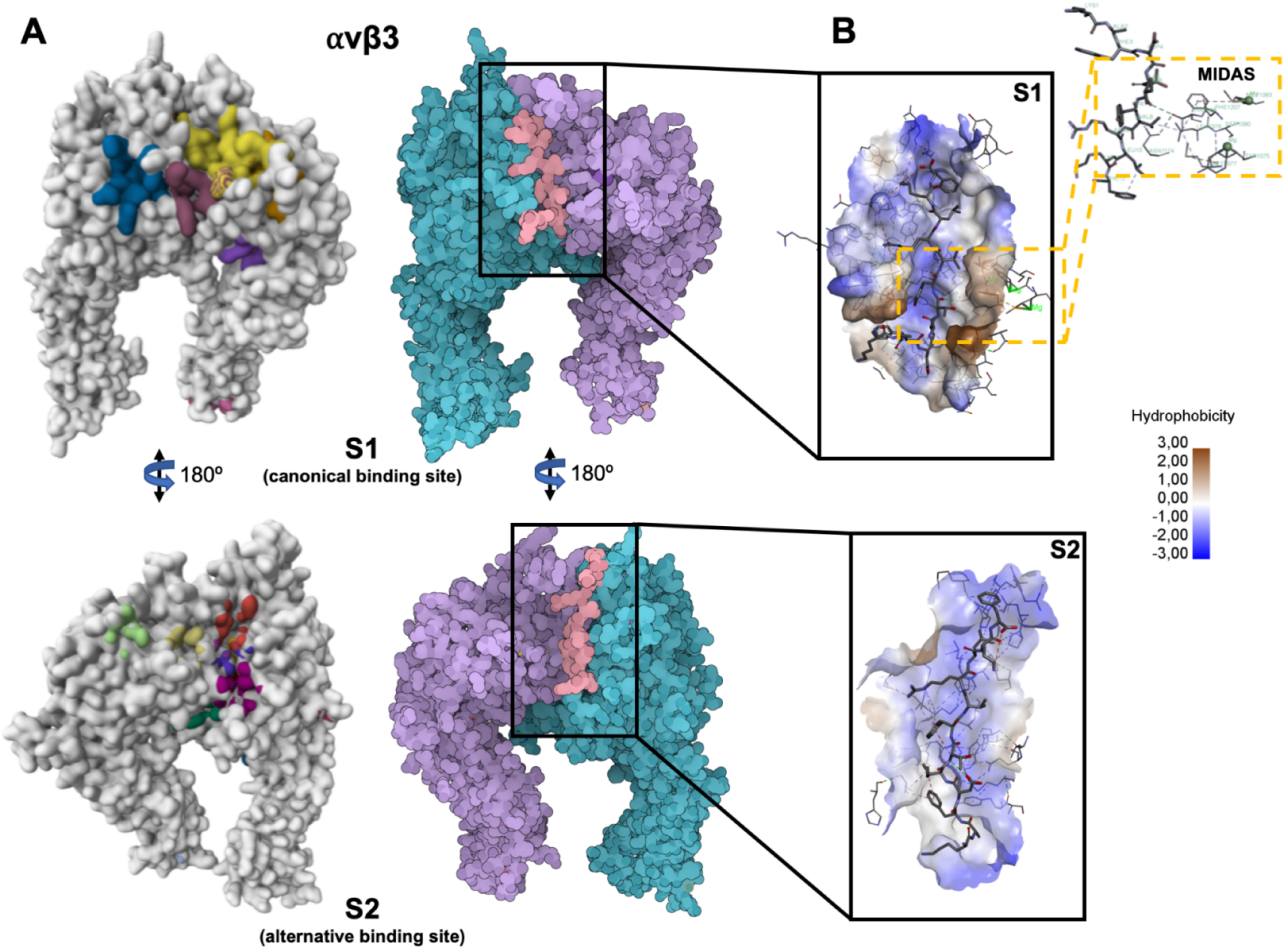
C16 peptide docking and molecular interactions with integrin αvβ3 reveal an alternative binding site. (A) On the left, prediction of potential ligand-binding sites on the integrin αvβ3 structure, showing the canonical binding Site 1 (S1, top) and the alternative binding Site 2 (S2, bottom), generated using PrankWeb. On the right, top ranked C16 pose within the hydrophobic pocket S1 (top) and S2 (bottom) of integrin αvβ3, rendered with SAMSON. (B) Molecular interaction of the C16 peptide with the hydrophobic binding pockets S1 (top) and S2 (right) of integrin αvβ3, visualized using Discovery Studio 2020. Green dashed lines represent hydrogen bonds. Inset showing the MIDAS domain.

Although the C16 peptide lacks the canonical RGD motif, its amino acid composition allows it to interact with the conserved RGD-recognition pockets (S1 sites) of both αvβ3 and α5β1 integrins. The hydrophobic residues Val, Ile, and Phe fit into the nonpolar pockets, while the basic residues Arg and Lys form electrostatic interactions with conserved acidic residues (Asp, Glu) present in both integrins. These interactions enable C16 to exploit the same conserved structural interface used by classical ECM ligands to mediate cell adhesion through both receptors. A previous study demonstrated that an RGD-mimetic ligand can induce a high-affinity conformation in integrin αIIbβ3 that enhances fibrinogen binding, suggesting that ligand mimetics such as C16 may act as partial agonists, potentially promoting integrin activation.^99^

##### 3.3.2.1 C16−α5β1 Interactions within the S1 Pocket

The interaction map (Figure S6) shows that the C16 peptide establishes a well-defined network of hydrogen bonds and hydrophobic contacts within the α5β1 S1 pocket. Three hydrogen bonds are formed with Glu126­(A), Cys783­(B), and Ser823­(B), with distances ranging from 2.7 to 3.2 Å, consistent with moderately strong polar interactions. These residues stabilize the N-terminal and central regions of the peptide, particularly the side chains of Lys1, Ile5, and Thr6.

Hydrophobic contacts involve residues from both integrin subunits, including Trp157­(A), Phe187­(A), Tyr729­(B), Pro782­(B), and Phe917­(B). These residues create a broad hydrophobic interface that accommodates Phe3, Val8, and Leu10 of C16. The pocket surface is slightly acidic due to residues such as Glu787­(B), Glu916­(B), and Asp228­(A), which interact electrostatically with the basic side chains of Lys1 and Arg9.

##### 3.3.2.2 C16−αvβ3 Interactions within the S1 Pocket

C16 establishes a similar interaction profile within the αvβ3 S1 site (Figure S7), forming three hydrogen bonds involving Asp148­(A), Asp218­(A), and Thr1208­(B), with bond lengths between 2.4 and 3.1 Å. The acidic residues Asp148­(A) and Asp218­(A), located near the MIDAS region, engage in polar contacts with the C16 peptide’s basic residues Arg9 and Lys11, reinforcing peptide anchoring through electrostatic complementarity.

Comparable to α5β1, the αvβ3 S1 pocket exhibits several hydrophobic interactions involving Phe177­(A), Leu209­(A), Ala215­(A), and Ile216­(A) of the αv chain and Leu1079­(B), Pro1135­(B), and Thr1209­(B) of the β3 chain. These residues interact with C16 Phe3, Ile5, Val8, and Leu10, forming a compact hydrophobic environment that complements the peptide’s core.

##### 3.3.2.3 Comparative Analysis of C16 Binding in α5β1 and αvβ3 S1 Sites

Importantly, the MIDAS Mg^2+^ and ADMIDAS Mg^2+^ ions in αvβ3, as well as the Ca^2+^ ion in α5β1, are coordinated by residues that also interact with the peptide backbone (Figures 2B and 3B), highlighting the contribution of these metal centers to ligand recognition.^100^ These interactions stabilize the integrin–ligand complex and may facilitate conformational adjustments required for receptor activation.

Both integrins exhibit a similar number of hydrogen bonds, polar contacts, and hydrophobic interactions with C16 within the S1 pocket, suggesting that the peptide exploits a conserved binding mechanism across isoforms. In both cases, acidic residues near the MIDAS coordinate interactions with the basic residues of C16, while neighboring hydrophobic side chains provide shape complementarity and van der Waals stabilization.

##### 3.3.2.4 C16−αvβ3 Interactions within the S2 Pocket

The αvβ3_S2 pocket displays a distinct topography and interaction pattern. It maintains a comparable number of hydrogen bonds but significantly expands the hydrophobic contact network, involving more than 12 residues distributed across both integrin chains.

The C16 residues Phe3, Ile5, Val8, Leu10, and Phe12 are deeply embedded within a hydrophobic groove delineated by Pro1135­(B), Thr1209­(B), Val1269­(B), and Tyr178­(A).

These contacts are complemented by electrostatic bridges between Arg1220­(B) and Asp83­(A) or Asp84­(A), forming a mixed polar–nonpolar interface that reinforces peptide retention. The alternating distribution of charged residues (Asp83, Arg1220, and Lys1118) interspersed with hydrophobic side chains generates an amphiphilic environment particularly well-suited for C16 binding.

#### 3.3.3 Electrostatic Repulsion Prevents C16 to Bind to the α5β1 Corresponding S2 Pocket

Topologically, the S2 cavity in αvβ3 is highly similar to the corresponding region in α5β1, suggesting that both integrins share a comparable structural framework in this area. This similarity raises the question of why C16 readily binds to the αvβ3_S2 site but not to the analogous pocket in α5β1.

To address this, we analyzed the electrostatic surface potential of both integrins and the C16 peptide. The electrostatic maps (Figure 4) revealed that the αvβ3_S2 pocket shows a distinct alternating charge distribution, with adjacent positive and negative regions that complement the electrostatic pattern of C16.

**Figure 4:**
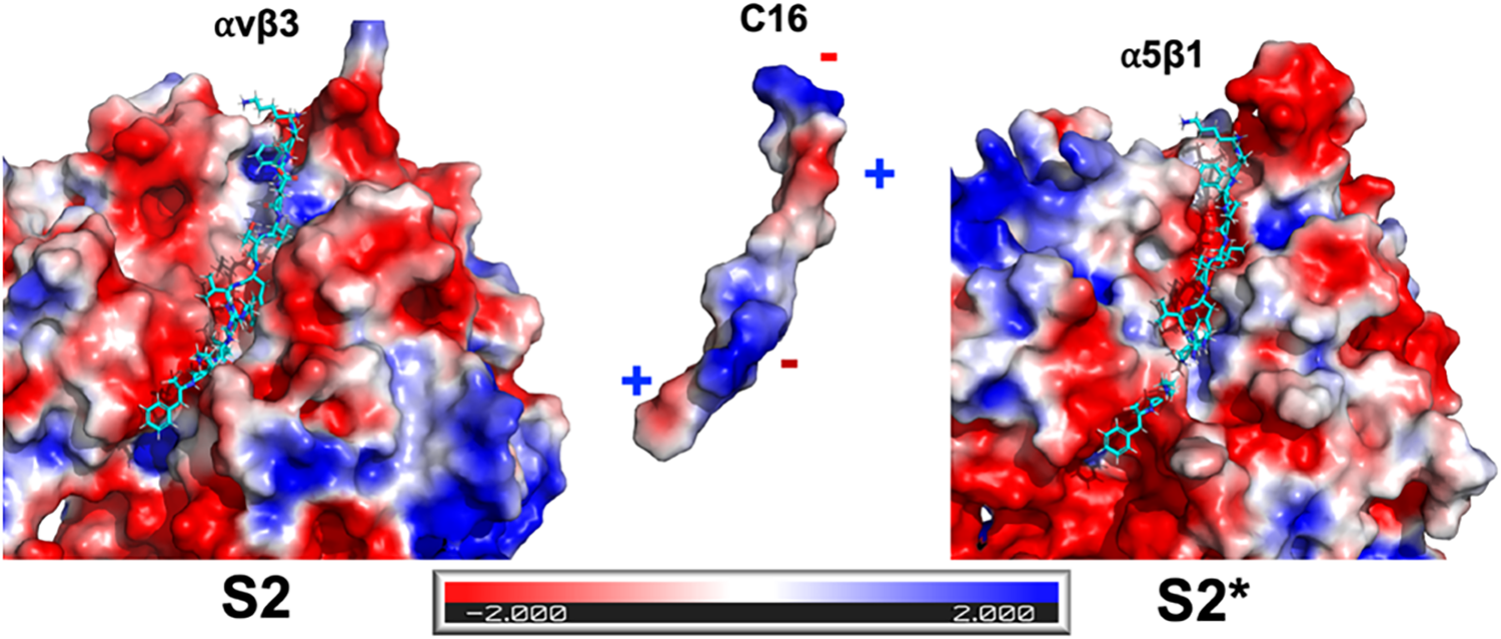
Electrostatic complementarity drives C16 binding selectivity between integrins αvβ3 and α5β1. Electrostatic potential surfaces (±2 kT/e) of the αvβ3 S2 pocket (left), the C16 peptide (center), and the corresponding α5β1 S2* pocket (right). Electrostatic potentials were calculated using the APBS Electrostatics Plugin in PyMOL. The docked C16 structure (cyan) is shown for reference.

This complementary arrangement likely facilitates the peptide’s stable binding to αvβ3 through balanced electrostatic attraction and hydrophobic accommodation.

In contrast, this pattern is not preserved in the corresponding α5β1_S2 region, which displays a predominantly negative electrostatic potential that would repel the negatively charged regions of C16, thereby preventing its stable insertion into the pocket. Thus, although both integrins share structural similarity in the S2 region, electrostatic complementarity, rather than topology alone, governs the selective recognition of C16 by αvβ3 at the S2 site.

#### 3.3.4 C16 Dual Predicted Binding Mode Suggests Differential Activation Potential of αvβ3 and α5β1 Integrins

The identification of two distinct C16 binding sites in αvβ3—one canonical (S1) and one alternative (S2)—suggests that this peptide may engage the integrin through a dual binding mechanism, potentially influencing receptor activation and downstream signaling. While both αvβ3 and α5β1 recognize C16 via the conserved S1 pocket near the MIDAS, only αvβ3 provides the additional hydrophobic S2 site that could serve as a secondary anchoring region or an allosteric interface.

The predominance of polar and electrostatic interactions in α5β1_S1 may favor transient and reversible binding, consistent with a lower binding stability. In contrast, the extensive hydrophobic network in αvβ3_S2 indicates stronger and more persistent contacts, possibly stabilizing a partially active receptor conformation.

This mechanism resembles previously described ligand-induced conformational changes in αIIbβ3 integrin,^99^ where mimetic peptides facilitated the transition toward a high-affinity state.

Altogether, these findings support the idea that C16 may act as a context-dependent integrin modulator: engaging α5β1 primarily through the classical S1 pocket, while interacting more robustly with αvβ3 through both S1 and S2 sites. This dual engagement could contribute to receptor-specific signaling outcomes and may underlie the differential roles of αvβ3 and α5β1 integrins in C16-mediated cell adhesion and migration.

To evaluate the robustness and specificity of our docking approach, we performed two additional control experiments using peptides that preserve the amino-acid composition and net charge of C16 but alter its sequence order: (i) a reversed C16 sequence and (ii) a scrambled C16 variant, in which the same residues are rearranged into a randomized order. These controls allow us to determine whether the predicted binding mode arises from sequence-specific recognition rather than generic physicochemical properties. Both control peptides were blind docked into the αvβ3 integrin using the same workflow applied to the native C16 peptide.

Despite sharing identical composition, neither control reproduced the binding geometry observed for C16 in the S2 pocket (Figure S4C). Their RMSD distributions, calculated relative to the lowest-energy C16–S2 reference pose, were broad and shifted toward substantially higher values (≈4–5 nm), indicating poor spatial convergence within the S2 pocket. In contrast, C16 docked directly into S2 exhibited a narrow, low-RMSD distribution with two distinct peaks, corresponding to two energetically favorable orientations of the peptide within the pocket. Similarly, blind docking of C16 recapitulated these same two low-RMSD peaks and additionally sampled a third peak consistent with binding at the S1 site. Together, these results demonstrate that the S2 region accommodates the native C16 sequence in a specific and reproducible manner that is not recapitulated by randomized controls, thereby supporting the sequence-dependent nature of the proposed noncanonical binding mode. These control experiments strengthen the conclusion that the predicted S2 binding mode is sequence-specific and not an artifact of overall peptide physicochemical properties.

### 3.4 Molecular Dynamics Simulations of Integrins

MD simulations can accurately predict a wide range of association phenomena, significantly enhancing our understanding of the interactions between laminin-derived peptides and integrin molecules.^101^ In this study, MD simulations were conducted to evaluate and validate *in silico* the stability of the C16–integrin complexes. Three independent 100 ns MD simulations were performed for each system. The simulations included the two unbound integrins and three complexed systems: α5β1_Apo, αvβ3_Apo, α5β1_S1, αvβ3_S1, and αvβ3_S2. We investigated the key interactions between C16 and αvβ3 integrin, and the conformational changes in the αvβ3 and α5β1 integrins upon C16 binding. The overall stability of each complex was further assessed through time-averaged structural properties and estimation of binding free energies.

#### 3.4.1 C16 Peptide Complex with α5β1

RMSD measures the average deviation of the complex’s conformation of its original conformation over time and helps to evaluate whether the complex has reached a stable state.^102,103^ The structural stability of the α5β1 integrin was evaluated by monitoring the protein RMSD and the per-residue RMSF over 100 ns of MD simulations (Figure 5).

**Figure 5:**
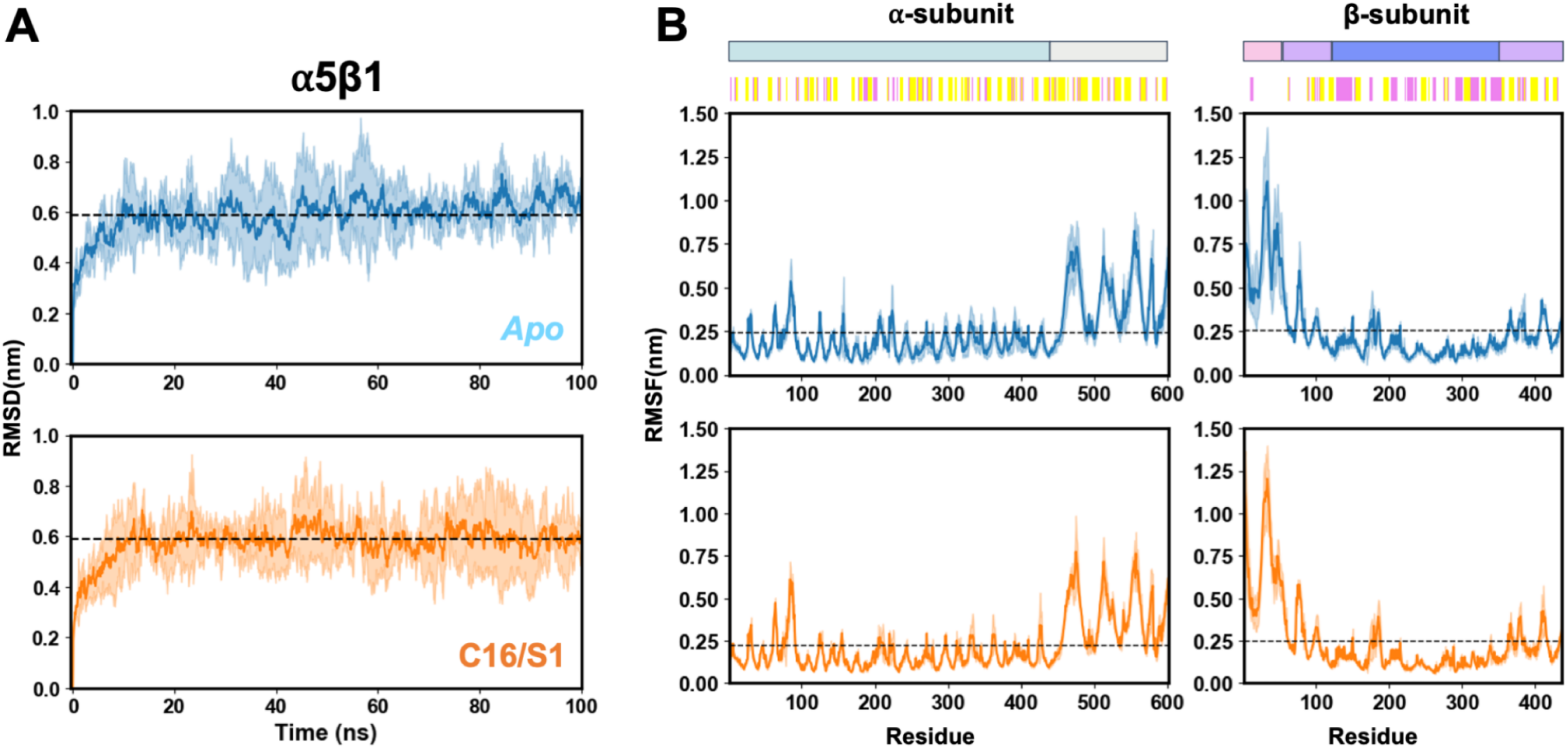
Structural stability and flexibility of the α5β1 integrin during MD simulations. (A) RMSD of the Cα atoms for the unbound (Apo, blue) and complexed (C16/S1, orange) α5β1 integrin over 100 ns of simulation. The solid lines represent the mean RMSD, and the shaded regions indicate standard deviations across three independent replicates. (B) RMSF of Cα atoms in the α- (left) and β-subunits (right) for both Apo and C16/S1 systems. Colored bars above the RMSF plots indicate the integrin structural domains (top): β-propeller (cyan), thigh (light gray), PSI (pink), hybrid (violet), and βI-like (dark blue); and the secondary structure elements (bottom): alpha-helices (magenta) and beta sheets (yellow).

Both the unbound (Apo) and the C16-bound (C16/S1) systems reached equilibrium after approximately 20 ns, exhibiting average RMSD values around 0.5–0.6 nm, indicating stable trajectories throughout the simulations. The overall similarity in RMSD profiles suggests that C16 binding does not induce major conformational rearrangements in the α5β1 integrin structure.

Analysis of the RMSF profiles for the α- and β-subunits revealed that most regions of the integrin remained relatively rigid, with limited fluctuations below 0.3 nm. However, local increases in flexibility were observed in specific loop segments and terminal regions, which are typically associated with surface exposure and ligand recognition. Notably, C16 binding slightly reduced fluctuations in some extracellular loops, suggesting that peptide association may stabilize these regions and contribute to a more compact receptor conformation.

The global structural compactness of the α5β1 integrin was evaluated by analyzing the radius of gyration (Rg) and its correlation with RMSD over the 100 ns simulations (Figure S8). Both the Apo and C16/S1 systems exhibited narrow distributions of Rg and RMSD, indicating that peptide binding does not lead to substantial changes in the overall compactness or folding of the integrin. The average Rg values remained stable around 3.6–3.7 nm, confirming that both systems reached equilibrium and maintained a compact structural organization throughout the simulations.

A positive linear correlation between Rg and RMSD (*R* ≈ 0.6–0.7) was observed for both systems, suggesting that minor conformational adjustments occur in a coordinated manner without significant expansion of the protein structure. Interestingly, the C16-bound integrin showed slightly reduced fluctuations and a marginally higher mean Rg value, suggesting that C16 binding contributes to a subtle stabilization of the extracellular region while preserving the global fold. These findings are consistent with the RMSD and RMSF analyses (Figure 5), further supporting that C16 association stabilizes local regions of α5β1 without inducing large-scale conformational rearrangements.

The solvent-accessible surface area (SASA) was examined to explore how C16 binding affects the solvent exposure and compactness of α5β1 during the simulations (Figure S9). Both the Apo and C16/S1 systems displayed narrow and overlapping SASA distributions, with mean values around 470 nm^2^, suggesting that peptide binding does not cause significant alterations in the overall solvent-exposed surface of the integrin. The SASA remained stable throughout the 100 ns trajectories, indicating well-equilibrated systems, and confirming the compact structural behavior observed in the Rg analysis (Figure S8). However, correlation analyses between SASA, RMSD, and Rg revealed there are no linear relationships (*R* = 0.15–0.32), indicating that variations in solvent exposure are largely decoupled from global conformational deviations. This suggests that local rearrangements, particularly those in surface-exposed loops, may contribute more strongly to SASA fluctuations than large-scale structural motions. The slightly reduced SASA fluctuations in the C16-bound system further indicate that C16 association may stabilize certain flexible regions without substantially modifying the integrin’s overall conformation.

To investigate the conformational space sampled by α5β1 during the simulations, a clustering analysis was performed based on protein RMSD (Figure S10). Both the Apo and C16/S1 systems displayed a rapid decay in the number of structures per cluster, indicating that a limited number of conformations dominate the ensemble. The cumulative distribution revealed that approximately 80% of the total sampled conformations are represented by the first few clusters, demonstrating that the integrin dynamics are restricted to a relatively compact conformational space over the 100 ns trajectories. The superposition of representative cluster centroids confirmed that the global fold of α5β1 is largely preserved in both systems. The C16-bound state exhibited slightly reduced structural dispersion compared to the Apo form, consistent with the modest stabilization inferred from the RMSD, Rg, and SASA analyses. These results suggest that C16 binding constrains local flexibility and limits the exploration of alternative conformational states, supporting a stabilizing effect on the integrin extracellular domain without inducing major structural rearrangements.

The temporal distribution of cluster IDs was analyzed to examine the persistence and recurrence of conformational states during the simulations (Figure S11). Both Apo and C16/S1 trajectories exhibited transitions between a limited number of dominant clusters, consistent with the restricted conformational space observed in the overall clustering analysis (Figure S10). The concatenation of the three replicates enabled a direct comparison of shared structural states, with the same cluster centroids representing equivalent conformations across simulations.

In the Apo system, cluster transitions were more frequent, reflecting higher conformational mobility and a broader exploration of accessible states. In contrast, the C16-bound system displayed longer residence times within specific clusters and fewer transitions, indicating increased structural stability and reduced conformational flexibility upon peptide binding. These results reinforce the conclusion that C16 association stabilizes α5β1 by constraining its conformational dynamics while maintaining the overall native architecture of the integrin.

#### 3.4.2 C16 Peptide Complex with αvβ3

To evaluate the structural stability of αvβ3 and the effect of C16 binding, the RMSD and RMSF profiles were analyzed over 100 ns of MD simulations (Figure 6). The apo αvβ3 system remained stable with an average RMSD of approximately 0.4 nm, indicating limited structural deviation from the initial conformation. In contrast, the C16/S1 complex displayed a gradual RMSD increase up to ∼0.7 nm, suggesting moderate conformational rearrangements upon peptide binding, whereas the C16/S2 complex maintained RMSD values around 0.45 nm, comparable to the apo state, reflecting higher stability.

**Figure 6:**
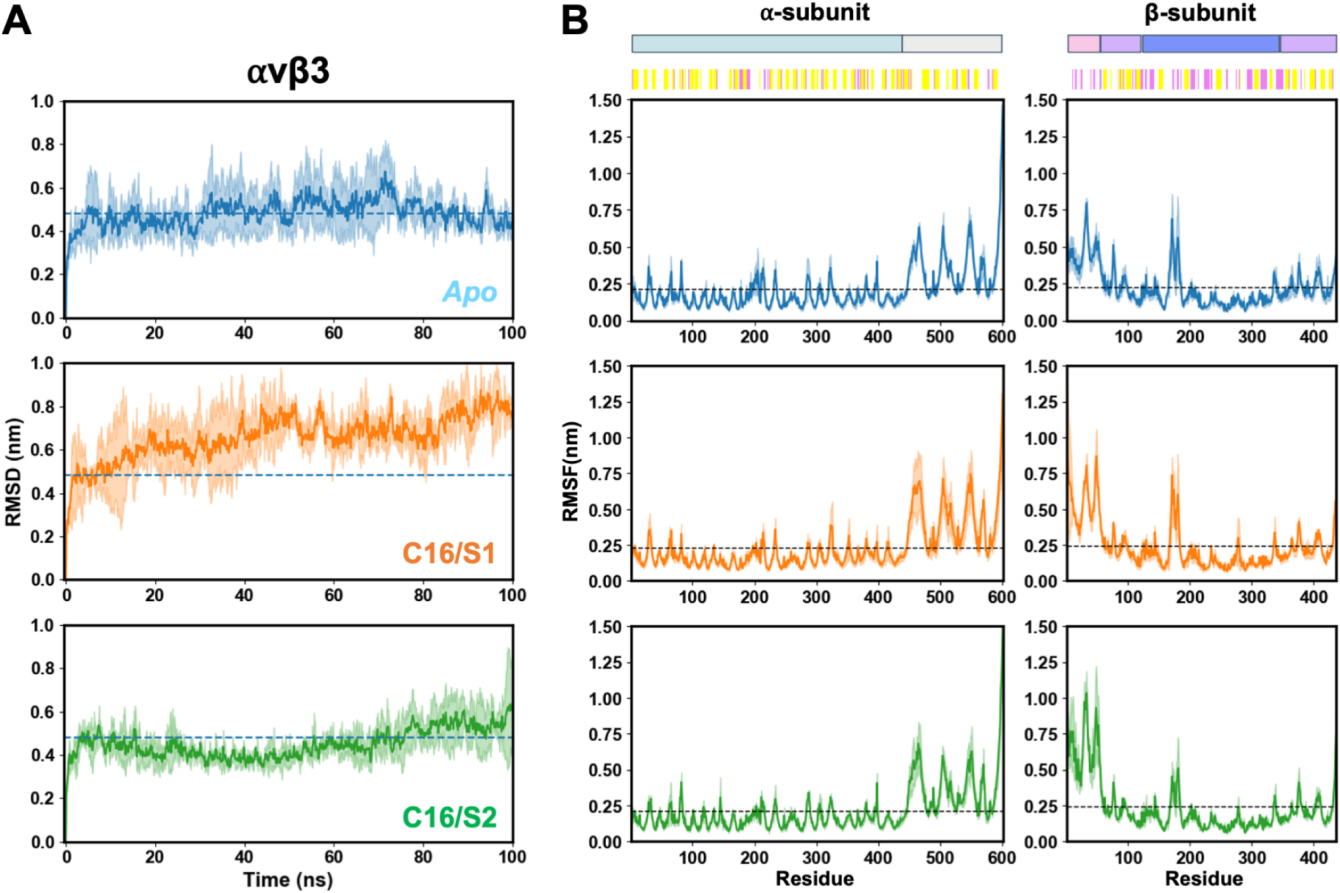
Structural stability and flexibility of the αvβ3 integrin during MD simulations. (A) RMSD of the Cα atoms of αvβ3 in the apo form (blue) and in complex with the C16 peptide at binding sites S1 (orange) and S2 (green). The solid lines represent the average values, and the shaded areas represent the standard deviations. (B) Per-residue RMSF for the α- (left) and β- (right) subunits in the same systems. Secondary structure elements are depicted above the plots. The dashed lines indicate the mean RMSF value for each system. Colored bars above the RMSF plots indicate the integrin structural domains (top) and the secondary structure elements (bottom).

Residue-based RMSF analysis (Figure 6B) showed similar flexibility patterns across systems, with prominent peaks corresponding to loop regions within the β-propeller and hybrid domains of the α- and β-subunits. Increased local fluctuations were detected in the αvβ3–C16/S1 complex, particularly near the ligand-binding interface, while the C16/S2 complex exhibited dampened fluctuations in these regions, consistent with a more stable peptide–integrin interaction.

When compared with α5β1, αvβ3 exhibited overall lower RMSD and RMSF amplitudes, suggesting a more rigid global architecture. The α5β1–C16 complexes underwent more pronounced conformational changes, especially in the S1 binding mode, where larger deviations were observed in the β-subunit.

The Rg analysis was performed to assess the global compactness and conformational stability of αvβ3 integrin during the 100 ns MD simulations (Figure S12). The distributions show that all systems maintained relatively narrow Rg and RMSD ranges, reflecting equilibrated and structurally stable trajectories. The apo and C16/S2 systems exhibited nearly overlapping Rg distributions centered around ∼3.5 nm, indicating similar global compactness, while the C16/S1 system showed a modest shift toward higher Rg values (∼3.6 nm) and a broader RMSD distribution, suggesting slightly increased structural flexibility.

The time-dependent Rg profiles confirm that all systems remained stable after ∼20 ns of equilibration, with no indication of unfolding or large-scale expansion. Nevertheless, small oscillations were observed in the C16/S1 complex, consistent with transient rearrangements within the extracellular domain that were not apparent in either the apo or C16/S2 trajectories. The correlation analysis revealed a moderate positive relationship between Rg and RMSD (*R* ≈ 0.45–0.55), indicating that conformational deviations are accompanied by small but coordinated changes in global compactness.

To explore whether peptide binding affects the molecular surface exposure of αvβ3 integrin, we evaluated the SASA throughout the 100 ns simulations (Figure S13). The distributions reveal distinct SASA profiles for each system. The apo form displays a narrow distribution centered near 470 nm^2^, indicating a compact and stable conformation. Upon C16 binding, both S1 and S2 complexes exhibit broader and slightly shifted distributions, suggesting enhanced structural breathing and transient solvent exposure. The RMSD distributions confirm that all systems reached stable equilibria, with no major unfolding events. The time-dependent SASA profiles show that the apo integrin remains stable during the entire trajectory, whereas the C16/S1 complex exhibits modest fluctuations toward higher SASA values over time, consistent with local rearrangements near the peptide-binding region. In contrast, C16/S2 shows a more stable evolution, maintaining values close to the apo state, indicating a less perturbative interaction.

The correlation plots in Figure S13C show no linear relationship between SASA and RMSD (R= 0.22–0.38), as well as between SASA and Rg (*R* = 0.25–0.37). These low correlations suggest that while peptide binding induces local flexibility and slight expansion of the solvent-exposed surface, the overall tertiary structure remains largely conserved. Among the systems, C16/S1 displays the strongest correlations, reinforcing that this binding mode elicits a subtle increase in conformational plasticity.

When compared to α5β1 integrin (Figure S9), αvβ3 exhibits narrower SASA fluctuations and lower SASA–RMSD correlation coefficients (typically ≤ 0.35 vs 0.5 for α5β1), indicating a more rigid and compact architecture.

To characterize the conformational diversity of αvβ3 integrin and assess the effect of C16 peptide binding, the concatenated MD trajectories of each system were subjected to clustering analysis based on protein RMSD (Figure S14). The distributions show that all systems are dominated by a small number of highly populated clusters, followed by a steep decay in frequency. The apo integrin exhibits a more heterogeneous cluster distribution, with approximately 8–10 clusters accounting for 80% of the sampled conformations. In contrast, the C16-bound systems, particularly C16/S2, display fewer and more dominant clusters, suggesting a stabilization of specific conformational states upon peptide binding. The cumulative cluster percentage curves further support these observations. For the apo system, the 80% threshold is reached near cluster ID 9, whereas for C16/S1 and C16/S2, this occurs around clusters 5 and 3, respectively. This trend indicates a reduction in conformational entropy upon complex formation, reflecting enhanced structural stabilization of the integrin when bound to C16.

Notably, the C16/S1 complex maintains a moderate level of conformational variability, consistent with its higher RMSD and broader radius of gyration distribution (Figures 6 and S12), suggesting that binding at S1 allows partial breathing motions of the headpiece. Conversely, C16/S2 exhibits the narrowest distribution, indicative of a more compact and stable state.

When compared with α5β1 (Figure S10), αvβ3 displays overall fewer populated clusters and a faster saturation of the cumulative percentage, underscoring its intrinsically lower conformational flexibility. Together, these data indicate that while both integrins undergo conformational stabilization upon C16 binding, αvβ3 shows a more pronounced restriction of structural diversity, especially when the peptide interacts at the S2 site.

Figure S15 displays the temporal evolution of the cluster IDs obtained from the αvβ3 trajectories in the Apo, C16/S1, and C16/S2 systems. These plots illustrate the conformational substates visited during the simulations and how the presence of the C16 peptide influences the structural dynamics of the integrin. In the Apo system, αvβ3 exhibits a highly dynamic behavior, with frequent transitions between clusters throughout the trajectory. The scattered distribution of cluster IDs indicates that the system explores a broad range of conformational substates without long-term stabilization in any particular state.

Upon C16 binding at site S1, the conformational sampling becomes more restricted. Although αvβ3 still transitions intermittently among clusters, the occupancy is now concentrated within a smaller subset of low-index clusters, indicating partial stabilization of the integrin structure. The reduced diversity of sampled states suggests that C16 engagement at S1 induces local ordering of the ligand-binding region, while allowing the integrin to retain a degree of flexibility necessary for its functional plasticity.

For the C16/S2 system, the distribution of cluster IDs becomes even more confined, with the trajectory dominated by a few persistent clusters. This behavior indicates that binding at the alternative S2 site promotes greater stabilization of the integrin conformation compared to S1. However, occasional short-lived transitions still occur, consistent with the dynamic coordination of residues and ions around the ligand-binding pocket that maintain structural adaptability.

### 3.5 Dominant Integrin Motions during the MD Simulations

PCA was performed on a covariance matrix derived from the concatenated trajectory containing the triplicates of all systems to enable direct comparison of their dominant motions within a common conformational subspace. This approach allows the identification of shared collective modes and the evaluation of how peptide binding redistributes the conformational sampling along identical PCs, rather than comparing unrelated eigenvectors from separate analyses.

The analysis of the eigenvectors (Figure 7) revealed that PC1 describes a global bending mode involving the Thigh and Psi domains, corresponding to the interdomain closing motion that regulates integrin headpiece opening. PC2, in turn, represents twisting and lateral rearrangements between the α- and β-subunits.

**Figure 7:**
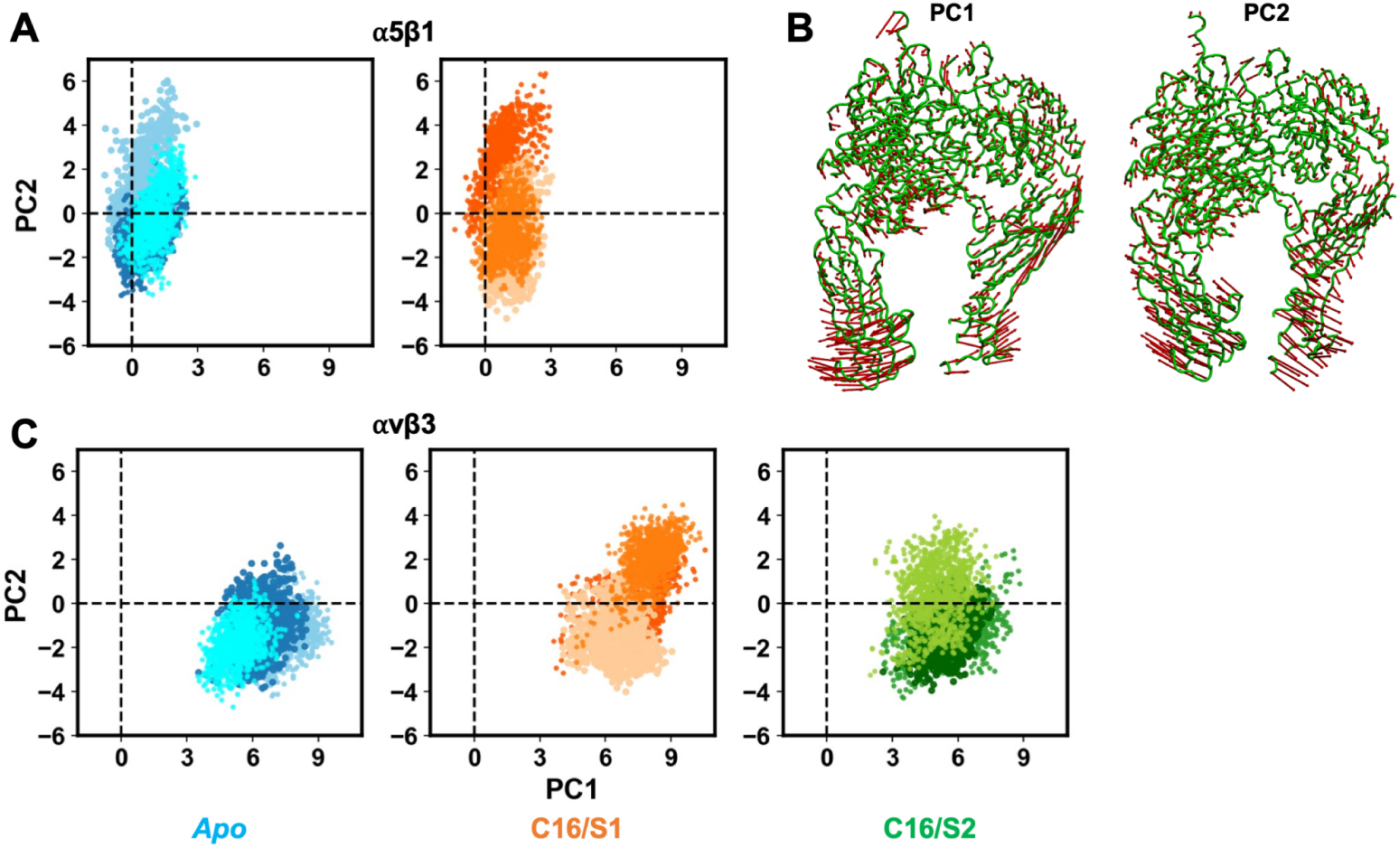
PCA of the concatenated molecular dynamics trajectories, including triplicates, for both α5β1 and αvβ3 integrins. (A) Projections of each structure onto the PC1 versus PC2 subspace for the α5β1 systems. (B) Directional vectors of the PC1 and PC2 mapped onto the integrin backbone, illustrating the dominant collective motions. (C) Projections of each structure onto the PC1 versus PC2 subspace for the αvβ3 systems. Apo, C16/S1, and C16/S2 ensembles are shown in shades of blue, orange, and green, respectively.

Interestingly, the α5β1 ensemble projections on the first PC remain close to zero, whereas αvβ3 explores higher positive values (greater than 3), indicating that αvβ3 samples more pronounced bending or closing motions.

In contrast, for αvβ3, the apo and C16-bound ensembles exhibited partially overlapping projections, with C16/S1 showing a modest shift along PC1 and C16/S2 being more compact, suggesting that C16 binding exerts a smaller perturbation on αvβ3 dynamics compared to α5β1.

Altogether, the PCA results indicate that C16 binding differentially modulates the conformational landscapes of α5β1 and αvβ3, reflecting distinct allosteric coupling and dynamic plasticity mechanisms between these integrin subtypes.

### 3.6 Normal Modes Predict Conformational Changes between αvβ3 and α5β1

NMA was employed to investigate the collective motions underlying the conformational transition between αvβ3 and α5β1 integrins. The overlap analysis against the deformation vector (Figure 8) revealed that only a few low-frequency modes, particularly the first five, account for the majority of the structural deformation between both integrins.

**Figure 8:**
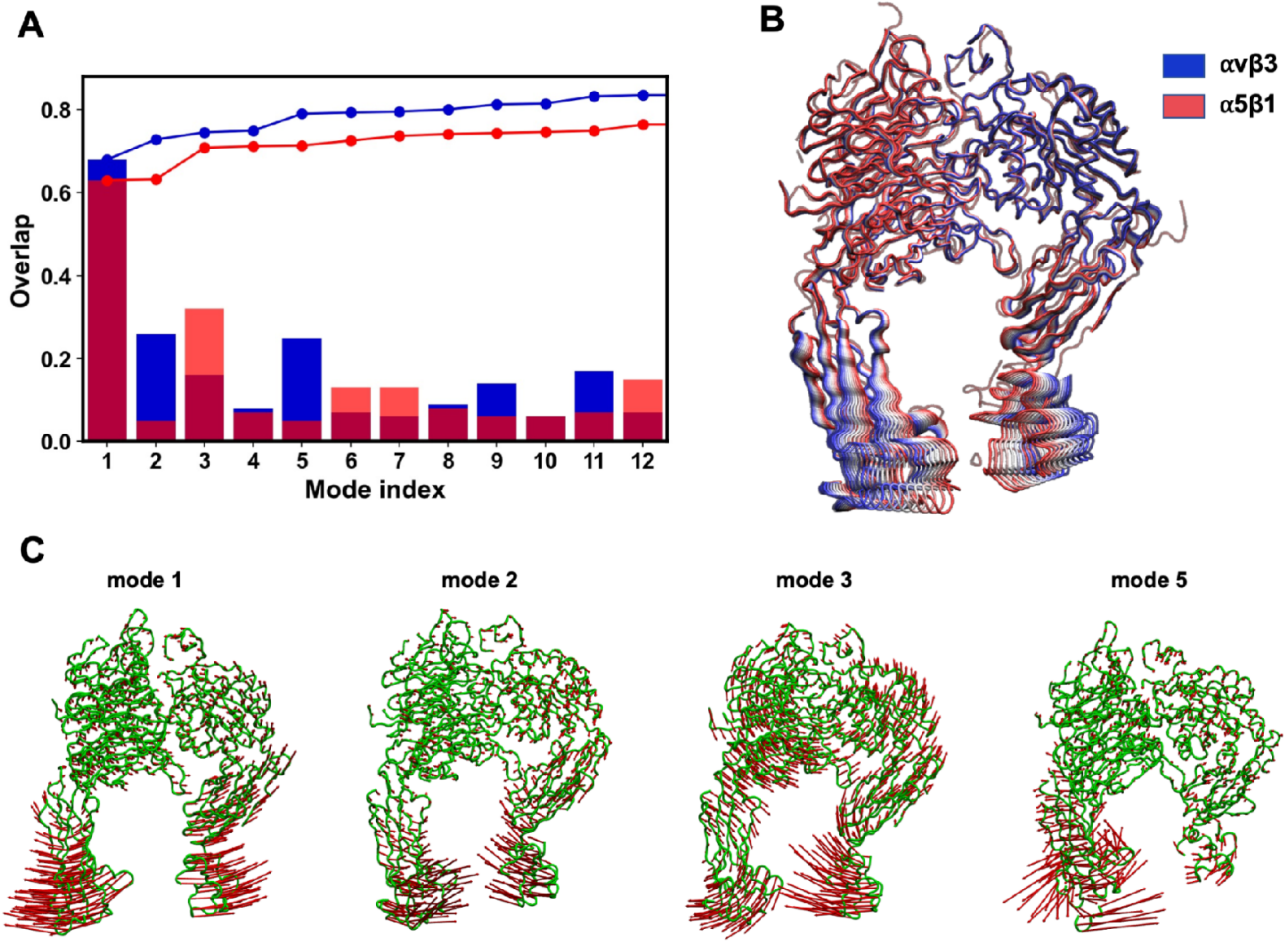
Normal mode overlap and collective motions describing the conformational transition between αvβ3 and α5β1 integrins. (A) Overlap between the low-frequency normal modes of αvβ3 (blue) and α5β1 (red) and the deformation vector corresponding to the transition between the two integrin structures. Bars indicate the contribution of each individual mode, and lines represent the cumulative overlap. (B) Morphing trajectory of the αvβ3 integrin structure displaced along its first four low-frequency modes, which collectively reproduce the transition toward the α5β1 conformation. (C) Visualization of the direction and amplitude of motion for representative normal modes (modes 1, 2, 3, and 5).

The cumulative overlap rapidly converges above 0.7, demonstrating that these modes capture the essential large-scale rearrangements associated with the transition. This result highlights that the intrinsic flexibility encoded in the global modes of the integrin structures is sufficient to describe the major functional conformational changes observed experimentally.

The morphing trajectory derived from these dominant modes (Figure 8B) illustrates a continuous deformation pathway that transforms the αvβ3 structure toward the α5β1-like conformation. The transition mainly involves a bending motion between the headpiece and the lower domains, consistent with the integrin “switchblade-like” opening mechanism. This intrinsic coupling between the leg and head domains suggests that both αvβ3 and α5β1 share a conserved mechanical core, although the amplitude and directionality of the motions differ subtly, reflecting their distinct functional dynamics and ligand preferences.

The visualization of individual normal modes shows that modes 1 and 2 primarily drive a hinge-like bending centered at the interface between the Thigh and PSI domains, while modes 3 and 5 describe additional twisting and shear movements of the headpiece. These collective displacements correspond to the conformational breathing motions that facilitate integrin activation and ligand binding.

Figure S16 provides a quantitative comparison between the essential dynamics extracted from the MD trajectories and the intrinsic normal modes derived from the αvβ3 integrin structure. The variance plots show that a limited number of collective motions dominate the global dynamics in both representations, with the first five PCs or normal modes accounting for more than 80% of the total variance. This indicates that both MD and NMA capture highly correlated large-scale motions that underlie the global flexibility of the integrin ectodomain, suggesting that the intrinsic low-frequency modes encode the main directions of motion accessible during the simulations.

The overlap analysis shows that the first two PCs have the highest correspondence with modes 1 and 2, reaching overlap values above 0.8. This strong correlation demonstrates that the low-frequency normal modes effectively reproduce the collective bending and twisting movements captured by MD. The RMSIP results confirm the consistency between the MD-derived and NMA-derived subspaces, with cumulative values approaching 0.8 when the first five normal modes are included. This high level of similarity supports the idea that the low-frequency normal modes efficiently represent the functional flexibility of the integrin and that the conformational transition between αvβ3 and α5β1 integrins occurs along intrinsic dynamic pathways encoded in their elastic network structures.

Figure S17 provides insight into how the conformational sampling obtained from the MD trajectories maps onto the intrinsic collective motions predicted by normal-mode analysis (NMA). The projections of α5β1 and αvβ3 integrin trajectories onto the first two low-frequency modes highlight the relationship between the spontaneous fluctuations sampled in MD and the structural directions encoded in the elastic network model. For the α5β1 integrin, the three replicates display compact and nearly symmetric distributions, suggesting a relatively confined conformational ensemble dominated by small-amplitude fluctuations around a more closed conformation, consistent with the PC projections in Figure 7, where this integrin exhibited limited excursions along PC1. The tight clustering of trajectories also implies a stable network of interdomain contacts and restricted hinge-like motion between the Thigh and PSI domains.

In contrast, the αvβ3 integrin shows broader and asymmetric distributions along mode 1, particularly in the C16-bound states. This indicates an enhanced exploration of the conformational landscape along the bending axis that defines the transition between bent and extended states. Together, these results reinforce that the low-frequency normal modes capture the dominant collective deformations that underlie integrin plasticity, and that αvβ3 exhibits higher dynamic adaptability upon peptide binding than α5β1.

### 3.7 C16 Flexibility and Binding Free Energy Estimation

#### 3.7.1 C16 Dynamics in the Integrin Binding Sites

Figure 9 highlights the conformational flexibility of the C16 peptide when bound to α5β1 and αvβ3 integrins. The RMSD and RMSF analyses provide complementary views of how the peptide adapts to each integrin’s binding interface.

**Figure 9:**
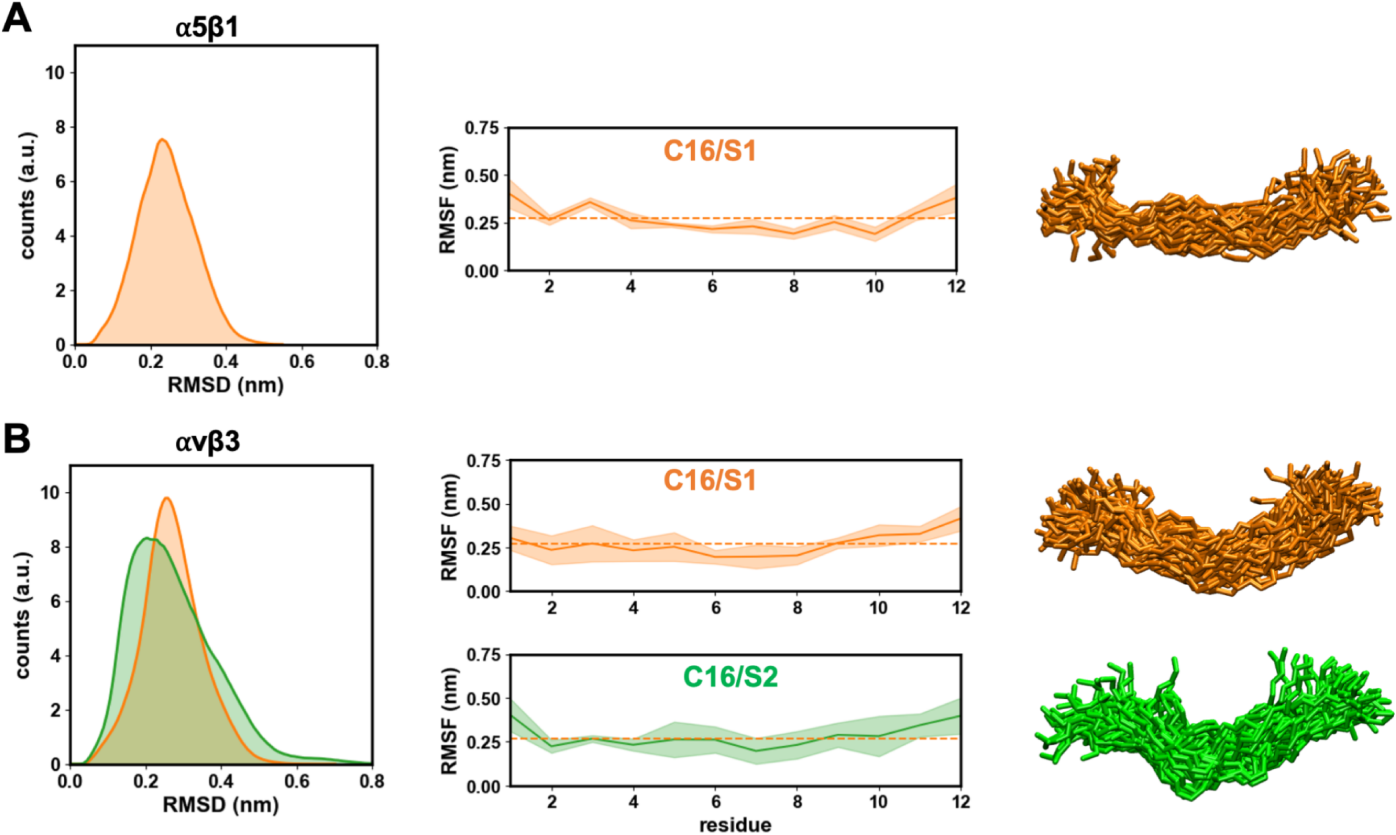
Flexibility of the C16 peptide when complexed with integrins. (A) Pairwise RMSD distribution of C16 atoms (left), RMSF per residue (middle), and superposition of representative C16 conformations (right) in complex with α5β1 integrin. (B) Equivalent analysis for the αvβ3 integrin. RMSD and RMSF values represent the average over three independent simulations, with shaded areas indicating the standard deviation among replicates.

In complex with α5β1 (Figure 9A), the C16 peptide exhibits a narrow RMSD distribution centered around ∼0.25 nm, suggesting that the bound conformation remains relatively stable throughout the simulation. The RMSF profile confirms this, showing minimal per-residue fluctuations with values below 0.3 nm across most residues. This rigidity indicates that the C16 peptide adopts a well-defined orientation when interacting with α5β1, stabilized by consistent hydrogen bonds and hydrophobic interactions at the binding site.

In contrast, the αvβ3-bound peptide shows broader RMSD distributions and slightly higher fluctuations, particularly in the C16/S2 complex. The RMSD histogram for C16/S1 peaks near 0.3 nm, while that for C16/S2 is shifted toward lower values, suggesting that the alternative binding pose (S2) stabilizes a distinct but more flexible peptide arrangement.

RMSF analysis reveals modest fluctuations, mainly in terminal residues, consistent with peripheral flexibility while maintaining a stable core interaction region. This dynamic adaptation may reflect the larger conformational space accessible at the αvβ3 interface, which accommodates peptide movement while preserving critical contacts at the MIDAS and adjacent regions.

Overall, these results suggest that C16 binds more rigidly to α5β1, consistent with a highly complementary interface, whereas its interaction with αvβ3 is more plastic, allowing subtle rearrangements of the peptide backbone. Such conformational adaptability might be functionally relevant, enabling C16 to interact with both integrins but with distinct stability and orientation characteristics that could influence downstream signaling or adhesion strength.

#### 3.7.2 Hydrogen Bonding Analysis

Figure S18 presents the time evolution of hydrogen bonds formed between the C16 peptide and the integrins during the simulations, serving as a measure of interfacial stability and binding persistence. In α5β1, the number of hydrogen bonds remains relatively constant throughout the trajectory, fluctuating around an average of 6–8 bonds. This stability supports the RMSD and RMSF results, confirming that the C16/α5β1 complex maintains a well-defined binding geometry with limited conformational drift.

The small amplitude of fluctuations indicates that the C16 peptide is tightly anchored to the integrin surface, consistent with a strong and specific recognition at the MIDAS region. In contrast, the αvβ3 complexes exhibit greater variability in the number of hydrogen bonds, reflecting a more dynamic peptide–protein interface. The C16/S1 complex maintains an average of ∼6 hydrogen bonds, while the C16/S2 complex shows a slightly lower and more fluctuating pattern (4–6 bonds). This higher variability suggests that the S2 binding mode allows the peptide to transiently rearrange its hydrogen-bonding pattern, consistent with its enhanced conformational flexibility observed in RMSD and RMSF analyses (Figure 9B). Such transient hydrogen-bond exchanges likely contribute to the adaptability of C16 in accommodating the structural plasticity of αvβ3.

The hydrogen bond occupancy analysis provided in Table S2 reveals that several peptide–integrin interactions are stably maintained throughout the MD simulations, confirming and extending the docking results.

In the α5β1_S1 system, the peptide established recurrent hydrogen bonds with residues located near the MIDAS region, consistent with the docking predictions that highlighted this site as a key anchoring interface. These hydrogen bonds persisted for more than 10% of the simulation time across all replicates, suggesting a robust interaction network that stabilizes the C16 peptide in the canonical binding groove.

In the αvβ3_S1 complex, the peptide displayed a similar hydrogen-bonding pattern, involving residues from both the α and β subunits that flank the MIDAS and ADMIDAS regions. Several of the hydrogen bonds identified in the docking pose were retained during the dynamics, indicating that the peptide remains well accommodated at this canonical binding site. However, the MD trajectories revealed fluctuations in occupancy among different replicates, reflecting the inherent flexibility of the binding pocket and the dynamic rearrangement of side chains and coordinated water molecules that modulate peptide stabilization. Interestingly, the αvβ3_S2 system exhibited a distinct hydrogen-bonding profile, with fewer but more persistent interactions compared to S1. Some residues forming transient hydrogen bonds in S1 were replaced by new stabilizing contacts in S2, suggesting that the alternative binding site may favor a more specific but less extensive hydrogen-bonding network. This behavior aligns with the docking results, where the S2 pose involved fewer but geometrically optimized hydrogen bonds. Overall, these findings indicate that while the docking results captured the key anchoring interactions, MD simulations provide a more comprehensive view of hydrogen bond stability and reveal subtle differences in binding persistence and adaptability between α5β1 and αvβ3 integrins.

The hydrogen bond occupancy analysis revealed that each C16–integrin complex established a distinct and stable network of interactions during the molecular dynamics (MD) simulations. For the α5β1–C16/S1 complex, the most persistent hydrogen bonds involved the LEU10_N and LEU10_HG of C16 with the GLU916_O from the integrin, with occupancies exceeding 80% in some replicas. These contacts indicate that the peptide’s C-terminal region remains stably anchored to the receptor surface. Such high-occupancy bonds suggest the peptide is well accommodated within the MIDAS-adjacent groove of α5β1, consistent with docking results that predicted GLU916 as a key residue forming polar contacts with C16.

In contrast, the αvβ3–C16/S1 complex displayed high hydrogen bond occupancies between SER123_N and SER121_OG of the integrin and ASP4_OD1 from C16, mainly during the third replicate, run3, (>90%). These interactions indicate that the peptide forms stable polar contacts through its N-terminal acidic residues, reinforcing the strong anchoring observed in the docking simulations at the MIDAS site. Interestingly, the dynamic behavior suggests that the stability of these hydrogen bonds is replica-dependent, pointing to possible alternative peptide orientations within the same binding pocket. The overall pattern aligns with docking predictions showing multiple hydrogen bonds between the peptide’s ASP and GLU residues and the integrin’s coordinating loops.

For the αvβ3–C16/S2 complex, the most persistent hydrogen bonds occurred between TYR7_OH of C16 and ALA263_O of the integrin, as well as between TRP93_NE1 and TYR7_OH, forming an internal hydrogen bond network that stabilizes the peptide conformation. This differs from the S1 binding mode, where hydrogen bonding primarily involves interfacial contacts. The S2 interactions indicate that C16 maintains local stability through site-specific hydrogen bonds and hydrophobic contacts. ALA263 contributes to peptide stabilization mainly via backbone and van der Waals interactions. Nearby aromatic residues, such as TYR7 (from C16) and TRP93, further enhance binding through π–π and hydrogen bonding interactions. Together, these contacts define a compact and energetically favorable binding mode at the αvβ3 S2 site, supporting the existence of an alternative and stable conformation distinct from the canonical MIDAS (S1) interaction.

#### 3.7.3 C16 Gets Structured upon Integrin Interaction

The C16 peptide starts in a random coil conformation and progressively adopts β-strand structures during the simulations in all systems (Figure S19). In α5β1 complexes, β-strands form transiently, indicating a flexible interaction and gradual stabilization at the interface. In contrast, in αvβ3 complexes, β-strand formation occurs earlier and remains more stable, suggesting that this binding environment promotes a more ordered C16 conformation. These results indicate that β-structuring of C16 contributes to complex stabilization, with distinct dynamic behavior depending on the integrin subtype.

Overall, both integrins retained their characteristic β-rich fold, though local fluctuations were evident, reflecting the inherent flexibility of loop regions and interdomain linkers in response to peptide binding and solvent dynamics.

#### 3.7.4 Binding Free Energy Estimation of C16/Integrin Complexes

The binding free energy analysis (Figure S20 and Table 9) shows that van der Waals and electrostatic interactions are the main stabilizing forces in C16–integrin complexes, while polar solvation opposes binding and nonpolar solvation provides minor support. Among the systems, αvβ3/S2 exhibited the most favorable Δ*G*_bind_ (−48.67 ± 11.7 kJ·mol^–1^), followed by α5β1/S1 (−35.41 ± 5.7 kJ·mol^–1^) and αvβ3/S1 (−28.61 ± 8.1 kJ·mol^–1^), indicating that the alternative S2 site of αvβ3 offers the most energetically optimized binding environment.

**Table 9:** Predicted Binding Energy for C16–Integrin Complexes

System	*G*_GAS_	*G*_SOLV_	Δ*G*_bind_
**α5β1/S1**	–622.77 ± 15.8	587.36 ± 16.8	**–35.41 ± 5.7**
**αvβ3/S1**	–575.35 ± 51.9	546.74 ± 57.2	**–28.61 ± 8.1**
**αvβ3/S2**	–663.67 ± 28.4	615.01 ± 17.4	**–48.67 ± 11.7**

For α5β1/S1, the balanced contributions of gas-phase and solvation energies (*G*_GAS_ = −622.77 kJ·mol^–1^; *G*_SOLV_ = 587.36 kJ·mol^–1^) suggest strong but partially compensated intermolecular interactions, consistent with persistent hydrogen bonds involving residues such as LEU10 and GLU916. This stable yet slightly solvent-exposed configuration aligns with the narrower conformational distribution observed during MD simulations.

Conversely, αvβ3/S1 displayed the least favorable Δ*G*_bind_, correlating with its greater conformational flexibility and weaker hydrogen bond occupancy at the MIDAS site. The smaller energy difference between *G*_GAS_ and *G*_SOLV_ further indicates limited stabilization. Overall, the energetic hierarchy (αvβ3/S2 < α5β1/S1 < αvβ3/S1) highlights the S2 site as the most stable binding mode, integrating hydrogen bonding and energetic analyses into a coherent model of C16–integrin recognition.

The stronger binding of C16 to the alternative αvβ3_S2 pocket suggests that this interaction may modulate integrin activation through a distinct, noncanonical mechanism. Unlike the classical MIDAS-associated S1 site, where ligand engagement directly supports conformational shifts toward the high-affinity or active state, the S2 pocket appears to stabilize C16 in a manner that could preferentially constrain integrin flexibility or impede the propagation of activating structural rearrangements. This stabilizing effect is consistent with the higher binding free energy and persistent hydrogen bond network observed at S2, which may reinforce local structural rigidity around the β-propeller and hybrid domains, thereby reducing the integrin’s capacity to transition into its extended, active conformation.

From a functional perspective, such selective stabilization at the αvβ3_S2 site could influence integrin-specific signaling and downstream cellular responses. The high-affinity interaction may favor a partially inactive or intermediate conformational state, fine-tuning αvβ3-mediated adhesion and signaling differently from α5β1. Given that αvβ3 integrin plays roles in angiogenesis, cell migration, and tumor progression, preferential targeting of the S2 pocket by C16 could represent a mechanism to selectively modulate these processes without fully triggering integrin activation. This site-preferential binding may thus underlie the distinct biological effects of C16, offering a structural rationale for its putative differential modulation of integrin pathways compared to classical RGD-like ligands.

The S2 pocket identified in this study corresponds to a previously uncharacterized site situated at a distinct αvβ3 interdomain interface. This region is structurally separate from the upper-surface allosteric hotspots targeted by macromolecular ligands such as CD40L^104^ and FGF1/FGF2,^105^ as well as from sites engaged by small-molecule modulators^106^ or lipid-based regulators.^107^ Importantly, recent cryo-EM studies of full-length αvβ3 revealed that the non-RGD small-molecule inhibitor CWHM-12 binds outside the canonical MIDAS-centered recognition mode and stabilizes a spectrum of intermediate and semiopen conformations, highlighting the existence of alternative regulatory interfaces beyond the classical RGD/MIDAS site.^108^ In this context, the distinctiveness of the S2 pocket reveals an additional layer of integrin regulation, showing that peptide binding at this previously unrecognized interface could propagate long-range conformational effects.

#### 3.7.5 Limitations of the Study

The conclusions of this work are derived exclusively from *in silico* analyses. Although our computational analyses provide new hypotheses regarding C16 recognition by αvβ3 and α5β1, some limitations must be acknowledged. This work relies on computational approaches, and therefore the predicted binding preferences, the proposed noncanonical S2 site in αvβ3, and the differential engagement of the two integrins should be experimentally validated.

The use of headpiece-only ectodomain models was appropriate for isolating the structural determinants of peptide recognition, but this strategy does not capture the long-range allosteric effects mediated by the transmembrane and cytoplasmic domains. Likewise, the 100 ns simulations, while adequate for evaluating local stability and short-time scale interactions, cannot fully resolve slower conformational transitions or rare events that would require extended simulations or full-length integrin models embedded in membranes.

Methodologically, MM-PBSA provides only semiquantitative estimates of relative binding affinities due to known limitations in its solvation models, and dielectric assumptions. In the present work, configurational entropy was not included in the MM-PBSA calculations. As a result, the reported binding free energies reflect enthalpic-dominated relative trends rather than absolute thermodynamic affinities. As an end-point method, it cannot capture kinetic barriers or full binding pathways, and therefore our energy results should be interpreted strictly as relative trends.

In this study, MM-PBSA was used as an initial comparative assessment of C16–integrin complex stability rather than for absolute affinity prediction, following the best-practice recommendations.^109^ Future work using enhanced-sampling free-energy methods, such as umbrella sampling, metadynamics, or alchemical approaches, will be necessary to fully resolve binding mechanisms and energy barriers.

Finally, the ADMET predictions reported in this study should be interpreted with caution, as the employed tools are primarily optimized for small molecules and provide only qualitative insights when applied to peptide systems. Experimental pharmacokinetic and permeability assays will be required to validate these *in silico* predictions.

These limitations highlight the essential need for targeted experimental validation to substantiate and refine our computational predictions. Follow-up studies such as competitive binding assays, SPR or ITC affinity measurements, peptide mutagenesis, and integrin site-directed mutagenesis are necessary to test the hypothesized binding determinants and confirm the existence and relevance of the proposed S2 site. Structural methods, such as hydrogen–deuterium exchange or cross-linking mass spectrometry could further evaluate the predicted peptide–integrin interfaces.

## 4 Conclusions

In this study, we employed an integrative computational strategy to elucidate the molecular mechanisms underlying the interaction between the laminin-1-derived C16 peptide and the αvβ3 and α5β1 extracellular matrix integrins. Through a combination of ADMET profiling, molecular docking, MD simulations, and normal-mode analyses, we characterized both the pharmacokinetic properties and structural determinants of C16 binding. These results provide a detailed atomistic understanding of how this bioactive peptide recognizes and stabilizes integrin conformations involved in cell adhesion, signaling, and angiogenesis.

The *in silico* pharmacological predictions revealed that the C16 peptide possesses favorable binding characteristics but limited drug-likeness due to its high molecular weight, flexibility, and hydrophilicity. Despite these limitations, its physicochemical profile supports strong and specific molecular recognition of integrin receptors, a property that may be exploited through formulation or delivery strategies. C16 displayed moderate plasma protein binding, low oral bioavailability, and minimal cytochrome P450 inhibition, suggesting a low potential for systemic toxicity and drug–drug interactions, yet emphasizing the need for nonoral delivery systems.

Molecular docking identified conserved interactions with the canonical MIDAS of both αvβ3 and α5β1 integrins, involving electrostatic and hydrophobic complementarity. Notably, a distinct noncanonical binding pocket (S2) was discovered in αvβ3, located opposite the MIDAS region. This putative alternative site forms an amphiphilic interface capable of accommodating the C16 peptide through extensive hydrophobic contacts and balanced charge complementarity, providing computational mechanistic insights for putative αvβ3-preferential binding selectivity.

MD simulations revealed that C16 binding stabilizes both integrins without inducing major conformational rearrangements, consistent with a modulatory rather than disruptive effect on receptor dynamics. The αvβ3 C16_S2 complex exhibited the greatest structural stability, reduced flexibility, and favorable predicted binding free energies, underscoring the thermodynamic preference for this novel site. PCA and normal-mode analyses confirmed that the dominant collective motions involved domain bending and twisting, with αvβ3 displaying greater dynamic adaptability upon C16 association than α5β1.

Altogether, our findings uncover a previously unrecognized αvβ3-preferential C16 putative binding site that may function as an alternative anchoring or allosteric interface. This dual binding behavior provides a new conceptual framework for understanding integrin putative subtype selectivity and ligand-mediated activation mechanisms. Beyond fundamental insight, the identification of this putative noncanonical site opens avenues for the rational design of next-generation peptide mimetics and inhibitors targeting αvβ3 with improved specificity and pharmacological performance in angiogenesis and metastatic cancer therapy.

## Supplementary Material





## Data Availability

The scripts used to run and analyze the MD simulations are available at: (https://github.com/DrFrank25/Gromacs-Step-by-step-tutorial). Additionally, the structures, configuration files, and MD simulation trajectories are provided and can be accessed directly at: (https://zenodo.org/records/14209630).

## Notes

Global structural analyses of integrin–C16 complexes, docking results, PC and normal mode comparisons, clustering, hydrogen bonds, C16 secondary structure evolution, and binding free energy decomposition results (PDF)
Molecular animations visualizing integrin dynamics: PC motions, normal mode displacements, and deformation vectors (ZIP)
