# Supplementary material for: Beyond MIDAS: An In Silico Study of a Putative Noncanonical C16 Binding Site in αvβ3 Integrin
Source: ACS Omega. 2026 Feb 19;11(8):13606–28. doi: 10.1021/acsomega.5c11287 (PMC12961469; doi:10.1021/acsomega.5c11287)
Supplement: Supplementary file 1 [file ao5c11287_si_001.pdf]

Supporting Information

Supplementary Figures and Tables

Beyond MIDAS: An In Silico Study of a Putative Non-Canonical C16 Binding Site in αvβ3 Integrin

Francisco das Chagas Pereira de Andrade[1,2,†], Yago Ferreira e Silva[3,4,†], Paulo Ricardo Batista[3,4,*], and Anderson Nogueira Mendes[1,2,*]

[1] Laboratory of Innovation in Science and Technology – LACITEC, Department of Biophysics and Physiology, Federal University of Piauí, 64049-550, Teresina, Piauí, Brazil

[2] Department of Biophysics and Physiology, Federal University of Piauí, Teresina 64049-550, Brazil

[3] Programa de Computação Científica, Vice-Presidência de Educação, Informação e Comunicação, Fundação Oswaldo Cruz. Av. Brasil 4365, Residência Oficial, Manguinhos. 21045-900, Rio de Janeiro, Brasil.

[4] Programa de Pós-graduação em Biologia Computacional e Sistemas, Instituto Oswaldo Cruz, Fundação Oswaldo Cruz. Av. Brasil 4365, Manguinhos. 21045-900, Rio de Janeiro, Brasil.

* E-mail: pbatista@fiocruz.br

* E-mail: anderson.mendes@ufpi.edu.br

† F.C.P.A. and Y.F.S contributed equally to this work.

Data and Software Availability

The scripts used to run and analyze the MD simulations are available at: (https://github.com/DrFrank25/Gromacs-Step-by-step-tutorial).

Additionally, the structures, configuration files, and MD simulations trajectories are provided and can be accessed directly at: (https://zenodo.org/records/14209630).

Supplementary Movie captions

Visualization of the PC1 to 7-derived motions from the concatenated trajectory comprising the triplicates from both integrins. Displacements are scaled to 5 Å. (PC1.gif, ..., PC7.gif)

Visualization of the Normal modes 1 to 5-derived motions calculated on the αvβ3 structure (Mode1.gif, … Mode5.gif).

Visualization of the transition between αvβ3 and α5β1 structures described by the linear combination of the first αvβ3 normal modes (defvec.gif)

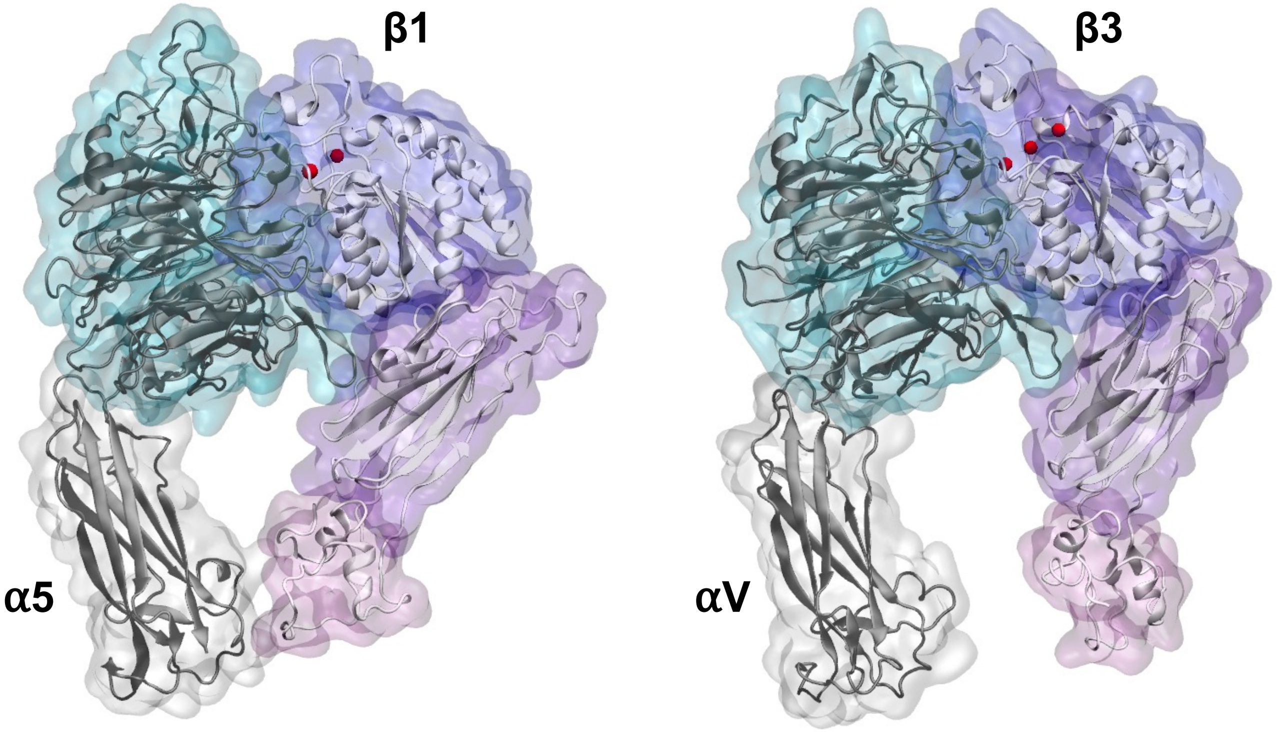

Subunit	Domain	αV (P06756)	α5 (P08648)	β3 (P05106)	β1 (P05556)
α	β-propeller	1–438	1–437	–	–
	Thigh	439–592	438–603	–	–
β	PSI	–	–	1–56	1–56
	Hybrid	–	–	57–128, 356–432	57–120, 353–432
	βI-like	–	–	129–355	121–352

Figure S1. Structural domains of the αvβ3 and α5β1 integrin headpiece. Cartoon and surface representations of the integrin headpieces for α5β1 (left) and αvβ3 (right). The α-subunits (α5 and αv) and β-subunits (β1 and β3) are colored according to their structural domains: β-propeller (cyan), thigh (light gray), PSI (pink), hybrid (violet), and βI-like (dark blue). Red spheres denote the metal ions at the MIDAS and ADMIDAS sites.

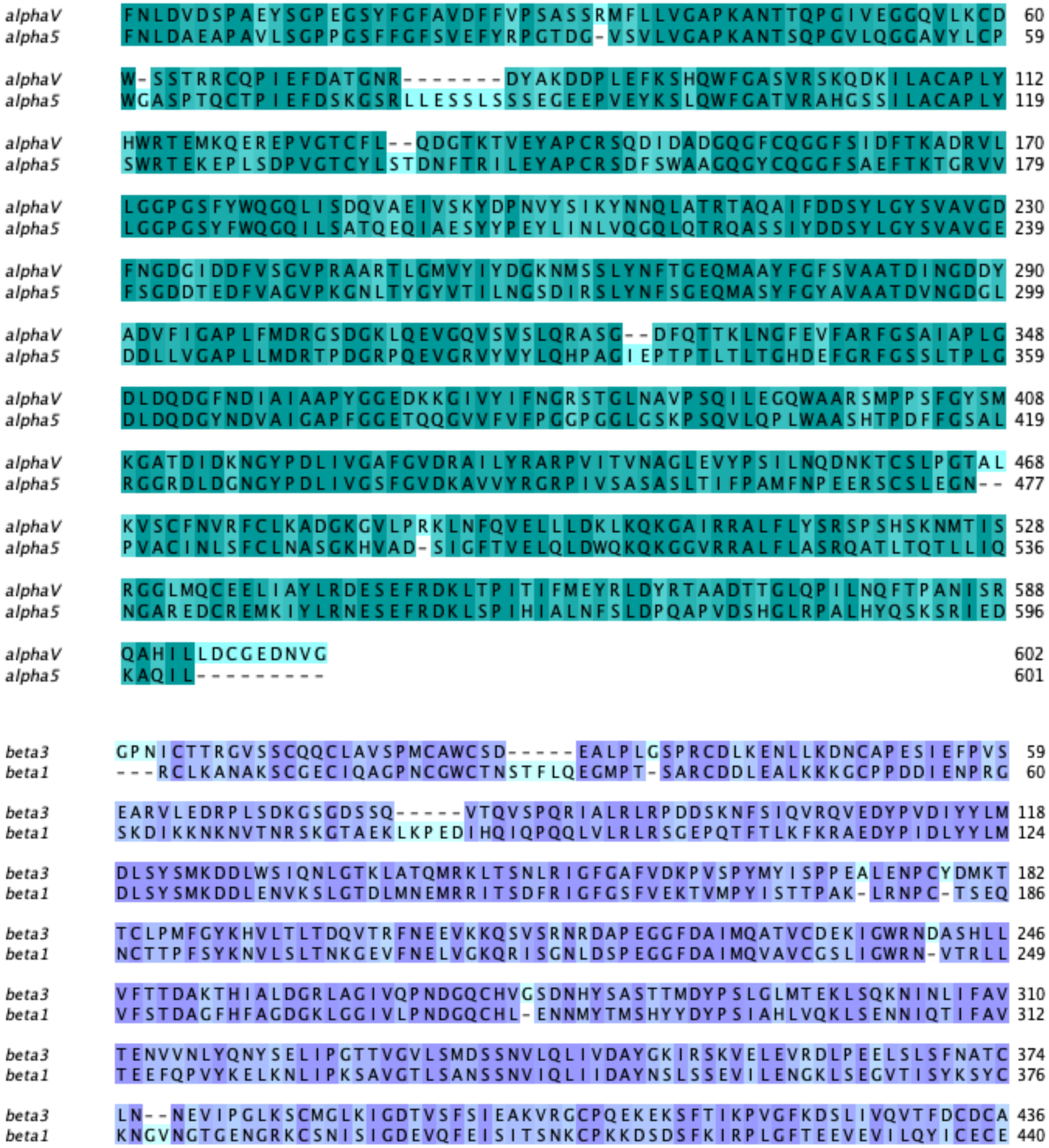

Figure S2. Multiple sequence alignment (MSA) of the α5β1 and αvβ3 integrin headpiece regions. The upper panel shows the alignment of α5 and αv subunits, while the lower panel displays the alignment of β1 and β3 subunits. Conserved residues are highlighted with increasing intensity according to their degree of conservation.

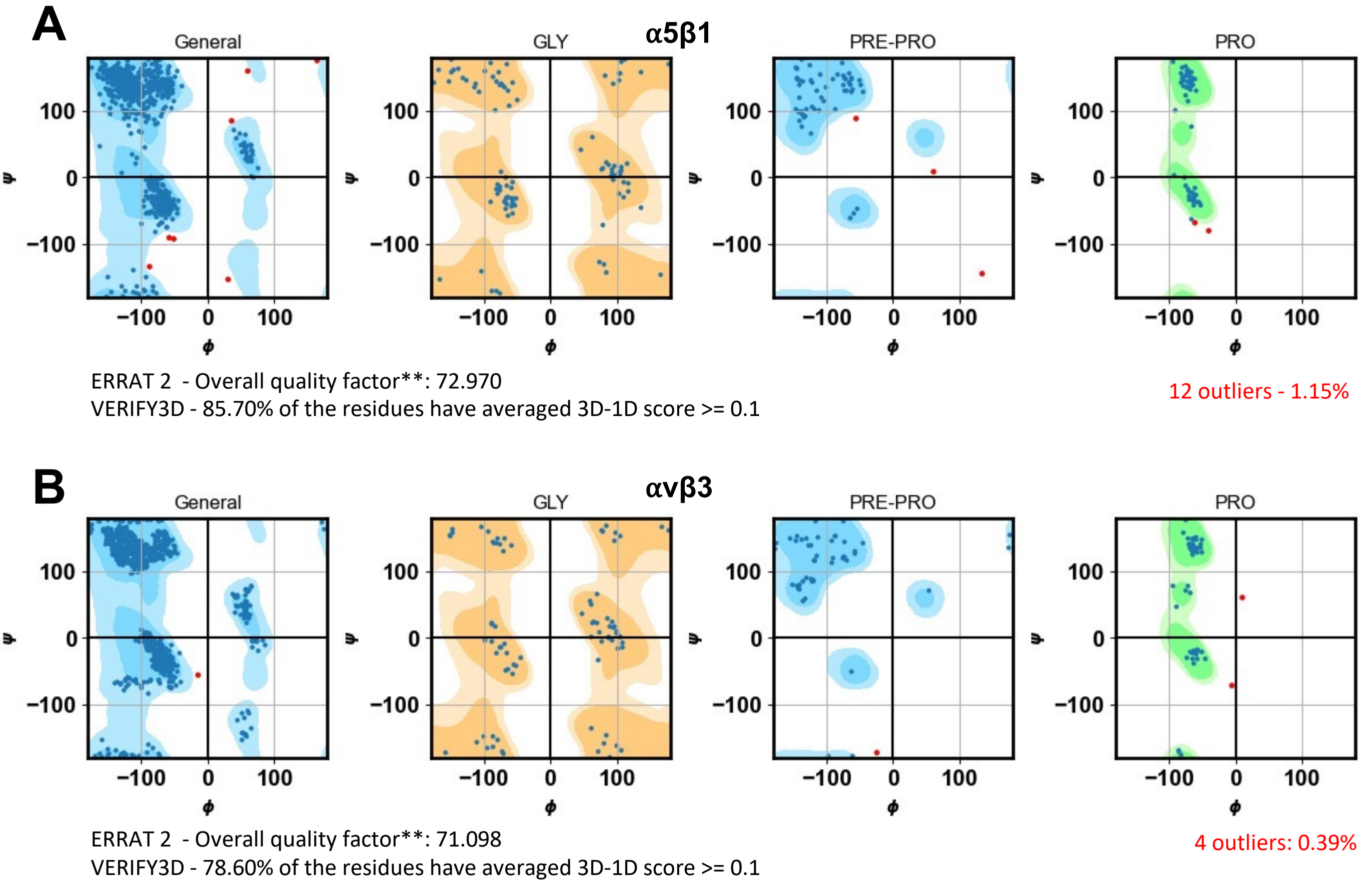

Figure S3. Validation of the integrin structural models. (A,B) The stereochemical quality of the final receptor structures was assessed and validated through Ramachandran plot (phi and psi dihedral distributions) analysis and the programs ERRAT2 and VERIFY3D. The overall quality factor from ERRAT2, the percentual of the residues have averaged 3D-1D score >= 0.1 in VERIFY3D is displayed in the left bottom of each plot. In red, the number (and %) of outliers (residues outside the allowed region of the Ramachandran Plot).

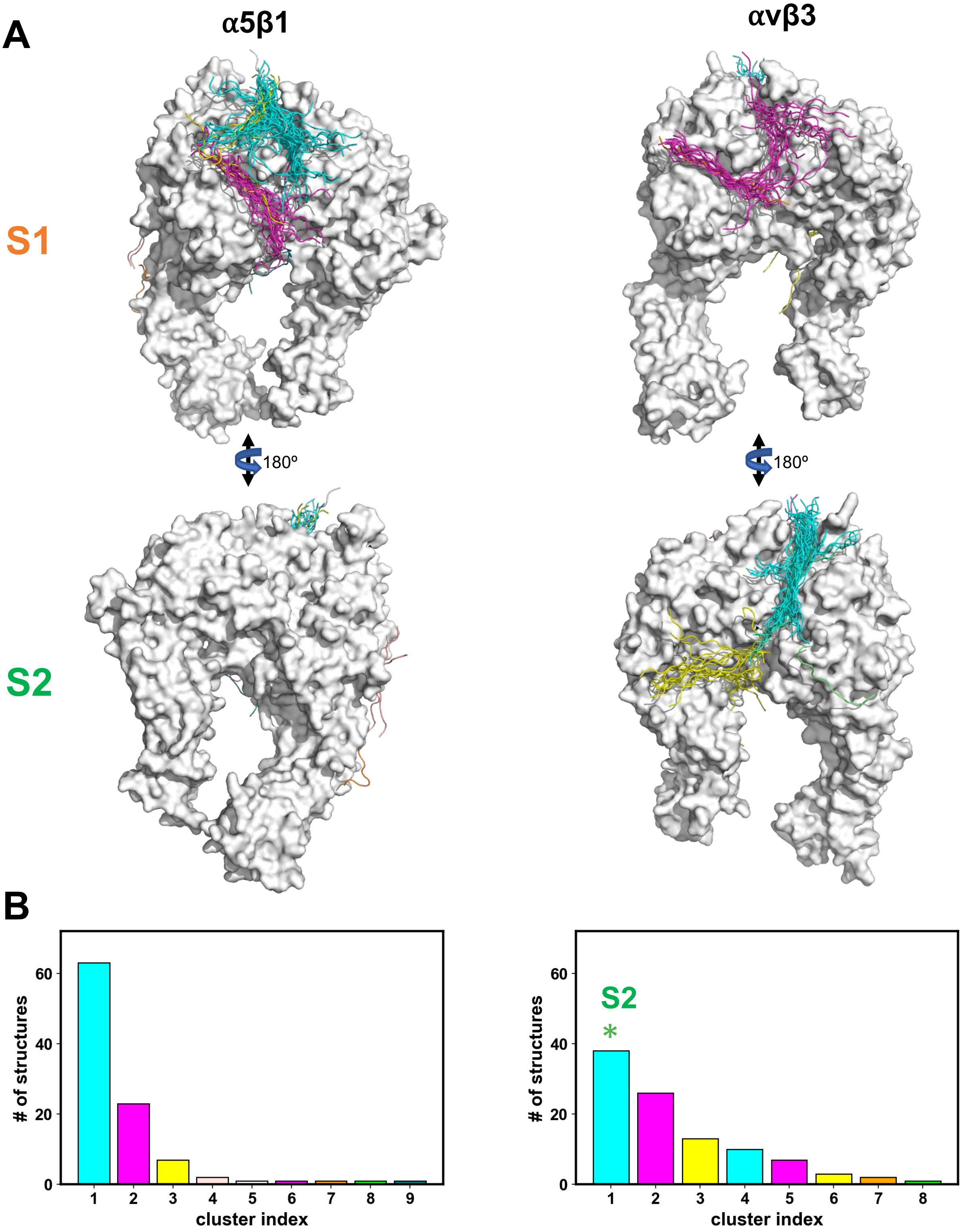

Figure S4. C16 peptide blind docking screening for α5β1 and αvβ3 integrins. (A,B) Representative binding clusters of the C16 peptide mapped on the integrin headpiece surface, highlighting the S1 (orange) and S2 (green) binding pockets. Each cluster is color-coded according to its cluster index. The dominant cluster for each binding site is indicated (A). Distribution of docked structures across the main clusters. The S2 pocket yielded the most populated clusters for both integrins, indicating a preferential binding site (B). The dominant clusters (cyan) correspond to the most favorable and recurrent binding poses, used as starting configurations for subsequent molecular dynamics simulations.

C

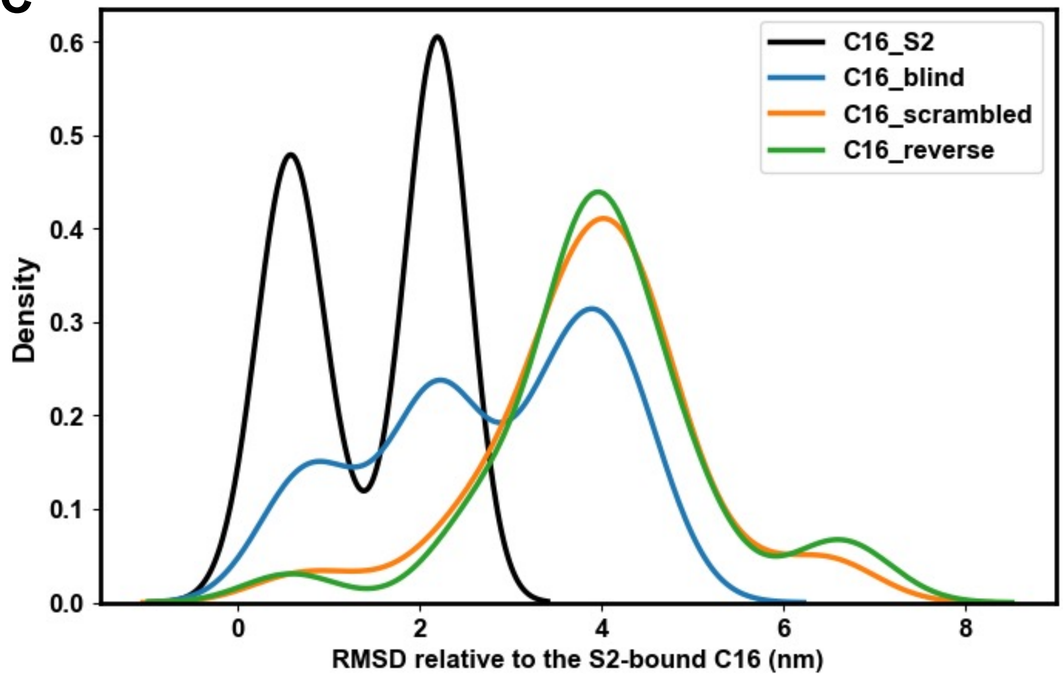

Figure S4_cont. (C) RMSD distributions relative to the S2-bound C16 reference pose. The RMSD distribution (nm) of four docking experiments was calculated using the lowest-energy C16 pose in the S2 pocket as structural reference. The datasets include: C16_S2 (C16 docked into the S2 pocket; black), C16_blind (blind docking across the entire αvβ3 headpiece; blue), C16_scrambled peptide (KRYLAFVKTDFI, scrambled control sequence; green), and C16_reverse peptide (FKLRYVITDFAK, reverse-sequence control; orange).

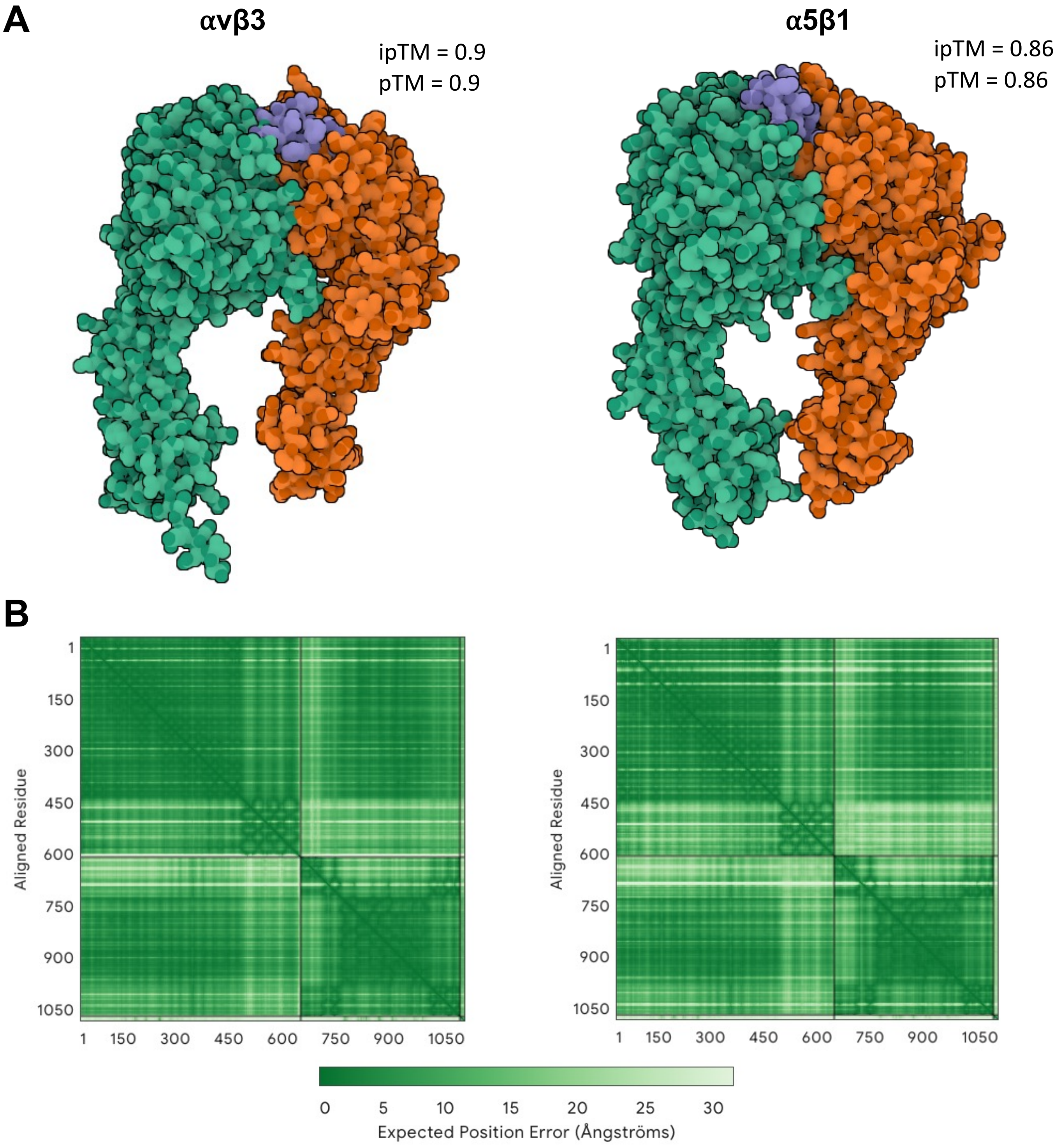

Figure S5. AlphaFold 3 prediction of the C16–integrin ternary complex. (A, B) Predicted structures of the αvβ3 (left) and α5β1 (right) integrin headpiece in complex with the C16 peptide (purple), obtained using AlphaFold 3. The α and β subunits are shown in green and orange, respectively (A). The predicted model confidence is indicated by the predicted template modeling score (pTM = 0.9) and interface predicted TM score (ipTM = 0.9), supporting the reliability of the peptide–integrin complex configuration. Predicted aligned error (PAE) plots showing residue-wise position uncertainty across each complex (B).

S1
(canonical binding site)

α5β1

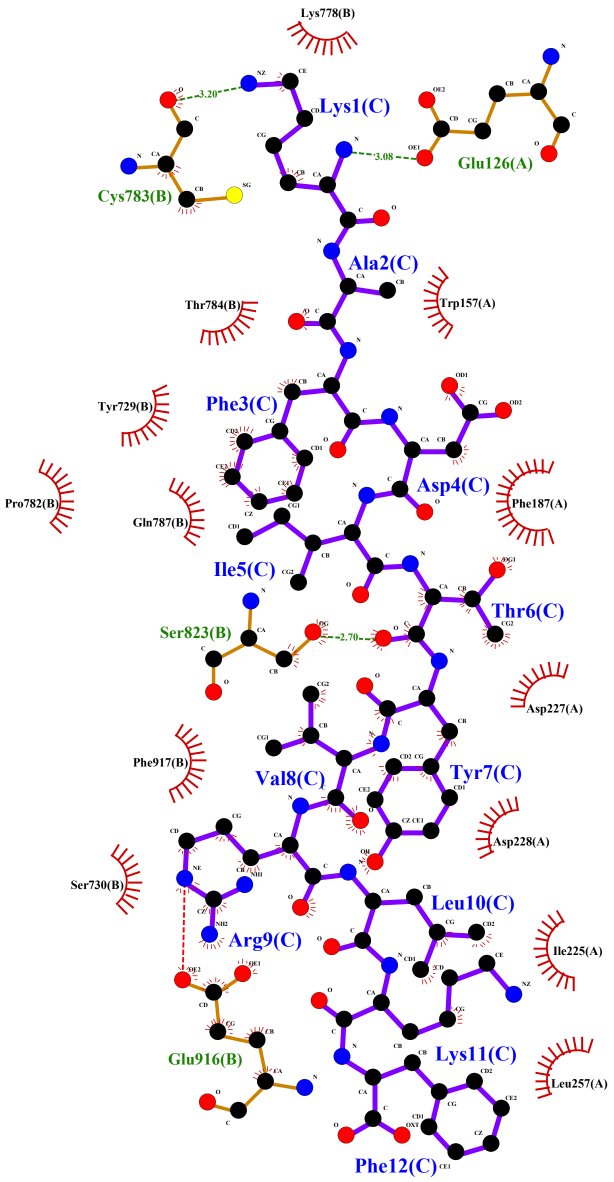

Figure S6. 2D interaction diagram of the C16–α5β1 interactions at the S1 binding site. LIGPLOT+ representation of key noncovalent interactions between the C16 peptide and the α5β1 integrin. Hydrogen bonds are shown as green dashed lines with corresponding distances (in Å), and hydrophobic contacts are represented by red spoked arcs around the interacting residues. Peptide residues are labeled in blue, and receptor residues in black.

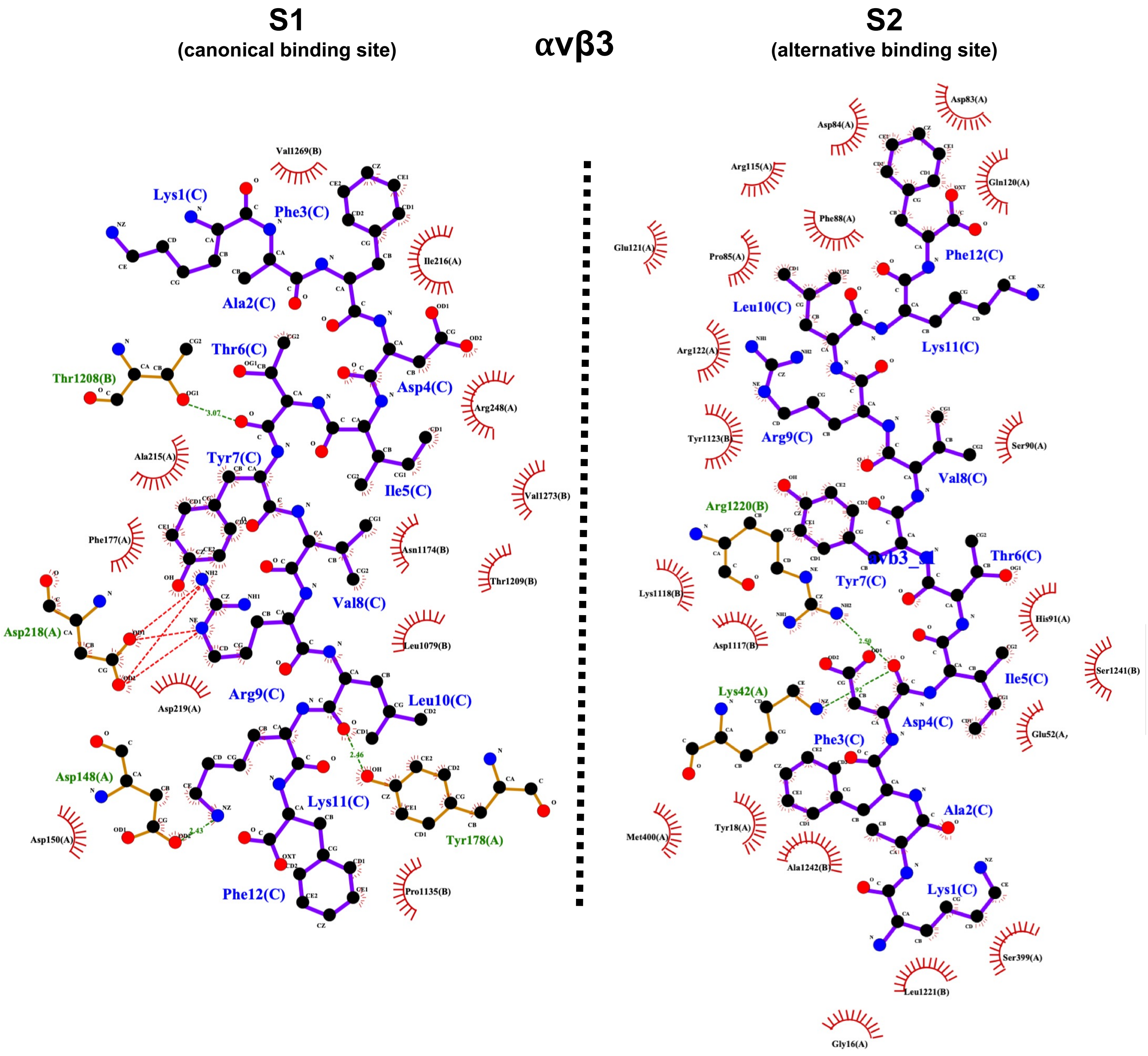

Figure S7. 2D interaction diagram of the C16–αvβ3 interactions at the S1 and S2 pockets. LIGPLOT+ representation of key noncovalent interactions between the C16 peptide and the αvβ3 integrin at the S1 (left) and S2 (right) binding sites. Hydrogen bonds are shown as green dashed lines with corresponding distances (in Å), and hydrophobic contacts are represented by red spoked arcs around the interacting residues. Peptide residues are labeled in blue, and receptor residues in black.

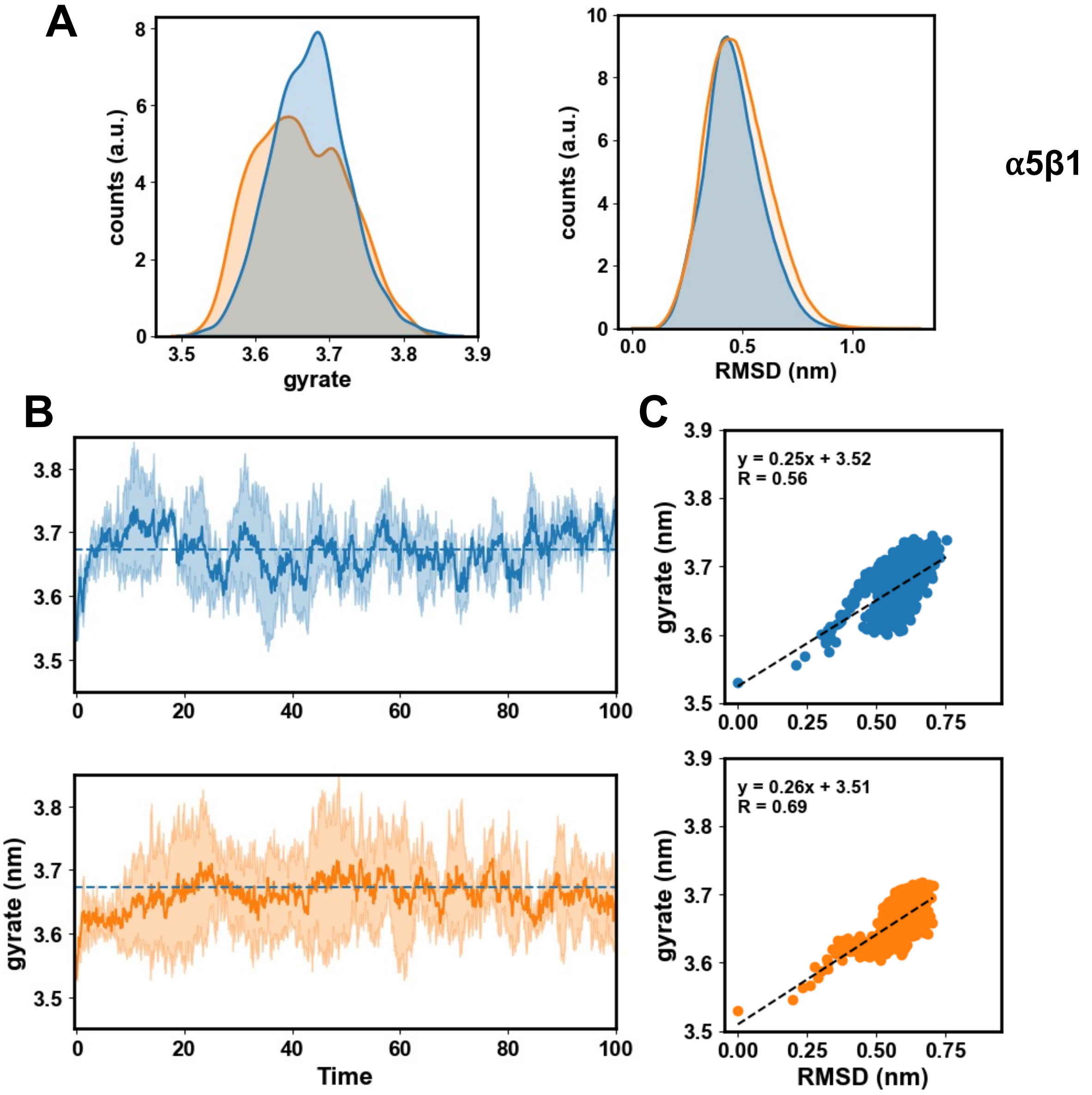

Figure S8. Structural compactness and conformational stability of the α5β1 integrin in the Apo and C16/S1 systems. (A) Probability distributions of the radius of gyration (left) and RMSD (right) for the unbound (Apo, blue) and C16-bound (C16/S1, orange) α5β1 integrin. (B) Time evolution of the radius of gyration (Rg) over 100 ns of simulation (left). (C) Correlation analysis between Rg and RMSD, showing a linear relationship that reflects coordinated conformational stability across the trajectories. Linear regression fits and R values are shown in each plot.

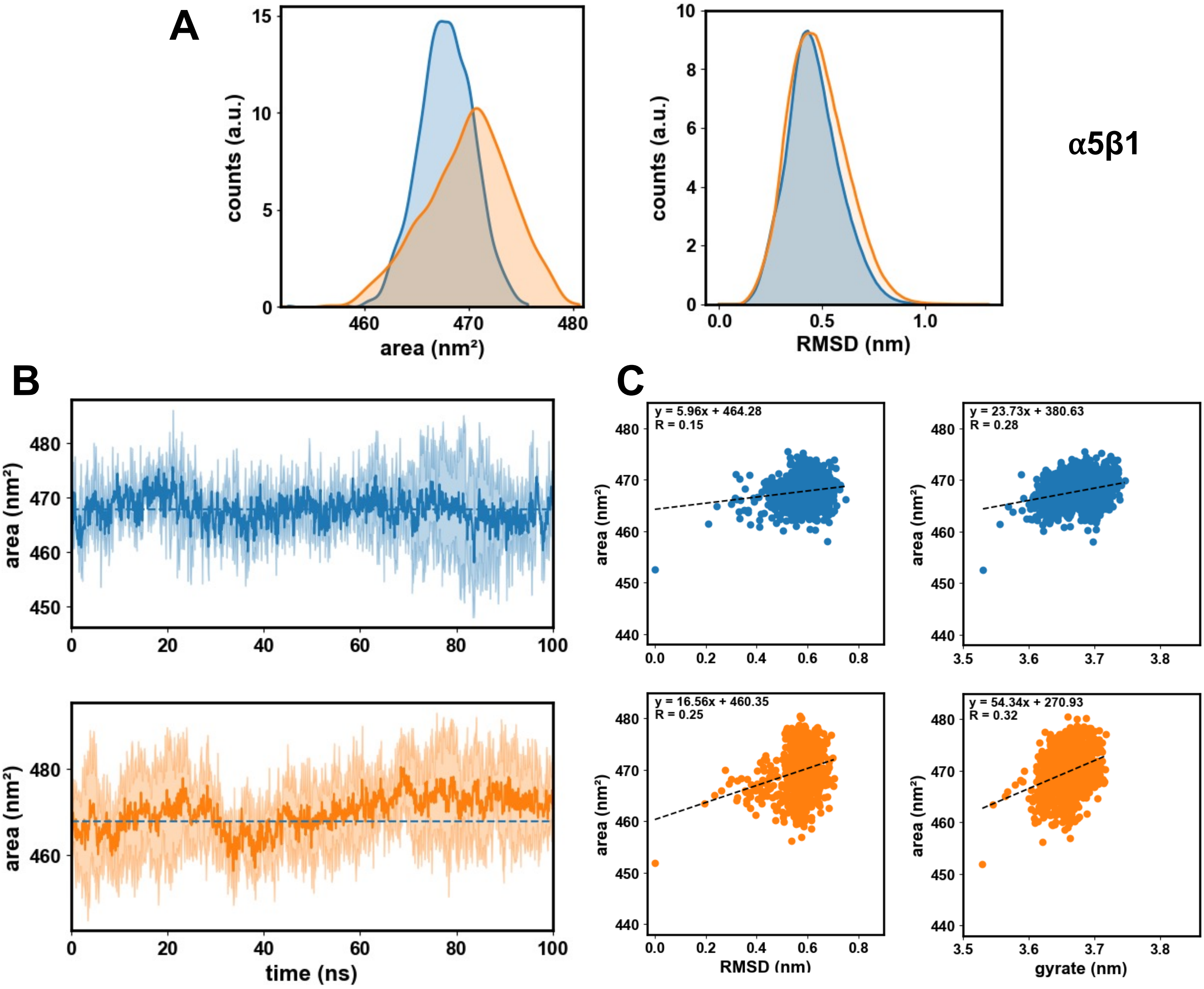

Figure S9. Solvent-accessible surface area (SASA) and conformational stability of the α5β1 integrin in the Apo and C16/S1 systems. (A) Probability distributions of the total SASA (left) and RMSD (right) for the unbound (Apo, blue) and C16-bound (C16/S1, orange) α5β1 integrin. (B) Time evolution of the SASA during the 100 ns simulations . (C) Correlation analyses between SASA and structural descriptors RMSD and Rg (right) for both systems. Linear regression fits and R values are shown in each plot.

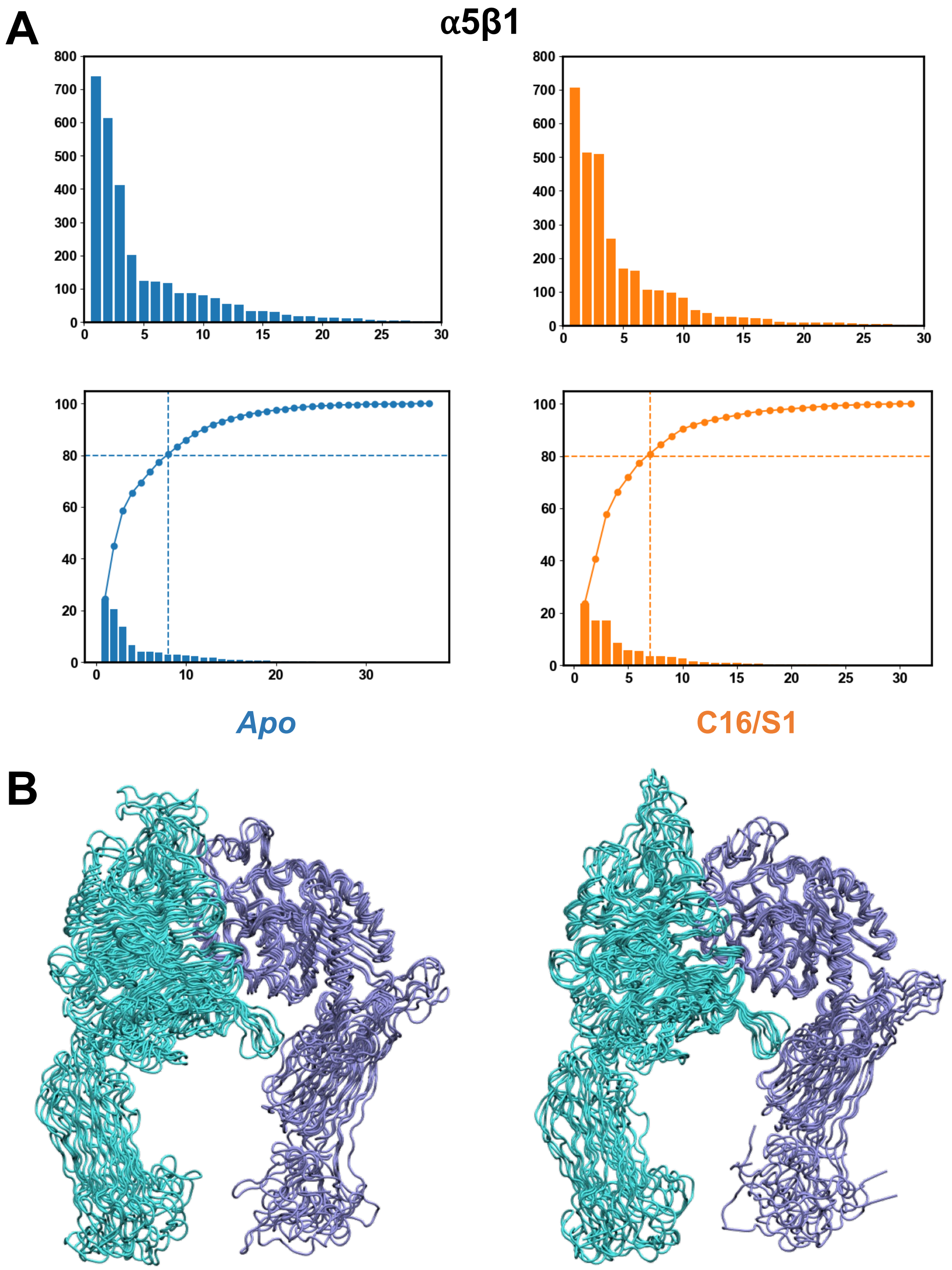

Figure S10. Clustering analysis of the α5β1 integrin from the Apo and C16/S1 molecular dynamics simulations. (A) Distribution of the number of structures per cluster ID (top) and cumulative percentage of conformations represented by each cluster (bottom). Left panels correspond to the unbound (Apo, blue) system, and right panels to the C16-bound (C16/S1, orange) system. (B) Superposition of the representative cluster centroids encompassing 80% of the total conformations sampled during the simulations, illustrating the dominant structural populations in each system.

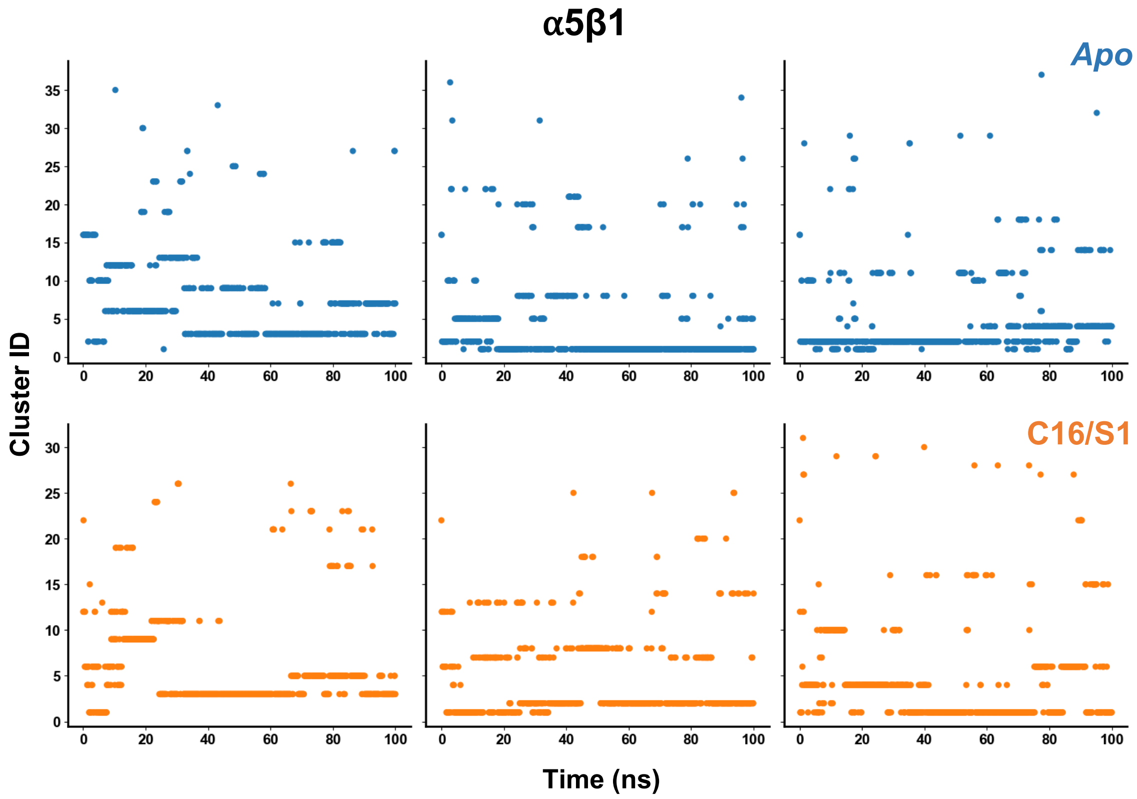

Figure S11. Temporal evolution of conformational clusters for α5β1 integrin in the Apo and C16/S1 systems. The plots show the cluster ID as a function of simulation time for each of the three independent replicas. The upper panels correspond to the unbound (Apo, blue) trajectories, and the lower panels to the C16-bound (C16/S1, orange) simulations. The triplicate trajectories were concatenated prior to clustering, ensuring that identical cluster IDs correspond to the same centroid conformation across all replicas.

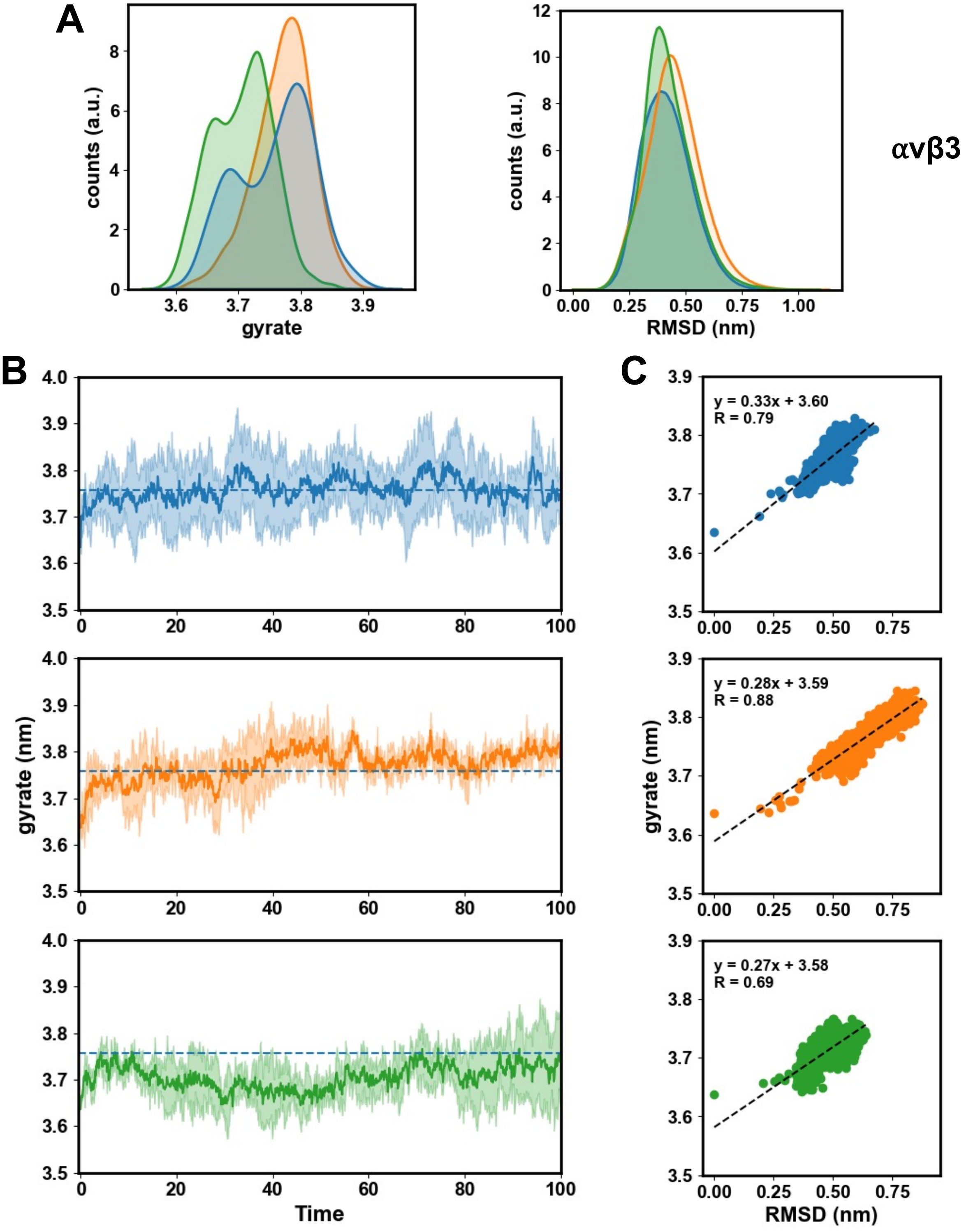

Figure S12. Radius of gyration (Rg) analysis of αvβ3 integrin in the Apo, C16/S1 and C16/S2 systems. (A) Probability distributions of the radius of gyration (left) and RMSD (right) for the apo (blue), C16/S1 (orange) and C16/S2 (green) simulations. (B) Time evolution of the radius of gyration over the 100 ns trajectories for each system (mean ± SD shown). (C) Scatter plots comparing Rg and RMSD for the three systems, illustrating the relationship between global compactness and conformational deviation over the sampled trajectories.

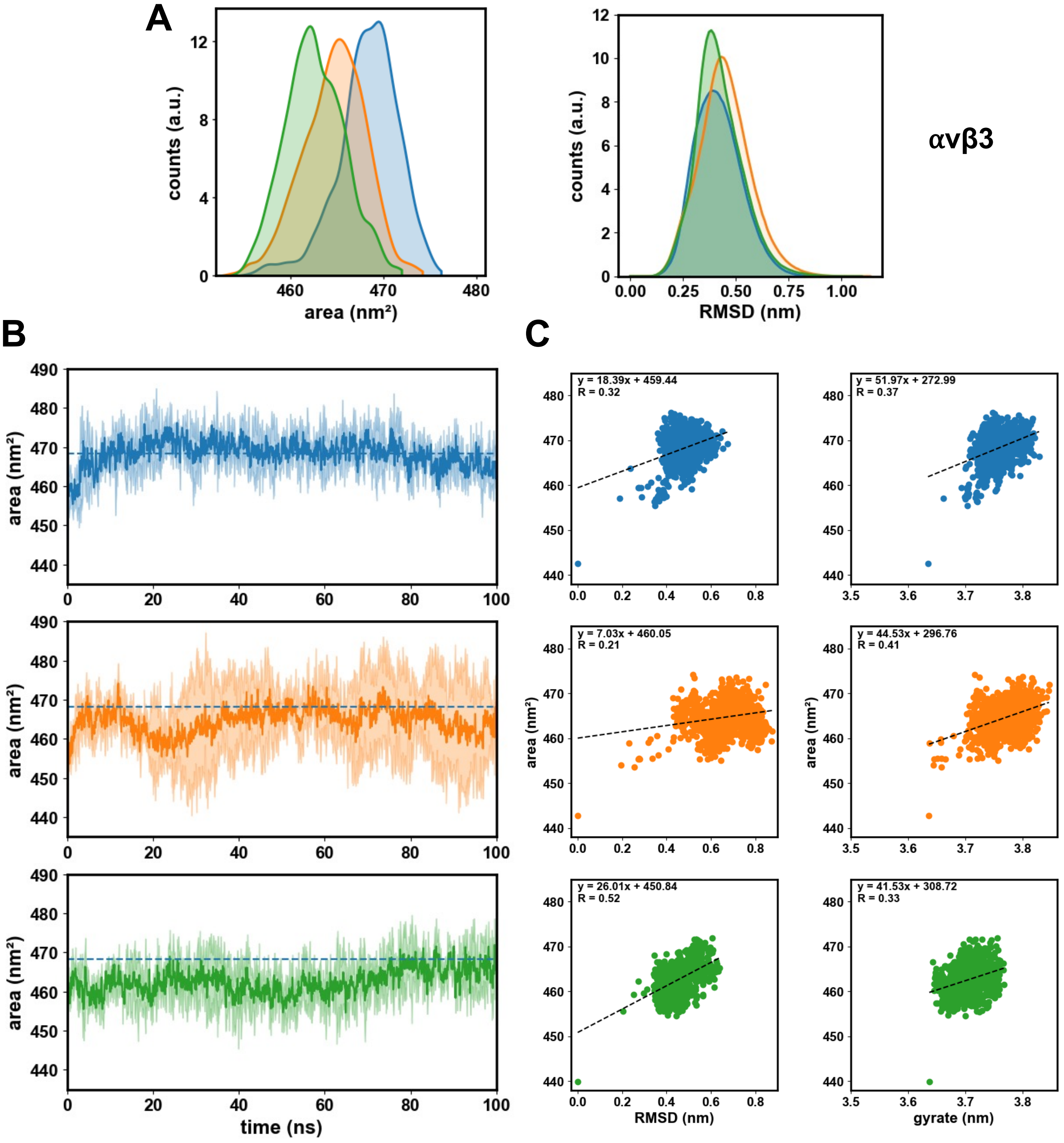

Figure S13. Solvent-accessible surface area (SASA) analysis of αvβ3 integrin in Apo, C16/S1, and C16/S2 systems. (A) Distributions of SASA (left) and RMSD (right) obtained from 100 ns MD trajectories. The apo, C16/S1, and C16/S2 simulations are shown in blue, orange, and green, respectively. (B) Time evolution of SASA across the trajectories showing the mean (solid line) and standard deviation (shaded area). (C) Correlation plots between SASA and RMSD (left) and between SASA and the radius of gyration (Rg, right). Linear regression and correlation coefficients (R) are shown for each system.

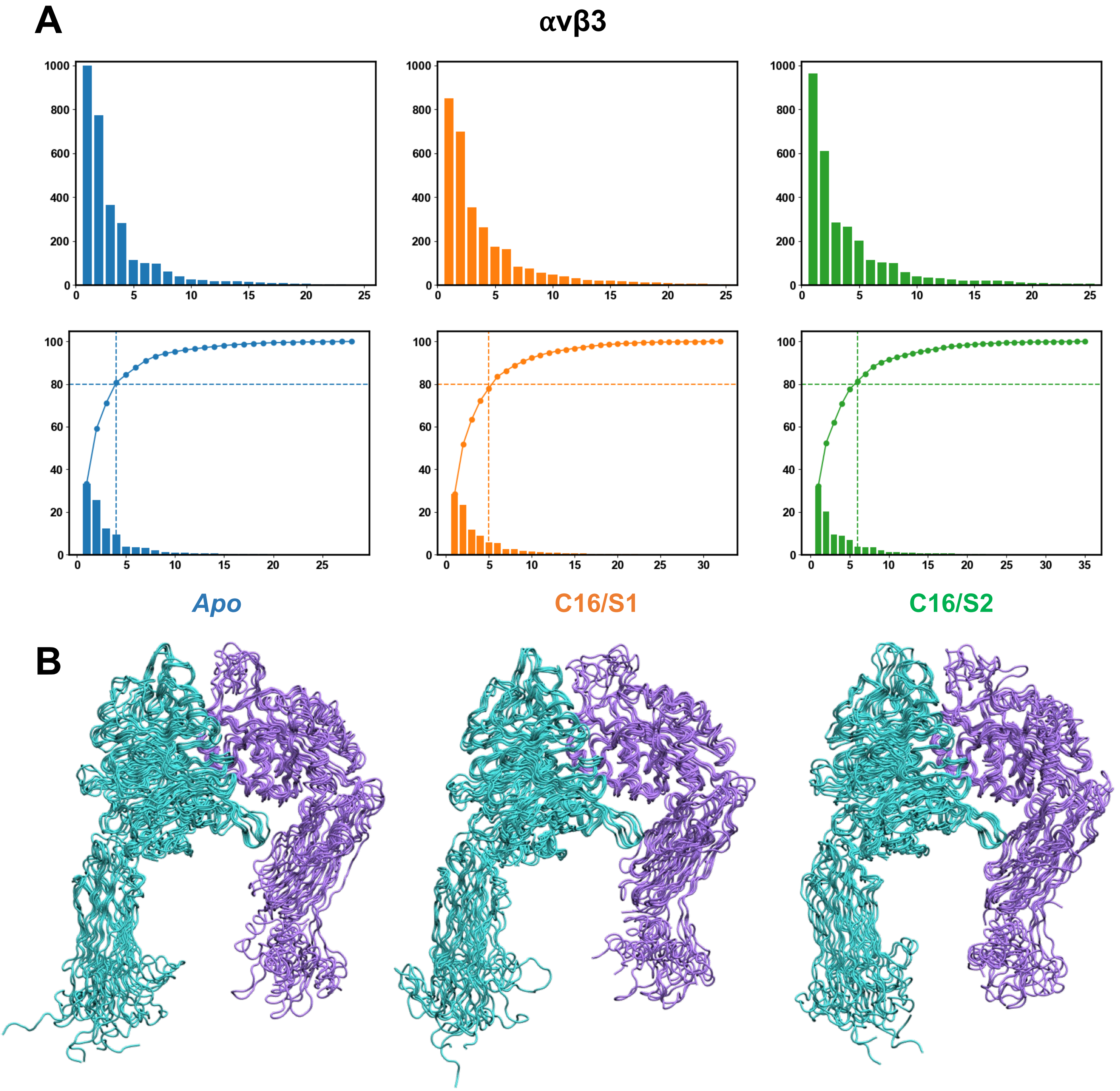

Figure S14. Clustering analysis of αvβ3 integrin during 100 ns MD simulations. (A,B) The figure shows the distribution of cluster populations derived from concatenated trajectories of three independent replicates for the Apo (blue), C16/S1 (orange), and C16/S2 (green) systems. (Top) Number of structures per cluster ID. (Bottom) Cumulative percentage of structures as a function of cluster ID, indicating the relative conformational diversity of each system.

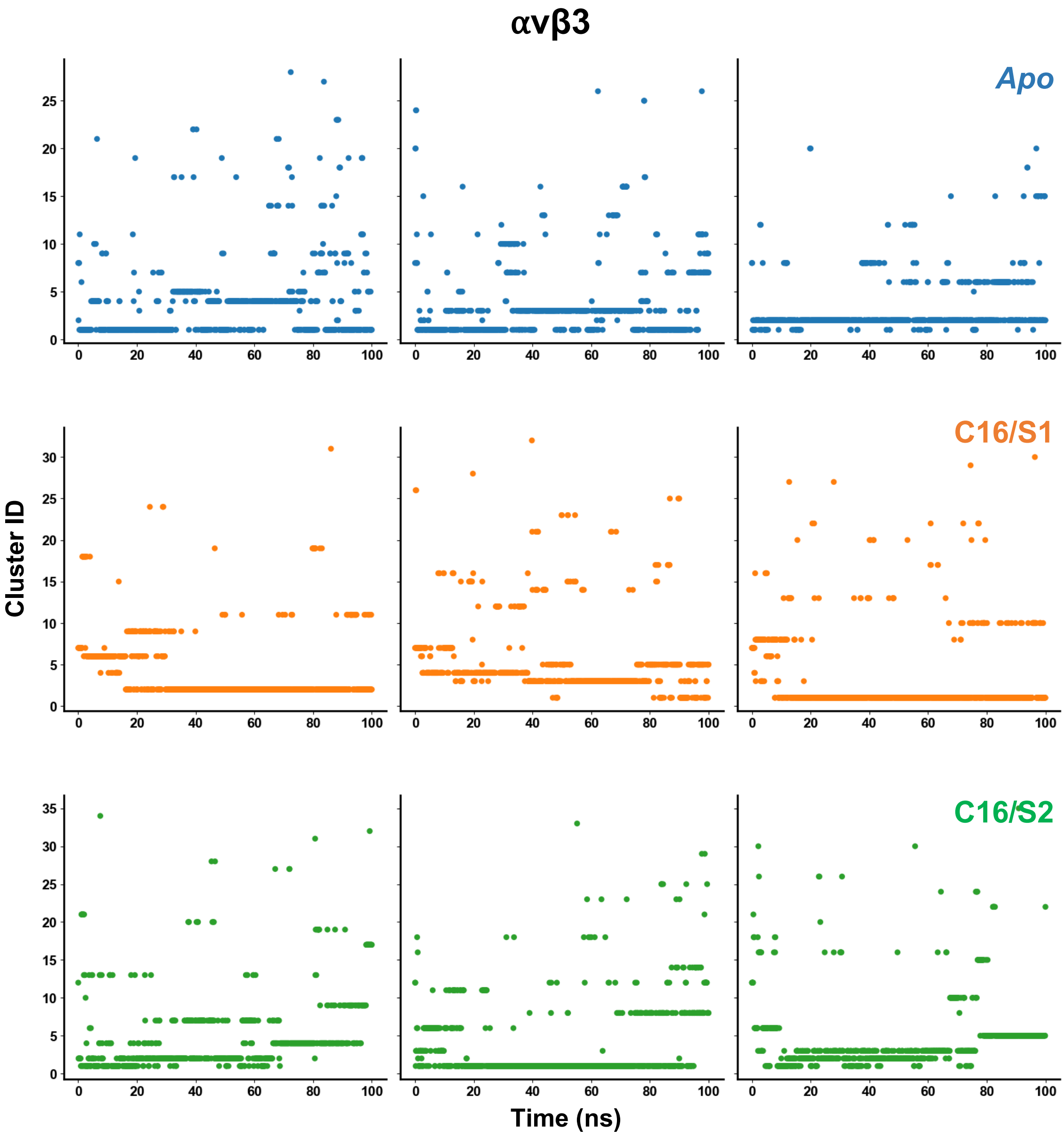

Figure S15. Time evolution of cluster IDs for αvβ3 integrin systems. The plots display the distribution of conformational clusters sampled during MD simulations of the Apo (blue), C16/S1 (orange), and C16/S2 (green) systems over time (100 ns). Each point corresponds to the cluster assignment at a given frame, highlighting the temporal persistence and interconversion of conformational states. This figure is analogous to Figure S11, which presents the same analysis for the α5β1 integrin systems.

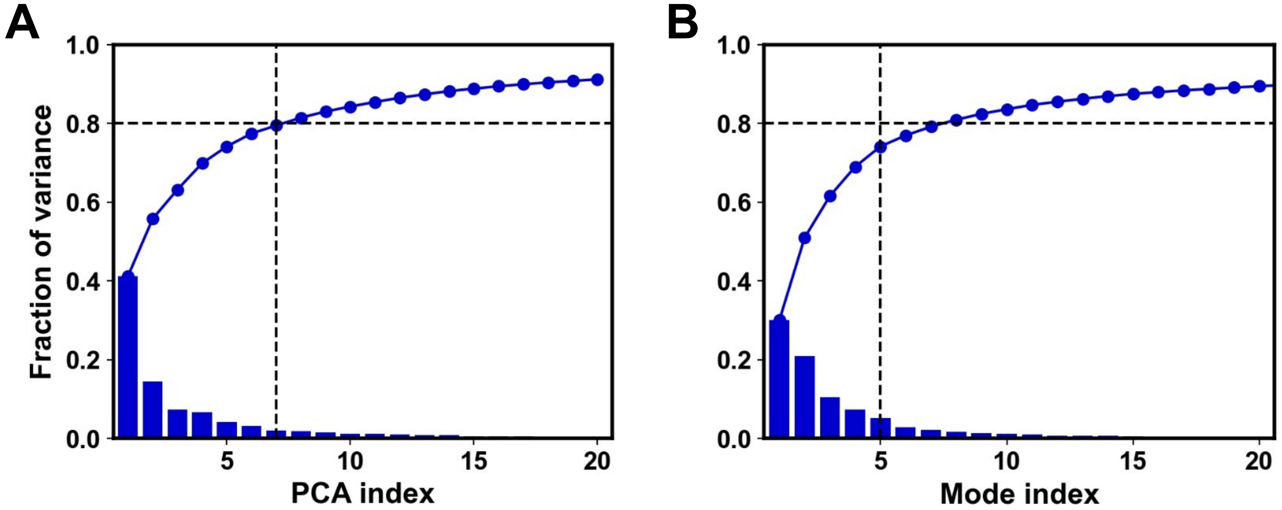

C

	Mode 1	Mode 2	Mode 3	Mode 4	Mode 5
PC1	0.66	0.25	0.25	0.01	0.22
PC2	0.1	0.89	0.05	0.11	0.16
PC3	0.57	0.16	0.21	0.08	0.3
PC4	0.13	0.09	0.8	0.07	0.04
PC5	0.35	0.04	0.17	0.1	0.1
PC6	0.03	0.06	0.02	0.8	0.22
PC7	0.04	0.1	0.1	0.32	0.49

D

	Mode 1	Mode 1-2	Mode 1-3	Mode 1-4	Mode 1-5
PC1	0.66	0.71	0.75	0.75	0.78
PC2	0.1	0.89	0.9	0.9	0.92
PC3	0.57	0.59	0.62	0.63	0.69
PC4	0.13	0.16	0.82	0.82	0.82
PC5	0.35	0.35	0.39	0.4	0.41
PC6	0.03	0.07	0.07	0.81	0.84
PC7	0.04	0.11	0.15	0.35	0.6

Figure S16. Comparison between PCs from MD simulations and normal modes calculated for αvβ3 integrin. (A) Fraction of total variance explained by the first 20 PCs, with individual contributions shown as bars and cumulative variance as a dotted line. (B) Equivalent representation for the first 20 normal modes obtained from normal mode analysis. (C) Overlap matrix between the first seven principal components (PC1–PC7) and the first five normal modes (Modes 1–5), showing the degree of correspondence between essential motions derived from MD and NMA. (D) RMSIP between subspaces spanned by normal modes (1–5) and PCs, indicating how efficiently the low-frequency modes reproduce the essential dynamics captured by MD simulations.

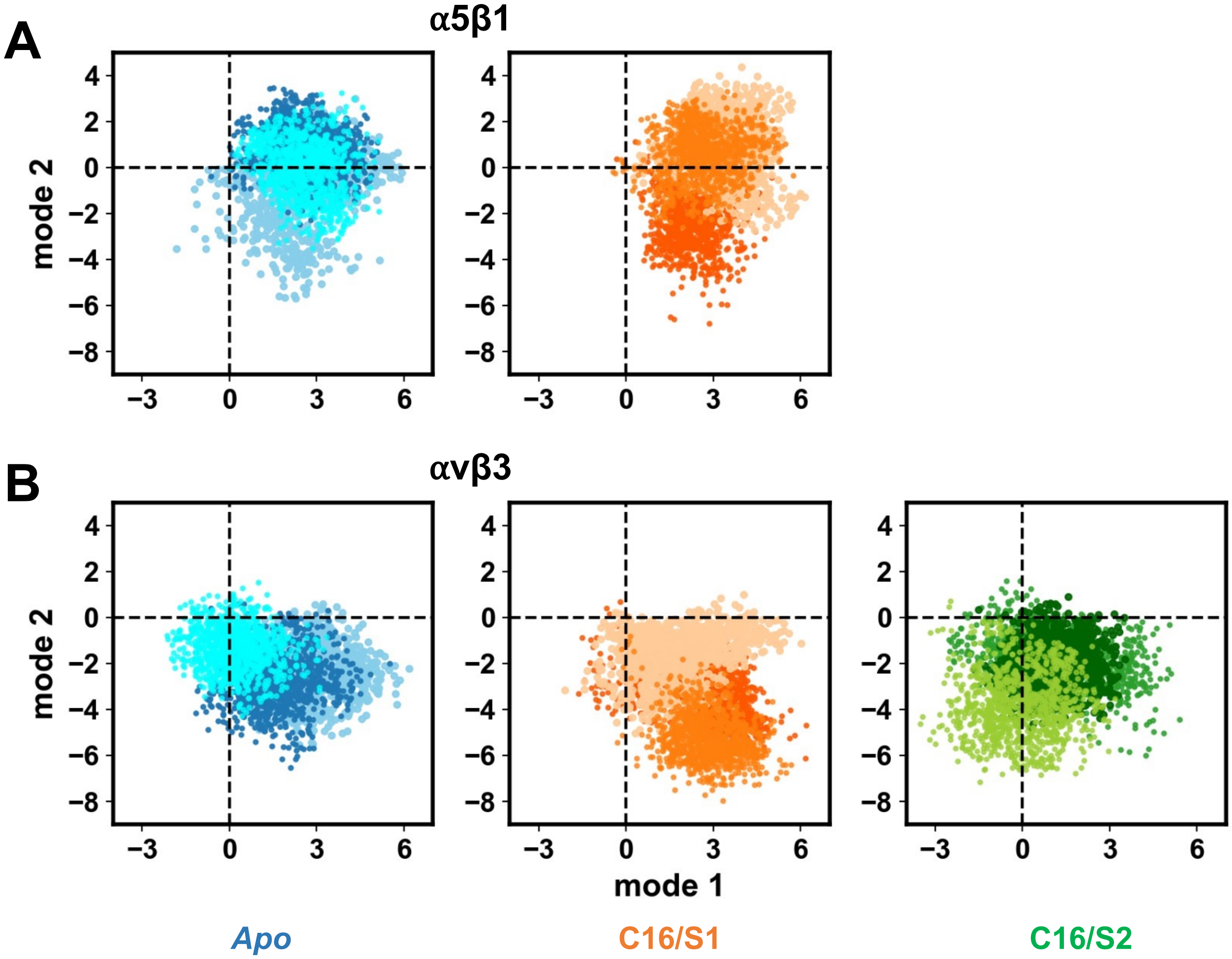

Figure S17. Projection of the integrin MD trajectories onto the two lowest-frequency avb3 normal mode space. (A,B) Projections of the α5β1 integrin trajectories onto mode 1 versus mode 2 (A). Projections of the αvβ3 integrin trajectories onto the same modes (B). The three independent MD replicates for each system are shown in shades of blue (apo), orange (C16/S1), and green (C16/S2), illustrating the conformational subspace explored during the simulations.

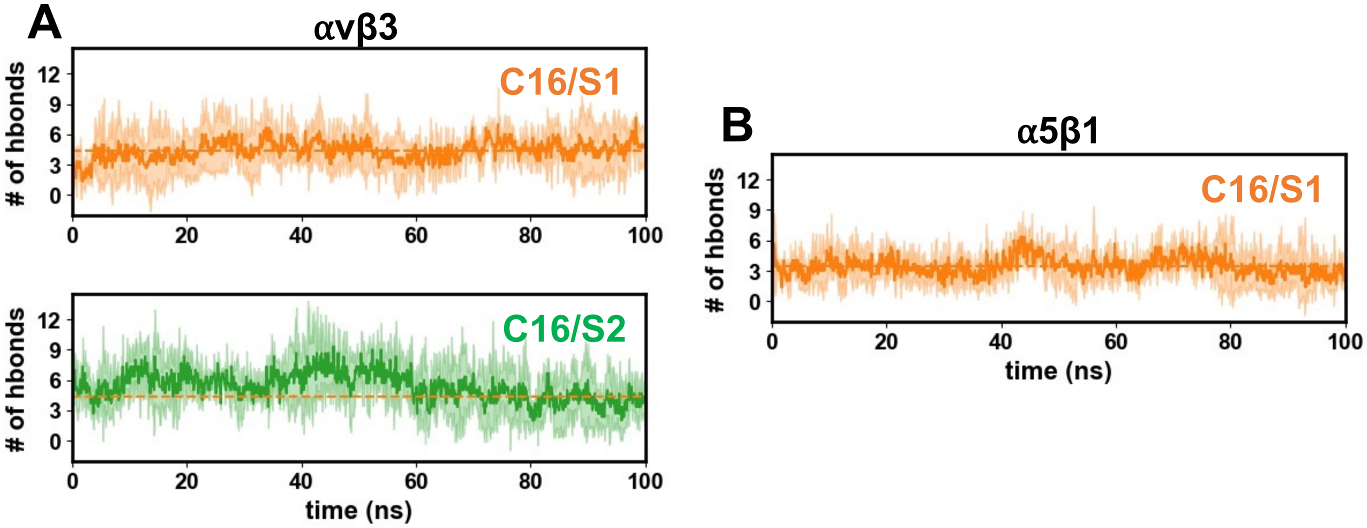

Figure S18. Number of hydrogen bonds between the C16 peptide and integrins during the MD simulations. (A, B) Hydrogen bond dynamics of the C16 peptide complexed with αvβ3 integrin at the S1 (orange) and S2 (green) binding sites (A). Hydrogen bond profile of the C16 peptide complexed with α5β1 integrin at the S1 site (B). Solid lines represent the average number of hydrogen bonds, and shaded areas indicate the standard deviation among triplicate simulations.

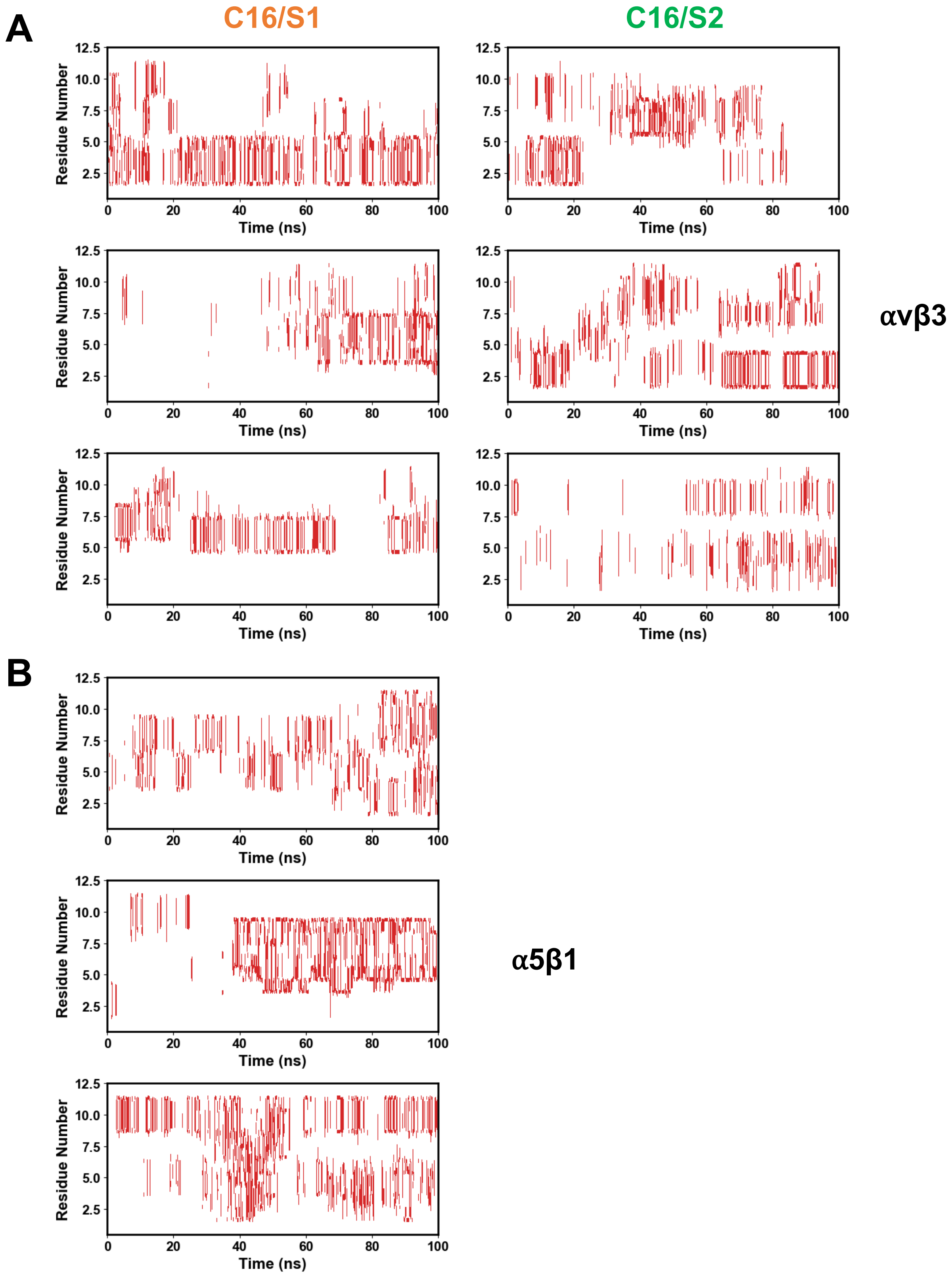

Figure S19. Time evolution of DSSP secondary structure elements during MD simulations of integrins complexed with the C16 peptide. (A,B) Temporal evolution of β-strand elements (highlighted in red) for the C16/αvβ3 (left, S1 pocket, orange) and (right, S2 pocket, green) (A). Time evolution of secondary structure of C16/α5β1 (S1 pocket). The plot is shown for the three different replicas over 100 ns of MD simulations.

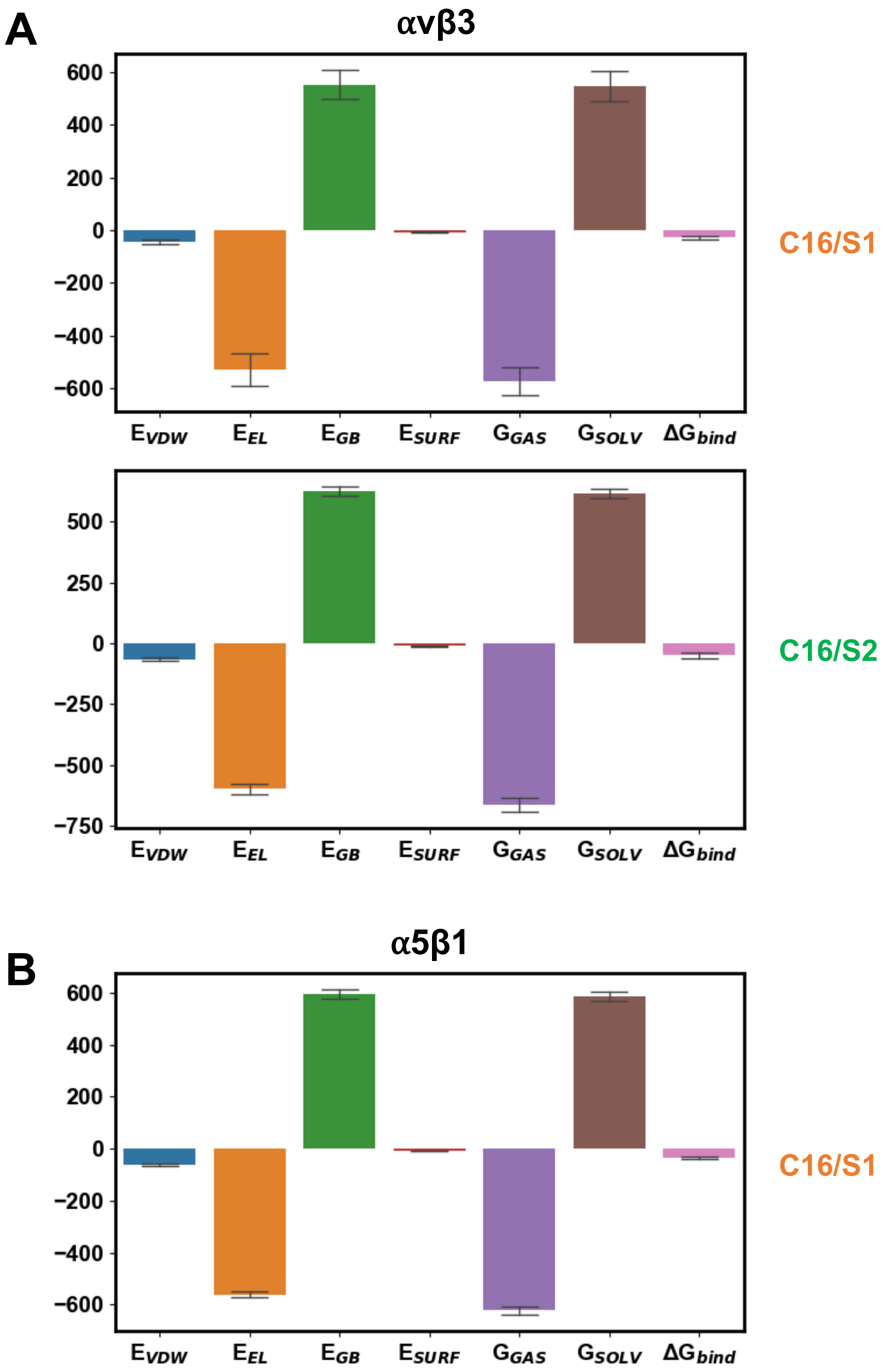

Figure S20. Contribution of energetic components to the binding free energy of the C16/integrin complexes. (A,B) The terms represent the van der Waals energy (E_VDW), electrostatic energy (E_EL), polar solvation energy (E_GB), nonpolar solvation energy (E_SURF), total gas-phase energy (G_GAS), solvation free energy (G_SOLV), and total binding free energy (ΔG_bind).

Target Name	ChEMBL ID	Indication	ICD-11 Code	Probability	Model Accuracy
Neuronal acetylcholine receptor; α4/β4	T73724	Alzheimer disease	8A20	98.13%	100%
		Aneurysm	BD51.Z	98.13%	100%
		Tobacco dependence	6C4A.2	98.13%	100%
Cathepsin D	T67102	Multiple sclerosis	8A40	99.96%	98.95%
Cathepsin L	T41141	Bone cancer	2B5Z	99.32%	96.61%
		Cancer-related pain	MG30	99.32%	96.61%
		Glioma	2A00.0	99.32%	96.61%
		Multiple sclerosis	8A40	99.32%	96.61%
		Solid tumour/cancer	2A00–2F9Z	99.32%	96.61%
Delta opioid receptor	T58992	Major depressive disorder	6A70.3	98.31%	99.35%
		Migraine	8A80	98.31%	99.35%
		Pain	MG30MG3Z	98.31%	99.35%
		Substance use disorder	6C4Z	98.31%	99.35%
		Rheumatoid arthritis	FA20	98.31%	99.35%

Table S1. **Ligand-based target prediction for C16 peptide.** Table presenting therapeutic targets associated with muscular, degenerative, inflammatory conditions, or cancer, filtering for targets with an association probability greater than 98% and a model accuracy exceeding 98%. It includes columns for the Target Name, ChEMBL-ID, Indication (clinical condition or disease with ICD-11 code), Probability (likelihood of association), and Model Accuracy (predictive precision).

α5β1				αvβ3							
C16/S1				C16/S1				C16/S2			
Donor_id	Acceptor_id	Occupancy (%)	Replica	Donor_id	Acceptor_id	Occupancy (%)	Replica	Donor_id	Acceptor_id	Occupancy (%)	Replica
**LEU10_N**	**GLU916_O**	**95.9**	**run3**	**SER123_N**	**ASP4_OD1**	**93.9**	**run3**	**TYR7_OH**	**ALA263_O**	**83.8**	**run3**
LEU10_N	GLU916_O	82.9	run1	SER123_N	ASP4_OD1	2.0	run2	TYR7_OH	ALA263_O	15.6	run2
LEU10_N	GLU916_O	23.4	run2	SER123_N	ASP4_OD1	0.0	run1	TYR7_OH	ALA263_O	8.5	run1
**LEU10_HG**	**GLU916_O**	**73.8**	**run3**	**SER121_OG**	**ASP4_OD1**	**93.1**	**run3**	**TRP93_NE1**	**TYR7_OH**	**60.3**	**run3**
LEU10_HG	GLU916_O	20.3	run2	SER121_OG	ASP4_OD1	16.1	run2	TRP93_NE1	TYR7_OH	50.3	run1
LEU10_HG	GLU916_O	19.1	run1	SER121_OG	ASP4_OD1	0.0	run1	TRP93_NE1	TYR7_OH	23.9	run2
**ARG9_HA**	**GLU916_HB1**	**66.6**	**run3**	**SER123_OG**	**ASP4_OD1**	**90.5**	**run3**	**LYS1_N**	**ASP84_OD2**	**49.7**	**run1**
ARG9_HA	GLU916_HB1	12.1	run2	SER123_OG	ASP4_OD1	0.2	run2	LYS1_N	ASP84_OD2	22.5	run2
ARG9_HA	GLU916_HB1	11.3	run1	SER123_OG	ASP4_OD1	0.0	run1	LYS1_N	ASP84_OD2	10.4	run3
**TYR7_OH**	**ASP855_OD1**	**57.7**	**run2**	**ALA2_N**	**ASP150_O**	**84.6**	**run1**	**LYS1_N**	**ASP84_OD1**	**44.7**	**run1**
TYR7_OH	ASP855_OD1	0.0	run1	ALA2_N	ASP150_O	0.0	run2	LYS1_N	ASP84_OD1	27.0	run3
TYR7_OH	ASP855_OD1	0.0	run3	ALA2_N	ASP150_O	0.0	run3	LYS1_N	ASP84_OD1	22.8	run2
**ARG9_HB1**	**GLU916_HG2**	**57.3**	**run3**	**SER123_OG**	**ASP4_CG**	**77.4**	**run3**	**GLN267_NE2**	**LYS11_O**	**44.3**	**run3**
ARG9_HB1	GLU916_HG2	3.2	run1	SER123_OG	ASP4_CG	0.1	run2	GLN267_NE2	LYS11_O	41.1	run1
ARG9_HB1	GLU916_HG2	2.1	run2	SER123_OG	ASP4_CG	0.0	run1	GLN267_NE2	LYS11_O	3.5	run2
**LEU10_HB2**	**GLU916_O**	**53.0**	**run3**	**ASN313_ND2**	**TYR7_O**	**68.1**	**run3**	**GLY264_CA**	**TYR7_HH**	**43.8**	**run3**
LEU10_HB2	GLU916_O	16.9	run1	ASN313_ND2	TYR7_O	39.1	run2	GLY264_CA	TYR7_HH	9.6	run2
LEU10_HB2	GLU916_O	10.7	run2	ASN313_ND2	TYR7_O	0.8	run1	GLY264_CA	TYR7_HH	4.9	run1
**VAL8_N**	**SER823_OG**	**51.1**	**run2**	**LYS1_NZ**	**ASP148_OD2**	**60.8**	**run3**	**HSD91_ND1**	**ILE5_O**	**43.2**	**run3**
VAL8_N	SER823_OG	50.6	run1	LYS1_NZ	ASP148_OD2	5.6	run2	HSD91_ND1	ILE5_O	0.0	run1
VAL8_N	SER823_OG	0.0	run3	LYS1_NZ	ASP148_OD2	0.0	run1	HSD91_ND1	ILE5_O	0.0	run2
**ASP822_HN**	**ILE5_HG12**	**49.4**	**run3**	**TYR178_OH**	**PHE3_O**	**60.5**	**run1**	**LYS1_N**	**ASP84_CG**	**41.1**	**run1**
ASP822_HN	ILE5_HG12	1.0	run1	TYR178_OH	PHE3_O	0.0	run2	LYS1_N	ASP84_CG	21.8	run2
ASP822_HN	ILE5_HG12	0.0	run2	TYR178_OH	PHE3_O	0.0	run3	LYS1_N	ASP84_CG	12.4	run3
**ARG9_HB2**	**GLU916_HG2**	**48.6**	**run3**	**LYS1_NZ**	**ASP150_OD2**	**60.3**	**run1**	**ARG122_NH2**	**PHE3_O**	**41.0**	**run3**
ARG9_HB2	GLU916_HG2	1.4	run1	LYS1_NZ	ASP150_OD2	49.1	run3	ARG122_NH2	PHE3_O	3.2	run1
ARG9_HB2	GLU916_HG2	0.3	run2	LYS1_NZ	ASP150_OD2	8.4	run2	ARG122_NH2	PHE3_O	0.7	run2
**PRO919_CD**	**LEU10_HB2**	**47.9**	**run1**	**LYS1_HB2**	**ASP150_O**	**58.9**	**run1**	**ARG9_NH1**	**GLU52_OE1**	**40.2**	**run3**
PRO919_CD	LEU10_HB2	40.8	run3	LYS1_HB2	ASP150_O	0.0	run2	ARG9_NH1	GLU52_OE1	14.7	run1
PRO919_CD	LEU10_HB2	21.4	run2	LYS1_HB2	ASP150_O	0.0	run3	ARG9_NH1	GLU52_OE1	6.7	run2
**TYR7_HE2**	**ASP855_HB2**	**47.4**	**run2**	**SER121_OG**	**ASP4_CG**	**58.8**	**run3**	**ARG9_NH1**	**GLU52_OE2**	**38.5**	**run3**
TYR7_HE2	ASP855_HB2	0.0	run1	SER121_OG	ASP4_CG	22.2	run2	ARG9_NH1	GLU52_OE2	13.4	run1
TYR7_HE2	ASP855_HB2	0.0	run3	SER121_OG	ASP4_CG	0.0	run1	ARG9_NH1	GLU52_OE2	4.5	run2
**PHE917_CA**	**LEU10_HG**	**45.8**	**run3**	**LYS1_HG2**	**ASP150_HB2**	**53.1**	**run1**	**ARG122_HH22**	**ASP4_O**	**36.5**	**run3**
PHE917_CA	LEU10_HG	21.4	run1	LYS1_HG2	ASP150_HB2	0.0	run2	ARG122_HH22	ASP4_O	2.6	run1
PHE917_CA	LEU10_HG	5.6	run2	LYS1_HG2	ASP150_HB2	0.0	run3	ARG122_HH22	ASP4_O	0.0	run2
**ARG9_CB**	**GLU916_HA**	**45.3**	**run3**	**TYR7_HD2**	**VAL314_HA**	**53.0**	**run3**	**ARG122_NH1**	**PHE3_O**	**35.6**	**run2**
ARG9_CB	GLU916_HA	14.9	run1	TYR7_HD2	VAL314_HA	0.4	run2	ARG122_NH1	PHE3_O	17.8	run3
ARG9_CB	GLU916_HA	0.1	run2	TYR7_HD2	VAL314_HA	0.0	run1	ARG122_NH1	PHE3_O	1.1	run1
**TYR7_CB**	**PHE917_HZ**	**43.2**	**run3**	**LYS1_N**	**ASP146_OD1**	**50.0**	**run1**	**SER162_HG1**	**TYR7_HA**	**33.4**	**run1**
TYR7_CB	PHE917_HZ	25.4	run2	LYS1_N	ASP146_OD1	0.0	run2	SER162_HG1	TYR7_HA	8.7	run3
TYR7_CB	PHE917_HZ	23.0	run1	LYS1_N	ASP146_OD1	0.0	run3	SER162_HG1	TYR7_HA	0.1	run2
**LEU10_CG**	**PHE917_HA**	**42.9**	**run3**	**PHE3_HD2**	**ARG216_HA**	**49.9**	**run3**	**HSD113_HB2**	**ILE5_HG11**	**31.9**	**run3**
LEU10_CG	PHE917_HA	22.8	run1	PHE3_HD2	ARG216_HA	0.0	run1	HSD113_HB2	ILE5_HG11	0.0	run1
LEU10_CG	PHE917_HA	9.2	run2	PHE3_HD2	ARG216_HA	0.0	run2	HSD113_HB2	ILE5_HG11	0.0	run2
**ILE5_CG2**	**SER823_HA**	**42.7**	**run3**	**LYS1_N**	**ASP146_OD2**	**46.6**	**run1**	**TYR7_OH**	**MET187_O**	**31.2**	**run2**
ILE5_CG2	SER823_HA	2.0	run1	LYS1_N	ASP146_OD2	0.0	run2	TYR7_OH	MET187_O	0.0	run1
ILE5_CG2	SER823_HA	0.5	run2	LYS1_N	ASP146_OD2	0.0	run3	TYR7_OH	MET187_O	0.0	run3
**LEU10_HB1**	**PRO919_HG1**	**42.5**	**run2**	**LYS1_HA**	**ASP150_O**	**46.5**	**run1**	**ARG122_NH2**	**ASP4_O**	**31.0**	**run3**
LEU10_HB1	PRO919_HG1	19.0	run3	LYS1_HA	ASP150_O	0.0	run2	ARG122_NH2	ASP4_O	9.2	run1
LEU10_HB1	PRO919_HG1	13.2	run1	LYS1_HA	ASP150_O	0.0	run3	ARG122_NH2	ASP4_O	0.2	run2
**LEU10_HD11**	**PHE917_HA**	**42.4**	**run3**	**PHE3_HD2**	**ASN215_O**	**44.6**	**run3**	**ILE5_HB**	**HSD91_HD1**	**30.6**	**run3**
LEU10_HD11	PHE917_HA	14.3	run1	PHE3_HD2	ASN215_O	0.0	run1	ILE5_HB	HSD91_HD1	0.0	run1
LEU10_HD11	PHE917_HA	9.8	run2	PHE3_HD2	ASN215_O	0.0	run2	ILE5_HB	HSD91_HD1	0.0	run2
**GLU916_CA**	**ARG9_HB2**	**42.4**	**run3**	**LYS1_HG1**	**ASP150_CG**	**43.8**	**run1**	**ARG9_NH2**	**GLU52_OE2**	**30.4**	**run1**
GLU916_CA	ARG9_HB2	11.4	run1	LYS1_HG1	ASP150_CG	0.0	run2	ARG9_NH2	GLU52_OE2	26.4	run3
GLU916_CA	ARG9_HB2	0.1	run2	LYS1_HG1	ASP150_CG	0.0	run3	ARG9_NH2	GLU52_OE2	2.1	run2
**LEU10_HN**	**GLU916_C**	**41.4**	**run3**	**LYS1_NZ**	**ASP150_OD1**	**43.5**	**run3**	**SER90_CB**	**ILE5_HB**	**30.0**	**run3**
LEU10_HN	GLU916_C	5.7	run1	LYS1_NZ	ASP150_OD1	12.7	run1	SER90_CB	ILE5_HB	4.1	run2
LEU10_HN	GLU916_C	4.0	run2	LYS1_NZ	ASP150_OD1	11.8	run2	SER90_CB	ILE5_HB	0.0	run1
**TYR7_HH**	**SER730_HB1**	**40.9**	**run3**	**TYR7_HB1**	**VAL314_HB**	**42.6**	**run3**	**ARG9_NH1**	**GLU52_CD**	**30.0**	**run3**
TYR7_HH	SER730_HB1	13.4	run1	TYR7_HB1	VAL314_HB	3.2	run2	ARG9_NH1	GLU52_CD	11.4	run1
TYR7_HH	SER730_HB1	3.2	run2	TYR7_HB1	VAL314_HB	0.0	run1	ARG9_NH1	GLU52_CD	3.9	run2
**SER823_HG1**	**TYR7_HD2**	**40.2**	**run2**	**LYS1_N**	**ASP146_CG**	**41.8**	**run1**	**VAL8_CG2**	**THR285_HB**	**29.8**	**run3**
SER823_HG1	TYR7_HD2	21.9	run3	LYS1_N	ASP146_CG	0.0	run2	VAL8_CG2	THR285_HB	5.7	run1
SER823_HG1	TYR7_HD2	4.2	run1	LYS1_N	ASP146_CG	0.0	run3	VAL8_CG2	THR285_HB	5.6	run2
**LEU10_CB**	**PRO919_HD1**	**40.1**	**run1**	**ASP148_CB**	**PHE3_HE1**	**41.6**	**run3**	**ILE5_CG1**	**PHE88_HB1**	**29.6**	**run3**
LEU10_CB	PRO919_HD1	29.4	run3	ASP148_CB	PHE3_HE1	0.0	run1	ILE5_CG1	PHE88_HB1	2.8	run2
LEU10_CB	PRO919_HD1	20.2	run2	ASP148_CB	PHE3_HE1	0.0	run2	ILE5_CG1	PHE88_HB1	0.4	run1
**LEU10_HN**	**GLU916_O**	**39.8**	**run3**	**ALA2_CB**	**PHE177_HB1**	**41.1**	**run1**	**SER162_HB2**	**TYR7_CE1**	**29.2**	**run3**
LEU10_HN	GLU916_O	9.4	run1	ALA2_CB	PHE177_HB1	0.0	run2	SER162_HB2	TYR7_CE1	1.7	run2
LEU10_HN	GLU916_O	8.2	run2	ALA2_CB	PHE177_HB1	0.0	run3	SER162_HB2	TYR7_CE1	0.5	run1
**LEU10_HD12**	**PHE917_HA**	**39.7**	**run3**	**LYS1_N**	**ASP150_OD1**	**40.2**	**run2**	**ILE5_CB**	**SER90_HB2**	**28.9**	**run3**
LEU10_HD12	PHE917_HA	14.3	run1	LYS1_N	ASP150_OD1	0.0	run1	ILE5_CB	SER90_HB2	5.2	run2
LEU10_HD12	PHE917_HA	8.2	run2	LYS1_N	ASP150_OD1	0.0	run3	ILE5_CB	SER90_HB2	0.1	run1
**SER823_HB1**	**VAL8_HN**	**38.8**	**run1**	**PHE3_HB1**	**ASP217_HA**	**38.7**	**run3**	**ARG122_NE**	**PHE3_O**	**28.4**	**run3**
SER823_HB1	VAL8_HN	21.8	run2	PHE3_HB1	ASP217_HA	0.0	run1	ARG122_NE	PHE3_O	0.0	run1
SER823_HB1	VAL8_HN	0.0	run3	PHE3_HB1	ASP217_HA	0.0	run2	ARG122_NE	PHE3_O	0.0	run2
**SER823_HB2**	**TYR7_HA**	**38.2**	**run1**	**LYS1_HG2**	**ASP150_CB**	**38.5**	**run1**	**LYS11_N**	**THR286_OG1**	**28.2**	**run3**
SER823_HB2	TYR7_HA	34.3	run2	LYS1_HG2	ASP150_CB	0.0	run2	LYS11_N	THR286_OG1	8.6	run1
SER823_HB2	TYR7_HA	5.8	run3	LYS1_HG2	ASP150_CB	0.0	run3	LYS11_N	THR286_OG1	0.0	run2
**LYS1_N**	**ASP4_OD1**	**38.1**	**run1**	**ASP4_HA**	**ALA218_HA**	**37.9**	**run3**	**SER90_OG**	**ASP4_OD2**	**27.7**	**run3**
LYS1_N	ASP4_OD1	19.7	run2	ASP4_HA	ALA218_HA	0.0	run1	SER90_OG	ASP4_OD2	0.0	run1
LYS1_N	ASP4_OD1	0.0	run3	ASP4_HA	ALA218_HA	0.0	run2	SER90_OG	ASP4_OD2	0.0	run2
**ARG9_HB1**	**GLU916_CG**	**38.1**	**run3**	**LYS1_HB2**	**ASP150_C**	**37.6**	**run1**	**ARG122_HH21**	**ASP4_O**	**27.6**	**run3**
ARG9_HB1	GLU916_CG	0.7	run1	LYS1_HB2	ASP150_C	0.0	run2	ARG122_HH21	ASP4_O	5.0	run1
ARG9_HB1	GLU916_CG	0.2	run2	LYS1_HB2	ASP150_C	0.0	run3	ARG122_HH21	ASP4_O	0.0	run2
**LEU10_HD13**	**PHE917_HA**	**37.9**	**run3**	**ALA2_CB**	**ASP150_HB2**	**37.0**	**run1**	**TRP93_NE1**	**TYR7_CZ**	**27.4**	**run3**
LEU10_HD13	PHE917_HA	13.1	run1	ALA2_CB	ASP150_HB2	0.1	run3	TRP93_NE1	TYR7_CZ	5.9	run2
LEU10_HD13	PHE917_HA	7.7	run2	ALA2_CB	ASP150_HB2	0.0	run2	TRP93_NE1	TYR7_CZ	4.5	run1
**LEU10_HG**	**PHE917_HA**	**37.1**	**run3**	**PHE3_CE1**	**ASP148_HB1**	**36.7**	**run3**	**PHE3_N**	**GLN120_O**	**27.4**	**run2**
LEU10_HG	PHE917_HA	18.4	run2	PHE3_CE1	ASP148_HB1	0.0	run1	PHE3_N	GLN120_O	0.0	run1
LEU10_HG	PHE917_HA	10.6	run1	PHE3_CE1	ASP148_HB1	0.0	run2	PHE3_N	GLN120_O	0.0	run3
**LEU10_CD1**	**PHE917_HA**	**36.3**	**run2**	**TYR178_OH**	**LYS1_O**	**36.5**	**run2**	**THR286_HB**	**LYS11_O**	**27.3**	**run3**
LEU10_CD1	PHE917_HA	25.2	run1	TYR178_OH	LYS1_O	0.0	run1	THR286_HB	LYS11_O	20.0	run1
LEU10_CD1	PHE917_HA	22.5	run3	TYR178_OH	LYS1_O	0.0	run3	THR286_HB	LYS11_O	1.0	run2
**GLU916_CB**	**ARG9_HB1**	**35.0**	**run3**	**ARG216_CA**	**PHE3_HD2**	**36.3**	**run3**	**LYS1_N**	**ASP83_OD1**	**27.2**	**run1**
GLU916_CB	ARG9_HB1	18.1	run1	ARG216_CA	PHE3_HD2	0.0	run1	LYS1_N	ASP83_OD1	26.9	run3
GLU916_CB	ARG9_HB1	2.9	run2	ARG216_CA	PHE3_HD2	0.0	run2	LYS1_N	ASP83_OD1	2.0	run2
**SER823_HG1**	**TYR7_HD1**	**34.9**	**run3**	**LYS1_N**	**ASP150_CG**	**35.8**	**run2**	**TYR7_CE1**	**ALA263_O**	**27.1**	**run3**
SER823_HG1	TYR7_HD1	1.8	run1	LYS1_N	ASP150_CG	0.0	run1	TYR7_CE1	ALA263_O	1.0	run2
SER823_HG1	TYR7_HD1	1.3	run2	LYS1_N	ASP150_CG	0.0	run3	TYR7_CE1	ALA263_O	0.2	run1
**VAL8_CG2**	**ASP227_HB2**	**34.4**	**run2**	**LYS1_NZ**	**ASP150_CG**	**35.7**	**run3**	**SER90_OG**	**ASP4_CG**	**26.6**	**run3**
VAL8_CG2	ASP227_HB2	18.8	run1	LYS1_NZ	ASP150_CG	9.9	run2	SER90_OG	ASP4_CG	0.0	run1
VAL8_CG2	ASP227_HB2	0.2	run3	LYS1_NZ	ASP150_CG	7.8	run1	SER90_OG	ASP4_CG	0.0	run2
**LEU10_CD1**	**PRO919_HD1**	**33.5**	**run2**	**ALA218_CA**	**ASP4_HA**	**35.0**	**run3**	**TRP93_NE1**	**TYR7_HH**	**26.3**	**run1**
LEU10_CD1	PRO919_HD1	21.1	run3	ALA218_CA	ASP4_HA	0.4	run2	TRP93_NE1	TYR7_HH	18.7	run3
LEU10_CD1	PRO919_HD1	19.6	run1	ALA218_CA	ASP4_HA	0.0	run1	TRP93_NE1	TYR7_HH	4.6	run2
**ILE225_HA**	**ARG9_O**	**33.1**	**run2**	**TYR178_CE1**	**PHE3_O**	**34.9**	**run2**	**ARG9_NH2**	**GLU52_OE1**	**26.2**	**run1**
ILE225_HA	ARG9_O	1.0	run1	TYR178_CE1	PHE3_O	1.1	run3	ARG9_NH2	GLU52_OE1	23.7	run3
ILE225_HA	ARG9_O	0.1	run3	TYR178_CE1	PHE3_O	0.0	run1	ARG9_NH2	GLU52_OE1	2.4	run2
**ARG9_HB1**	**GLU916_CD**	**32.9**	**run3**	**LYS1_N**	**ASP150_OD2**	**34.7**	**run2**	**TYR7_HE2**	**LYS42_HZ2**	**26.1**	**run3**
ARG9_HB1	GLU916_CD	2.2	run2	LYS1_N	ASP150_OD2	0.0	run1	TYR7_HE2	LYS42_HZ2	0.6	run2
ARG9_HB1	GLU916_CD	0.3	run1	LYS1_N	ASP150_OD2	0.0	run3	TYR7_HE2	LYS42_HZ2	0.0	run1
**LEU10_HB2**	**PRO919_HG1**	**32.2**	**run2**	**SER123_OG**	**ASP4_CB**	**34.5**	**run3**	**ALA2_N**	**ASP84_OD2**	**26.1**	**run3**
LEU10_HB2	PRO919_HG1	6.7	run1	SER123_OG	ASP4_CB	0.0	run1	ALA2_N	ASP84_OD2	4.2	run2
LEU10_HB2	PRO919_HG1	3.5	run3	SER123_OG	ASP4_CB	0.0	run2	ALA2_N	ASP84_OD2	2.2	run1
**SER823_HB2**	**VAL8_HN**	**32.0**	**run1**	**ASP4_HB2**	**SER123_HG1**	**34.4**	**run3**	**SER162_HB1**	**TYR7_HD2**	**25.8**	**run1**
SER823_HB2	VAL8_HN	29.8	run2	ASP4_HB2	SER123_HG1	0.0	run1	SER162_HB1	TYR7_HD2	0.0	run2
SER823_HB2	VAL8_HN	0.1	run3	ASP4_HB2	SER123_HG1	0.0	run2	SER162_HB1	TYR7_HD2	0.0	run3
**ARG9_CB**	**GLU916_HG2**	**32.0**	**run3**	**PHE3_HZ**	**PHE154_HZ**	**33.0**	**run3**	**SER162_HG1**	**VAL8_HN**	**25.5**	**run1**
ARG9_CB	GLU916_HG2	11.5	run1	PHE3_HZ	PHE154_HZ	0.0	run1	SER162_HG1	VAL8_HN	7.5	run3
ARG9_CB	GLU916_HG2	0.2	run2	PHE3_HZ	PHE154_HZ	0.0	run2	SER162_HG1	VAL8_HN	0.0	run2
**LEU10_CD1**	**VAL920_HB**	**31.8**	**run1**	**ALA149_N**	**PHE3_HE1**	**32.8**	**run3**	**GLN267_HB1**	**PHE12_HD1**	**25.5**	**run3**
LEU10_CD1	VAL920_HB	29.5	run3	ALA149_N	PHE3_HE1	0.0	run1	GLN267_HB1	PHE12_HD1	6.0	run1
LEU10_CD1	VAL920_HB	27.5	run2	ALA149_N	PHE3_HE1	0.0	run2	GLN267_HB1	PHE12_HD1	0.0	run2
**LEU10_HB1**	**PRO919_HB2**	**31.3**	**run2**	**LYS1_HE2**	**ASP150_OD2**	**32.6**	**run1**	**ALA2_CB**	**ASP84_HB1**	**25.1**	**run3**
LEU10_HB1	PRO919_HB2	2.4	run3	LYS1_HE2	ASP150_OD2	0.7	run3	ALA2_CB	ASP84_HB1	5.4	run2
LEU10_HB1	PRO919_HB2	0.4	run1	LYS1_HE2	ASP150_OD2	0.0	run2	ALA2_CB	ASP84_HB1	0.7	run1
**LEU10_HD13**	**PRO919_HD1**	**31.2**	**run2**	**TYR7_HD2**	**VAL314_CA**	**31.3**	**run3**	**GLN267_HE21**	**PHE12_N**	**24.7**	**run3**
LEU10_HD13	PRO919_HD1	13.5	run3	TYR7_HD2	VAL314_CA	0.1	run2	GLN267_HE21	PHE12_N	22.8	run1
LEU10_HD13	PRO919_HD1	12.2	run1	TYR7_HD2	VAL314_CA	0.0	run1	GLN267_HE21	PHE12_N	0.6	run2
**ARG9_NH2**	**GLU916_OE2**	**30.8**	**run2**	**PHE3_HE2**	**ARG216_HG2**	**31.0**	**run3**	**SER90_OG**	**ASP4_OD1**	**24.4**	**run3**
ARG9_NH2	GLU916_OE2	22.2	run1	PHE3_HE2	ARG216_HG2	0.1	run2	SER90_OG	ASP4_OD1	0.0	run1
ARG9_NH2	GLU916_OE2	10.7	run3	PHE3_HE2	ARG216_HG2	0.0	run1	SER90_OG	ASP4_OD1	0.0	run2
**LEU10_HB2**	**PRO919_HD1**	**30.7**	**run2**	**TYR7_HN**	**ASN313_HD22**	**29.8**	**run2**	**TYR7_OH**	**THR285_O**	**24.0**	**run2**
LEU10_HB2	PRO919_HD1	25.8	run3	TYR7_HN	ASN313_HD22	2.1	run3	TYR7_OH	THR285_O	1.0	run1
LEU10_HB2	PRO919_HD1	5.8	run1	TYR7_HN	ASN313_HD22	0.0	run1	TYR7_OH	THR285_O	0.0	run3
**LEU10_HD12**	**PRO919_HD1**	**30.6**	**run2**	**PHE3_HB2**	**ASP217_HN**	**29.4**	**run3**	**THR286_CG2**	**LEU10_HA**	**23.9**	**run3**
LEU10_HD12	PRO919_HD1	12.1	run3	PHE3_HB2	ASP217_HN	0.0	run1	THR286_CG2	LEU10_HA	21.1	run1
LEU10_HD12	PRO919_HD1	10.9	run1	PHE3_HB2	ASP217_HN	0.0	run2	THR286_CG2	LEU10_HA	5.8	run2
**SER823_HA**	**TYR7_HD2**	**30.2**	**run1**	**SER123_HB1**	**ASP4_OD1**	**28.7**	**run3**	**LYS1_N**	**ASP83_CG**	**23.8**	**run3**
SER823_HA	TYR7_HD2	0.9	run2	SER123_HB1	ASP4_OD1	0.5	run2	LYS1_N	ASP83_CG	18.4	run1
SER823_HA	TYR7_HD2	0.3	run3	SER123_HB1	ASP4_OD1	0.0	run1	LYS1_N	ASP83_CG	2.2	run2
**TYR7_CA**	**SER823_HB2**	**30.1**	**run1**	**LYS1_HG1**	**ASP150_OD2**	**28.2**	**run1**	**GLN267_HE21**	**PHE12_HA**	**23.4**	**run3**
TYR7_CA	SER823_HB2	15.1	run2	LYS1_HG1	ASP150_OD2	0.0	run2	GLN267_HE21	PHE12_HA	18.3	run1
TYR7_CA	SER823_HB2	0.9	run3	LYS1_HG1	ASP150_OD2	0.0	run3	GLN267_HE21	PHE12_HA	1.9	run2
**SER823_HA**	**TYR7_HE1**	**30.1**	**run3**	**PHE3_CE1**	**ALA149_HN**	**28.0**	**run3**	**TYR7_CA**	**SER162_HG1**	**22.9**	**run1**
SER823_HA	TYR7_HE1	0.2	run1	PHE3_CE1	ALA149_HN	0.0	run1	TYR7_CA	SER162_HG1	4.2	run3
SER823_HA	TYR7_HE1	0.0	run2	PHE3_CE1	ALA149_HN	0.0	run2	TYR7_CA	SER162_HG1	0.0	run2
**ARG9_CB**	**GLU916_HB1**	**30.0**	**run3**	**TYR7_HD2**	**ASN313_O**	**27.9**	**run2**	**VAL8_CG1**	**THR285_HB**	**22.7**	**run3**
ARG9_CB	GLU916_HB1	20.6	run1	TYR7_HD2	ASN313_O	7.1	run3	VAL8_CG1	THR285_HB	12.0	run1
ARG9_CB	GLU916_HB1	4.0	run2	TYR7_HD2	ASN313_O	0.0	run1	VAL8_CG1	THR285_HB	9.0	run2
**ARG9_NH2**	**GLU916_OE1**	**29.6**	**run2**	**LYS1_NZ**	**ASP218_OD2**	**27.5**	**run2**	**TYR7_HH**	**THR286_HA**	**22.5**	**run3**
ARG9_NH2	GLU916_OE1	23.9	run1	LYS1_NZ	ASP218_OD2	0.0	run1	TYR7_HH	THR286_HA	0.3	run1
ARG9_NH2	GLU916_OE1	9.6	run3	LYS1_NZ	ASP218_OD2	0.0	run3	TYR7_HH	THR286_HA	0.0	run2
**SER823_HA**	**TYR7_CE1**	**29.5**	**run3**	**ALA2_HB1**	**TYR178_HD1**	**27.5**	**run1**	**ILE5_HG21**	**HSD91_HD1**	**22.2**	**run3**
SER823_HA	TYR7_CE1	0.0	run1	ALA2_HB1	TYR178_HD1	0.0	run2	ILE5_HG21	HSD91_HD1	0.0	run1
SER823_HA	TYR7_CE1	0.0	run2	ALA2_HB1	TYR178_HD1	0.0	run3	ILE5_HG21	HSD91_HD1	0.0	run2
**TYR7_HB2**	**PHE917_HZ**	**28.7**	**run2**	**LYS1_N**	**ASP179_CG**	**27.3**	**run3**	**ARG9_NH2**	**GLU52_CD**	**22.2**	**run1**
TYR7_HB2	PHE917_HZ	13.9	run1	LYS1_N	ASP179_CG	0.0	run1	ARG9_NH2	GLU52_CD	12.1	run3
TYR7_HB2	PHE917_HZ	1.0	run3	LYS1_N	ASP179_CG	0.0	run2	ARG9_NH2	GLU52_CD	0.7	run2
**TYR7_CA**	**SER823_HG1**	**28.6**	**run1**	**ASP4_CA**	**ALA218_HA**	**27.3**	**run3**	**VAL8_N**	**SER162_OG**	**22.1**	**run1**
TYR7_CA	SER823_HG1	0.9	run2	ASP4_CA	ALA218_HA	0.1	run2	VAL8_N	SER162_OG	2.5	run3
TYR7_CA	SER823_HG1	0.0	run3	ASP4_CA	ALA218_HA	0.0	run1	VAL8_N	SER162_OG	0.0	run2
**LEU10_HD11**	**PRO919_HD1**	**28.4**	**run2**	**PHE3_HB2**	**ASP217_N**	**26.8**	**run3**	**SER162_HG1**	**TYR7_HD1**	**21.9**	**run3**
[illegible]	[illegible]	[illegible]	[illegible]	[illegible]	[illegible]	[illegible]	[illegible]	[illegible]	[illegible]	[illegible]	[illegible]

Table S2. Hydrogen bonding occupancy during the MD simulations. Hydrogen bond analysis obtained from molecular dynamics simulations replicates. The table lists all donor–acceptor pairs detected during the simulation, together with their corresponding occupancy (%) over the trajectory and the average donor–acceptor distance (Å).
