# Supplementary figures and images for: Beyond MIDAS: An In Silico Study of a Putative Noncanonical C16 Binding Site in αvβ3 Integrin
Source: ACS Omega. 2026 Feb 19;11(8):13606–28. doi: 10.1021/acsomega.5c11287 (PMC12961469; doi:10.1021/acsomega.5c11287)

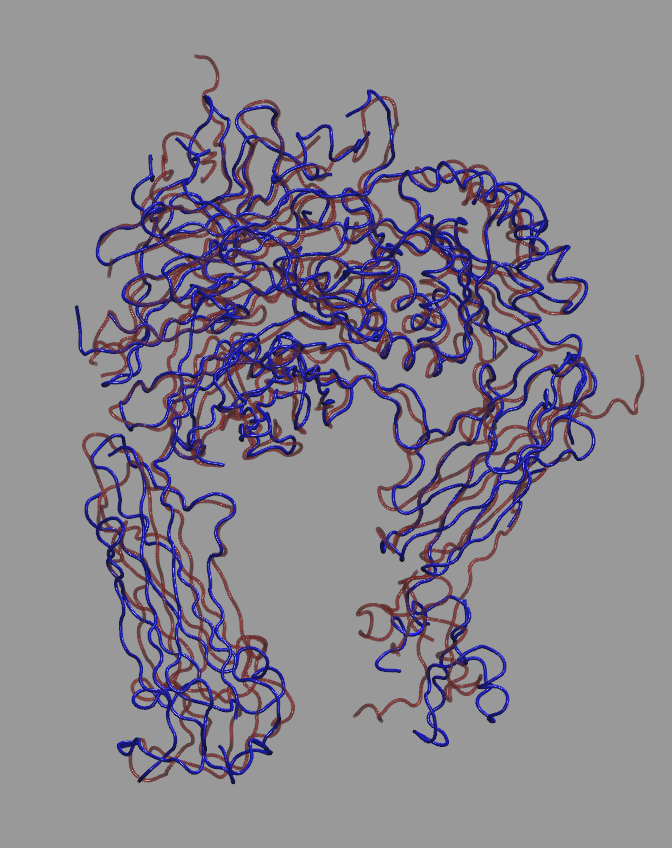

Supplement: Supplementary file 2 [file ao5c11287_si_002.zip › def_vec.gif]

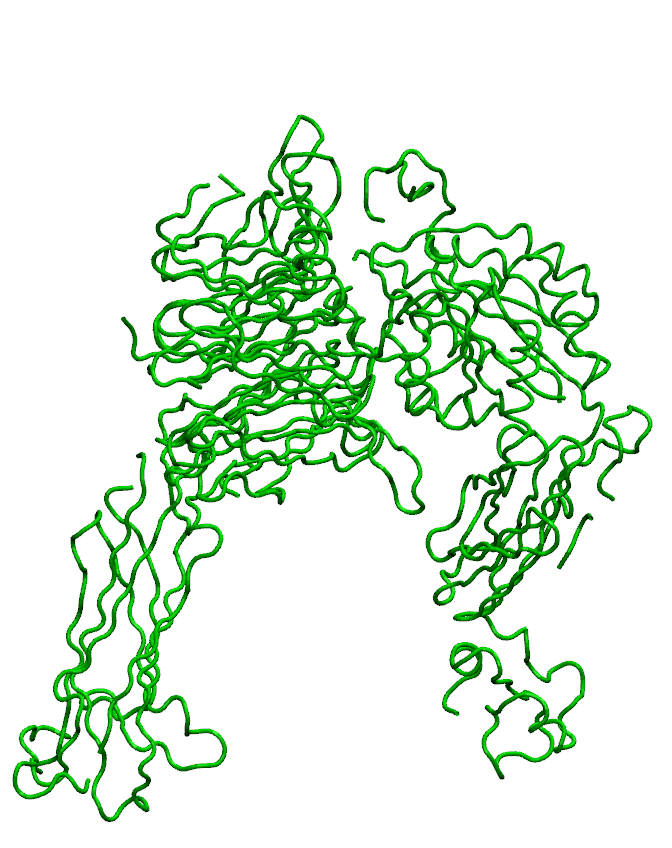

Supplement: Supplementary file 2 [file ao5c11287_si_002.zip › mode_1.gif]

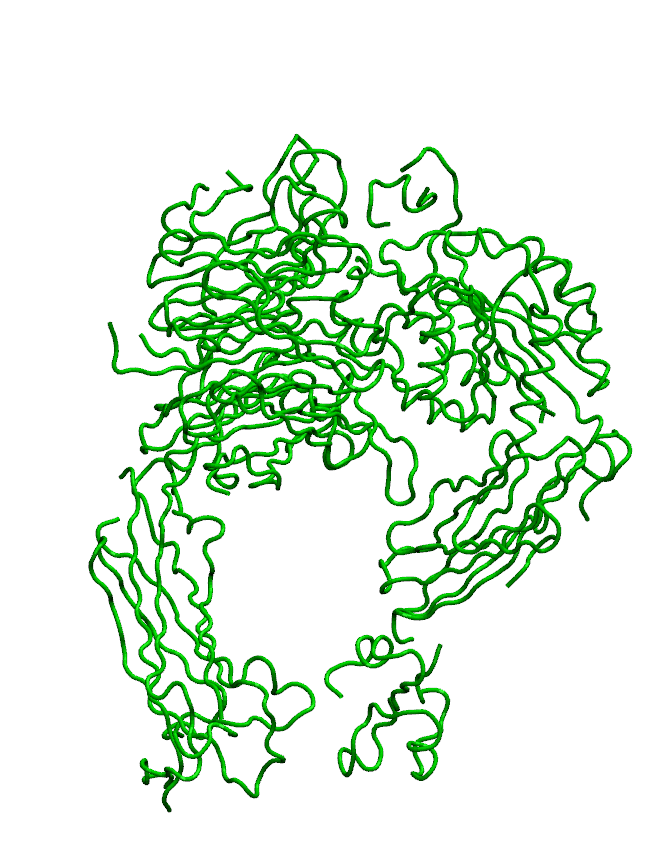

Supplement: Supplementary file 2 [file ao5c11287_si_002.zip › mode_2.gif]

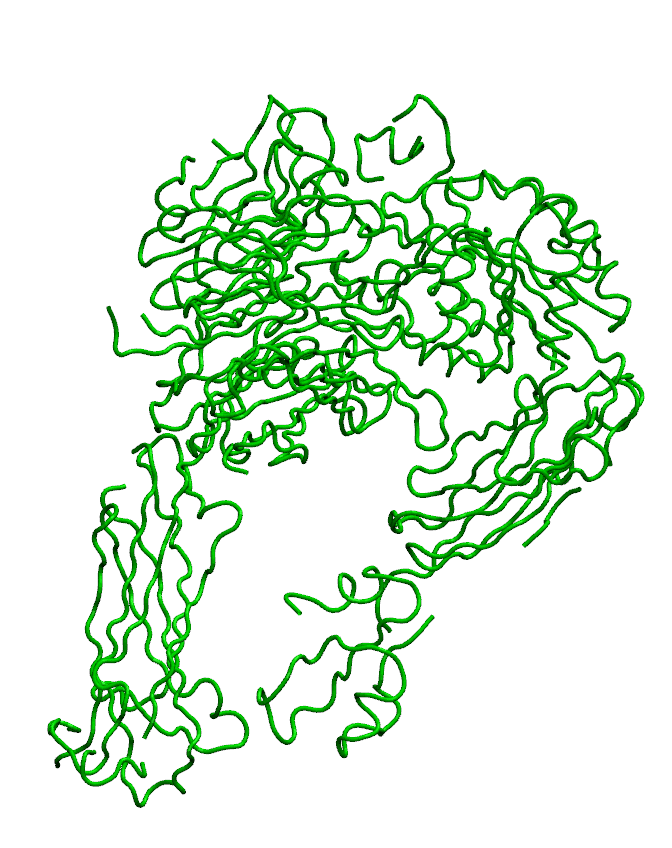

Supplement: Supplementary file 2 [file ao5c11287_si_002.zip › mode_3.gif]

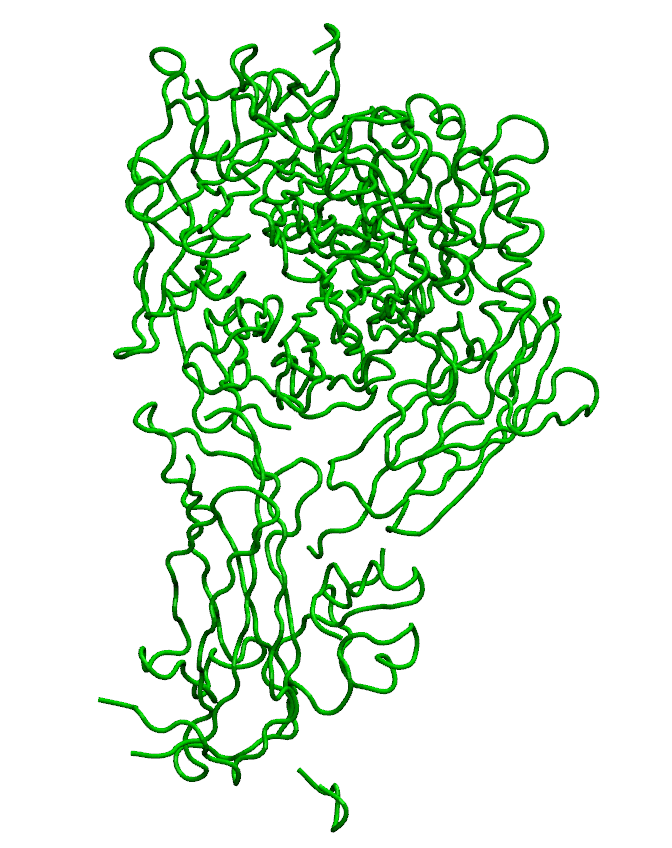

Supplement: Supplementary file 2 [file ao5c11287_si_002.zip › mode_4.gif]

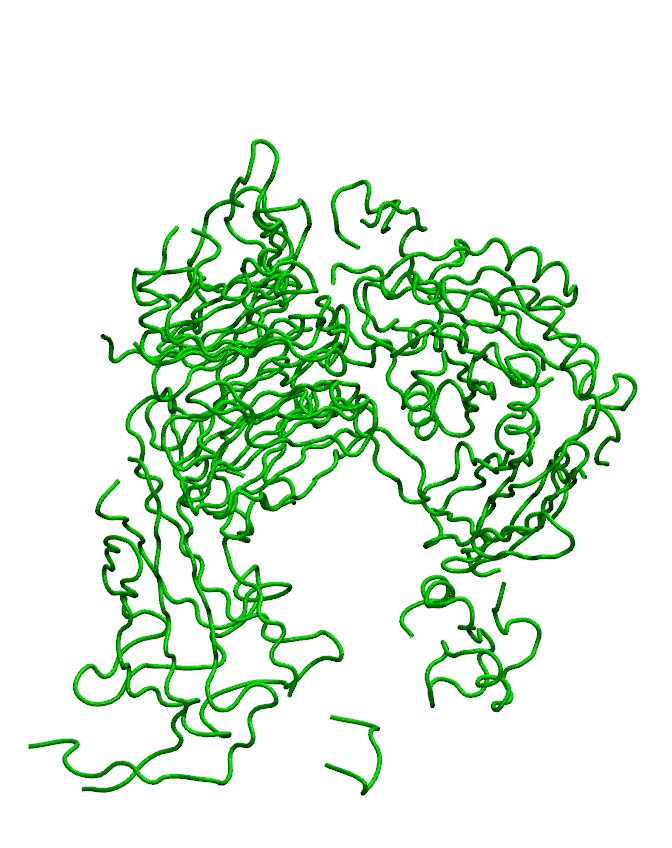

Supplement: Supplementary file 2 [file ao5c11287_si_002.zip › mode_5.gif]

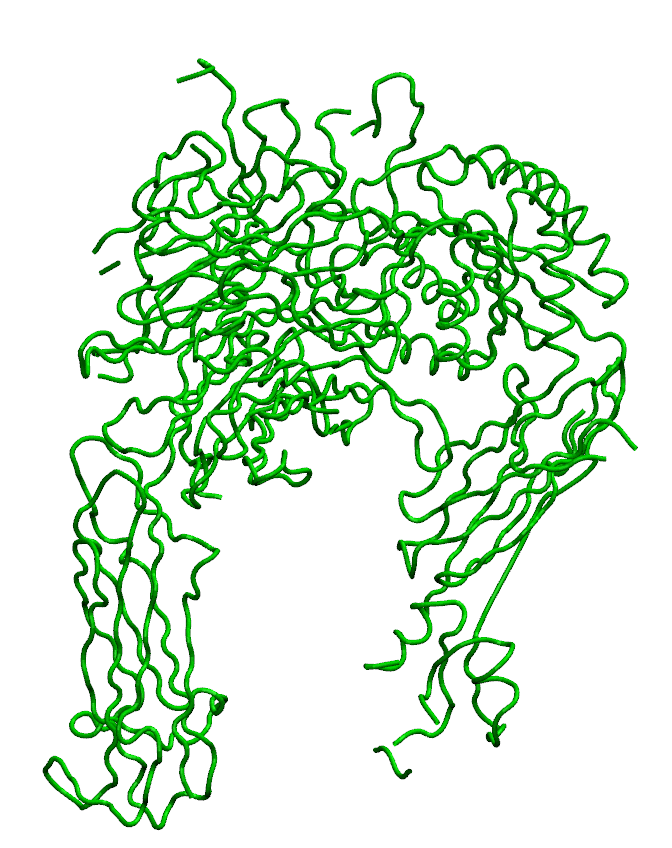

Supplement: Supplementary file 2 [file ao5c11287_si_002.zip › pc1.gif]

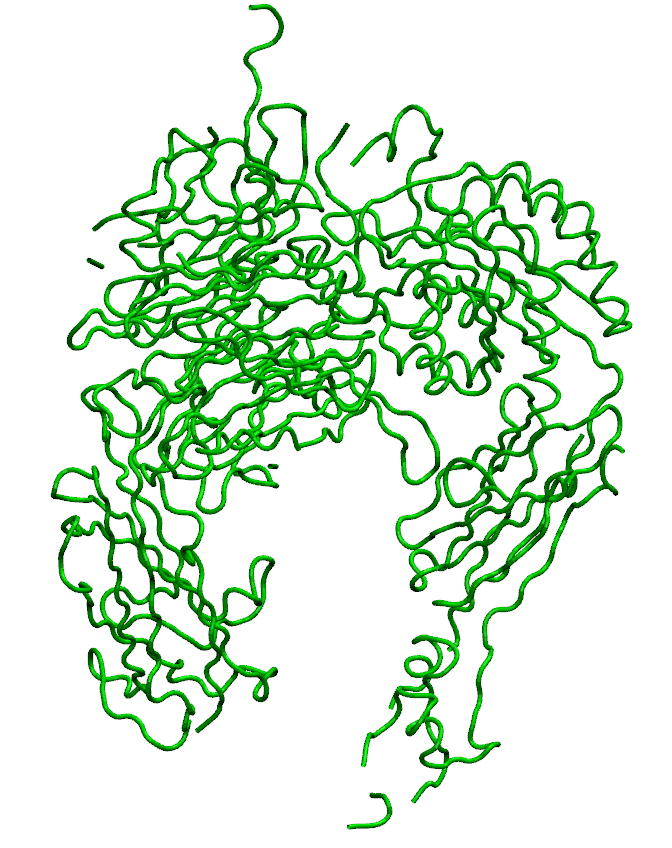

Supplement: Supplementary file 2 [file ao5c11287_si_002.zip › pc2.gif]

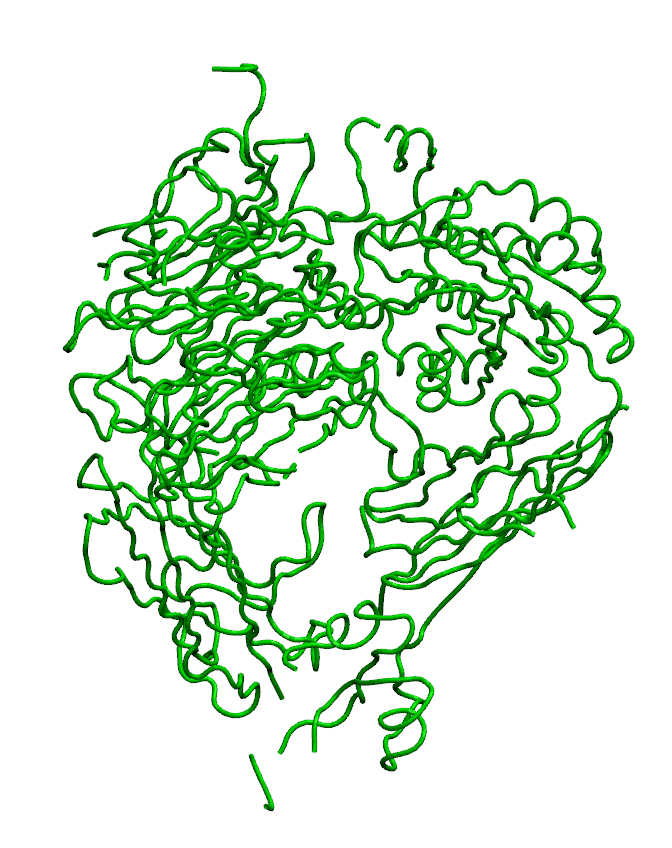

Supplement: Supplementary file 2 [file ao5c11287_si_002.zip › pc3.gif]

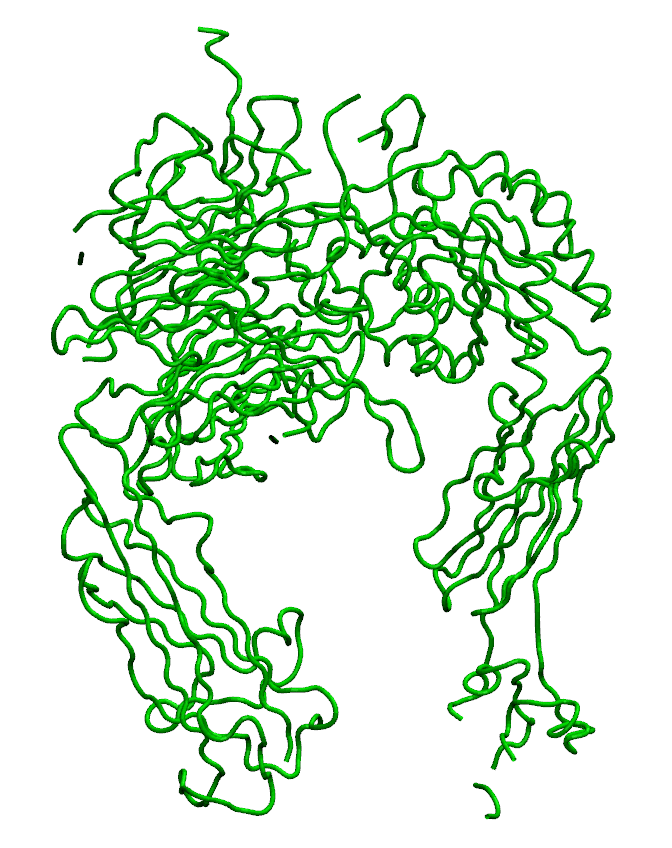

Supplement: Supplementary file 2 [file ao5c11287_si_002.zip › pc4.gif]

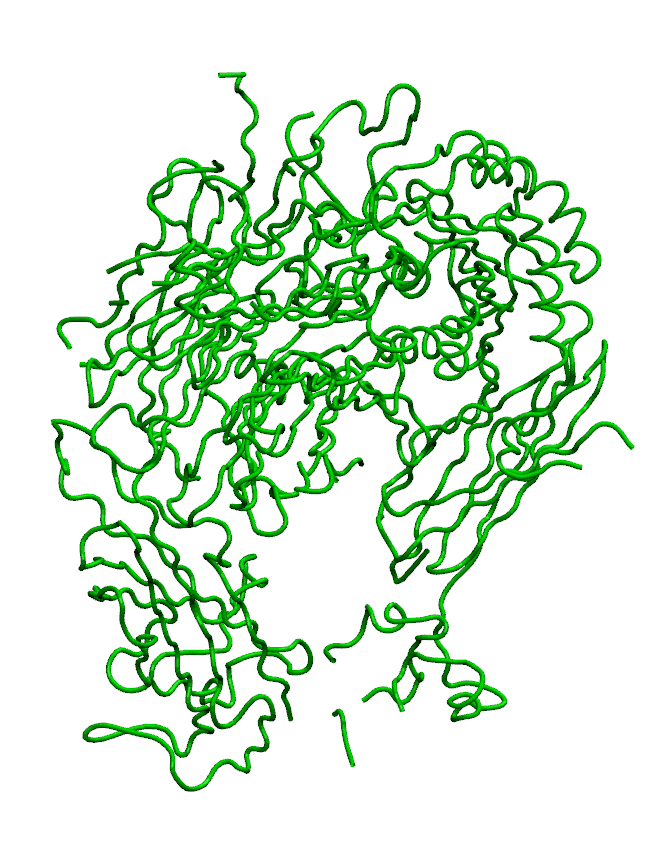

Supplement: Supplementary file 2 [file ao5c11287_si_002.zip › pc5.gif]

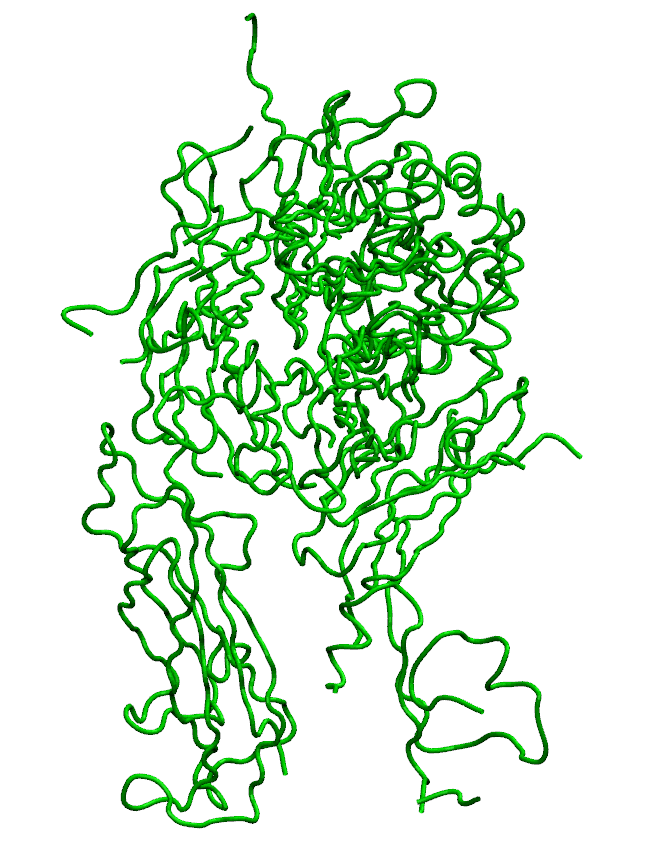

Supplement: Supplementary file 2 [file ao5c11287_si_002.zip › pc6.gif]

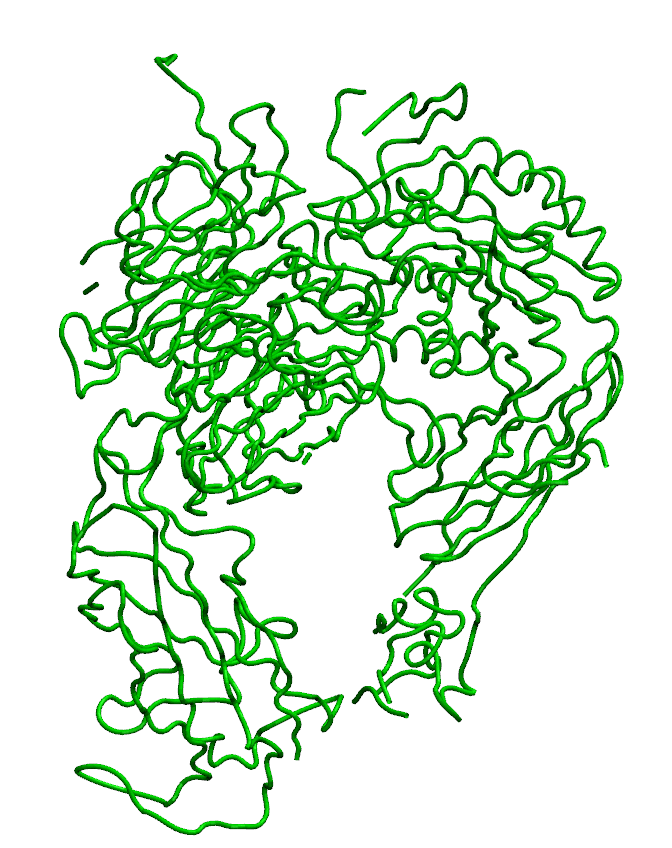

Supplement: Supplementary file 2 [file ao5c11287_si_002.zip › pc7.gif]
